# FoxO1-zDHHC4-CD36 S-Acylation Axis Drives Metabolic Dysfunction in Diabetes

**DOI:** 10.1161/CIRCRESAHA.124.325918

**Published:** 2025-05-13

**Authors:** Kaitlyn M.J.H. Dennis, Keshav Gopal, Claudia N. Montes Aparicio, Jiashuo Aaron Zhang, Marcos Castro-Guarda, Thomas Nicol, Ríona M. Devereux, Ryan D. Carter, Saara-Anne Azizi, Tong Lan, Ujang Purnama, Carolyn A. Carr, Gul Simsek, Eleanor K. Gill, Pawel Swietach, Oana Sorop, Ilkka H.A. Heinonen, Francesco Schianchi, Joost J.F.P. Luiken, Dunja Aksentijevic, Dirk J. Duncker, Bryan C. Dickinson, Sarah De Val, John R. Ussher, William Fuller, Lisa C. Heather

**Affiliations:** 1 Department of Physiology, Anatomy and Genetics (K.M.J.H.D., C.N.M.A., J.A.Z., M.C.-G., T.N., R.M.D., R.D.C., U.P., C.A.C., G.S., E.K.G., P.S., S.D.V., L.C.H.), University of Oxford, United Kingdom.; 2 Department of Chemistry (R.M.D.), University of Oxford, United Kingdom.; 3 Faculty of Pharmacy and Pharmaceutical Sciences, University of Alberta, Edmonton, Canada (K.G., J.R.U.).; 4 Department of Chemistry, University of Chicago, IL (S.-A.A., T.L., B.C.D.).; 5 Division of Experimental Cardiology, Department of Cardiology, Erasmus MC, University Medical Center Rotterdam, the Netherlands (O.S., I.H.A.H., D.J.D.).; 6 Turku PET Centre, University of Turku and Turku University Hospital, Finland (I.H.A.H.).; 7 Faculty of Health, Medicine and Life Sciences, Department of Genetics and Cell Biology, Maastricht University, the Netherlands (F.S., J.J.F.P.L.).; 8 Barts and the London Faculty of Medicine and Dentistry, William Harvey Research Institute, Queen Mary University of London, United Kingdom (D.A.).; 9 School of Cardiovascular and Metabolic Health, University of Glasgow, United Kingdom (W.F.).

**Keywords:** cardiovascular diseases, diabetic cardiomyopathies, heart failure, insulin resistance, myocardial infarction

## Abstract

**BACKGROUND:**

The fatty acid (FA) transporter CD36 (FA translocase/cluster of differentiation 36) is the gatekeeper of cardiac FA metabolism. Preferential localisation of CD36 to the sarcolemma is one of the initiating cellular responses in the development of muscle insulin resistance and in the type 2 diabetic heart. Post-translational S-acylation controls protein trafficking, and in this study we hypothesised that increased CD36 S-acylation may underpin the preferential sarcolemmal localisation of CD36, driving metabolic and contractile dysfunction in diabetes.

**METHODS:**

Type 2 diabetes was induced in the rat using high fat diet and a low dose of streptozotocin. Forkhead box O1 (FoxO1) transcriptional regulation of zDHHC4 (zinc finger DHHC-type palmitoyltransferase 4) and subsequent S-acylation of CD36 was assessed using chromatin immunoprecipitation (ChIP) sequencing, ChIP-quantitative polymerase chain reaction, luciferase assays, siRNA (small interfering RNA) and shRNA silencing.

**RESULTS:**

Type 2 diabetes increased cardiac CD36 S-acylation, CD36 sarcolemmal localisation, FA oxidation rates and triglyceride storage in the diabetic heart. CD36 S-acylation was increased in diabetic rats, *db/db* mice, diabetic pigs and insulin-resistant human iPSC-derived cardiomyocytes, demonstrating conservation between species. The enzyme responsible for S-acylating CD36, zDHHC4, was transcriptionally upregulated in the diabetic heart, and genetic silencing of zDHHC4 decreased CD36 S-acylation. We identified that *zDHHC4* expression is under the regulation of the transcription factor FoxO1. Diabetic mice with cardiomyocyte-specific FoxO1 deletion had decreased cardiac *zDHHC4* expression and decreased CD36 S-acylation, which was further confirmed using diabetic mice treated with the FoxO1 inhibitor AS1842856. Pharmacological inhibition of zDHHC enzymes in diabetic hearts decreased CD36 S-acylation, sarcolemmal CD36 content, FA oxidation rates and triglyceride storage, culminating in improved cardiac function in diabetes. Conversely, inhibiting the de-acylating enzymes in control hearts increased CD36 S-acylation, sarcolemmal CD36 content and FA metabolic rates in control hearts, recapitulating the metabolic phenotype seen in diabetic hearts.

**CONCLUSIONS:**

Activation of the FoxO1-zDHHC4-CD36 S-acylation axis drives metabolic and contractile dysfunction in the type 2 diabetic heart.

**Novelty and Significance**

**What Is Known?**

- Metabolic dysfunction drives diabetic cardiomyopathy; however, the underlying mechanisms still remain elusive.
- Changes in fatty acid metabolism occur early on during disease progression.
- Understanding the mechanism responsible for increased cardiac fatty acid uptake in diabetes is critical, as this is the primary regulated step in fatty acid metabolism in the heart.

**What New Information Does This Article Contribute?**

- In diabetes, CD36 (fatty acid translocase/cluster of differentiation 36), the fatty acid transporter, is redistributed to the sarcolemma due to increased posttranslational S-acylation, mediated by increased expression of the S-acylating enzyme zDHHC4 (zinc finger DHHC-type palmitoyltransferase 4).
- The transcription factor FoxO1 (forkhead box O1) drives increased expression of zDHHC4, mediating increased CD36 S-acylation in diabetes.
- Pharmacologically inhibiting CD36 S-acylation corrects fatty acid metabolism and lipotoxicity, and improves cardiac function in diabetes.

Excessive fatty acid metabolism within the diabetic myocardium contributes to contractile dysfunction and disease progression; therefore, it is paramount to identify the mechanisms driving this overdependence on fatty acids. CD36 is the sarcolemmal fatty acid transporter responsible for the majority of fatty acid uptake into the heart and, as such, is often referred to as the metabolic gatekeeper of cardiac fatty acid metabolism. In this article, we show that CD36 is redistributed to the sarcolemma from intracellular vesicle stores in diabetes due to increased post-translational S-acylation. This relocation increases fatty acid uptake, oxidation, and lipid storage in the diabetic heart. Increased CD36 S-acylation is mediated by increased expression of the S-acylating enzyme zDHHC4 in diabetes. The transcription factor FoxO1 drives increased transcription of zDHHC4, and deletion of FoxO1 reverses CD36 S-acylation in diabetes. Pharmacologically inhibiting CD36 S-acylation corrects fatty acid metabolism and lipotoxicity, and improves cardiac function in diabetes. Taken together, a FoxO1-zDHHC4-CD36 S-acylation axis exists in diabetes, which drives fatty acid metabolic dysfunction and contributes to contractile dysfunction. As sarcolemmal CD36, the metabolic gatekeeper, influences all intracellular pathways utilizing or regulated by myocardial fatty acids, this work paves the way toward the development of new cardiometabolic therapies in diabetes.

**Meet the First Author, see p 1523 | Editorial, see p 1561**

Cardiovascular disease is the leading cause of mortality in patients with type 2 diabetes (T2D) due to the development of diabetic cardiomyopathy, increased incidence of myocardial infarction, and accelerated progression of heart failure. There is a growing body of research demonstrating that abnormal cardiac metabolism is a key mechanism driving cardiac dysfunction in diabetes.^1^ Diabetes causes a metabolic shift within the heart, increasing fatty acid (FA) oxidation and myocardial triglyceride storage, with concomitant decreases in glucose oxidation and glycolysis.^2,3^ As such, T2D is a systemic metabolic disorder associated with a cardiac pathological overdependence on FA utilization.^4^

The primary step in cardiac FA metabolism is uptake across the sarcolemma, and the FA transporter CD36 (FA translocase/cluster of differentiation 36) is responsible for the majority of FA uptake into the heart.^5^ CD36 is an integral membrane protein that translocates from intracellular endosomes to the sarcolemma in response to stimuli, to increase FA uptake and fuel onward metabolism.^6^ In diabetes, CD36 is preferentially relocated to the sarcolemma mediating increased FA uptake, with increased sarcolemmal localization identified as one of the earliest changes in the development of insulin resistance in skeletal muscle.^7^ Therefore, metabolic dysfunction in diabetes is associated with abnormal CD36 trafficking, though the mechanisms driving this remain unclear.

Recently, a lipid post-translational modification, S-acylation, which regulates membrane trafficking of various proteins, has sparked interest in the field of metabolism. S-acylation involves the covalent addition of a long chain FA, most commonly palmitate, to a cysteine thiol through a thioester bond^8^ (also referred to as S-palmitoylation). S-acylated proteins can undergo reversible cycles of S-acylation and de-S-acylation with timescales ranging from seconds to hours.^8^ The S-acylation reaction is catalyzed by zDHHC (zinc finger DHHC-type palmitoyltransferase) enzymes, and the de-S-acylation reaction is catalyzed by a family of enzymes including APTs (acyl protein thioesterases) and ABHD (α/β-hydrolase domain) enzymes.^8^ S-acylation of CD36 regulates its trafficking in adipocytes.^9,10^

This study sets out to investigate if the S-acylation of CD36 is a driving force for metabolic and contractile dysfunction in the diabetic heart and, critically, if pharmacologically targeting the S-acylation enzymes can reverse dysfunction in diabetes. Using a combination of rodent, pig, and human tissues in combination with genetic and pharmacological in vivo and ex vivo approaches, we demonstrate increased CD36 S-acylation in diabetes, which is conserved between species and is sufficient to drive metabolic and contractile dysfunction. We identify a novel FoxO (forkhead box O) 1-zDHHC4-CD36 S-acylation axis, which causes relocation of CD36 to the sarcolemma, increasing FA metabolism and decreasing cardiac function. Critically, we demonstrate that pharmacological inhibition of zDHHC activity directly, or indirectly via FoxO1 inhibition and decreased zDHHC4 (zinc finger DHHC-type palmitoyltransferase 4) expression, normalizes CD36 S-acylation and localization, FA metabolism, and cardiac function in T2D. Taken together, we demonstrate that activation of the FoxO1-zDHHC4-CD36 S-acylation axis drives metabolic and contractile dysfunction in type 2 diabetic heart, which can be reversed by pharmacologically targeting the S-acylation enzymes.

## Methods

### Data Availability

The data that support the findings of this study are available from the corresponding author upon reasonable request.

### Rat Model of T2D

T2D was induced as previously described, generating an early stage model of the disease presenting with mild hyperglycemia, hyperlipidemia, and hyperinsulinemia.^11,12^ Briefly, male Wistar rats (starting body weight ≈300 g; Envigo) were fed a high-fat diet (cat. no. 829197, 60% calories from fats; Special Diet Services) for 5 weeks, and on day 14, they received a single low-dose intraperitoneal injection of streptozotocin (25-mg/kg body weight). Control rats were fed a standard chow diet for 5 weeks. Experiments conformed to the Home Office Guidance on the Operation of the Animals (Scientific Procedures) Act, 1986, and were approved by a local ethics committee (University of Oxford, United Kingdom). Males have a higher prevalence of T2D (estimated at 17.7 million more males than females worldwide),^13^ so this study focused on the effect in male animals.

### Mouse Models of T2D

Snap-frozen cardiac tissues from male 13-week C57BL/KsJ-lepr^*db*^/lepr^*db*^ (*db/db*) and lean control heterozygote (*db/*+) mice (Charles River, Italy) were investigated. αMHC^Cre^ (alpha-myosin heavy chain Cre; The Jackson Laboratory) and cardiac-specific FoxO1 deficient (*Foxo1*^fl/fl^ αMHC^Cre^, hereafter referred to as *Foxo1*^Cardiac−/−^) littermates on a C57BL/6J background were generated as previously described.^14^ αMHC^Cre^ mice were either fed a chow diet (αMHC^Cre^ control) or a high-fat diet (60% kcal from lard, Research Diets D12492) for 10 weeks with a single intraperitoneal injection of streptozotocin (75-mg/kg body weight) at 4 weeks (αMHC^Cre^ diabetic). A subset of these diabetic mice received twice daily oral gavage with the FoxO1 inhibitor AS1842856 (100 mg/kg; MedChemExpress LLC) for the final 2 weeks of the dietary protocol (αMHC^Cre^ diabetic+AS1842856). Cardiac-specific FoxO1 deficient (*Foxo1*^[Cardiac^^−/−]^) mice^14^ were either fed chow or underwent the same high-fat diet/single intraperitoneal injection of streptozotocin to induce diabetes as the littermate αMHC^Cre^ mice, to give models of both pharmacological and genetic FoxO1 inhibition on the same genetic and diabetes background. Neither *Foxo1*^(Cardiac−/−)^ mice nor AS1842856-treated mice showed any difference in body weight or food intake compared with their respective control.^14^

### Porcine Model of T2D

Frozen heart tissues (left ventricular free wall) from diabetic and control adult male Göttingen minipigs were studied.^15^ Briefly, diabetes was induced in the swine by intravenous injections of streptozotocin (25 mg/kg per day) over 3 days. One week later, the swine were fed a high-fat and high-sugar diet (25% saturated fats, 10% sucrose, and 15% fructose) for 5 months, whereas the healthy control swine consumed normal pig chow.^15^

### Insulin-Resistant Human Induced Pluripotent Stem Cell-Derived Cardiomyocyte

Human induced pluripotent stem cells (IMR90) were differentiated into cardiomyocytes and matured using our previously published protocol.^16,17^ Insulin resistance was induced in the matured contracting human induced pluripotent stem cell-derived cardiomyocyte (hiPSC-CM) as previously described^17^ and characterized.^18^ Briefly, mature hiPSC-CMs were cultured for 3 days in glucose-free insulin resistance media comprising DMEM no glucose, supplemented with 0.3-mmol/L palmitic acid:bovine serum albumin (BSA) (bound 6:1), 1.7-µmol/L insulin, 5-µg/mL vitamin B12, 0.5-mmol/L vitamin C, 0.84-µmol/L biotin, 1X nonessential amino acids, 0.1X penicillin/streptomycin, and 10% horse serum. On day 4, the media was switched to insulin resistance media as described above but also containing 12-mmol/L glucose and 3.4-µmol/L insulin for an additional 3 days.

### Isolated Heart Perfusion

Hearts were isolated and arrested in ice-cold Krebs-Henseleit buffer, rapidly cannulated via the aorta, and then perfused in retrograde Langendorff mode according to our published protocol.^12,19^ A fluid-filled PVC balloon connected to a pressure transducer was inserted into the left ventricle and inflated to give an end-diastolic pressure of 4 to 8 mm Hg, and the left ventricle contracted against a constant afterload pressure of 100 mm Hg. Left ventricular developed pressure was calculated as peak systolic minus end-diastolic pressure, and rate pressure product was calculated as the developed pressure multiplied by the heart rate. Hearts were perfused with recirculating Krebs-Henseleit buffer supplemented with 11-mmol/L glucose and 0.4-mmol/L palmitate (bound to BSA), gassed with 95% O_2_ and 5% CO_2_, and maintained at 37 °C. For measurement of palmitate oxidation rates, this buffer was supplemented with 0.2-µCi/mL [9,10-^3^H] palmitate. Buffer aliquots were collected at 4-minute intervals throughout the perfusion, and the conversion of ^3^H-palmitate into ^3^H_2_O was measured following Folch extraction, with the upper aqueous phase counted for radioactivity. For the low- and high-fat perfusions, the palmitate concentration of the KH buffer was decreased to 0.2 mmol/L and increased to 1.2 mmol/L, respectively, for 60 minutes.^20^ For the cyano-myracrylamide^21^ (CMA; 100 µmol/L) and ML348 (N-[2-chloro-5-(trifluoromethyl)phenyl]-4-(2-furanylcarbonyl)-1-piperazineacetamide) (100 µmol/L) perfusion experiments, the compounds were dissolved in DMSO (dimethyl sulfoxide) and added into the perfusion buffer after cardiac function had been stable for 10 minutes, and recirculated for a further hour. T2D rat hearts were perfused with CMA, and control rat hearts were perfused with ML348. Following perfusion, hearts were freeze-clamped on the cannula for subsequent analysis.

### Acyl Resin–Assisted Capture

S-acylated proteins were purified from snap-frozen tissue using acyl resin–assisted capture^22^; 10 mg of tissue was incubated in blocking buffer (2.5% SDS [sodium dodecyl sulfate] [w/v], 100-mmol/L HEPES [2-(4-2-hydroxyethyl)piperazin-1-yl ethanesulfonic acid], 1-mmol/L EDTA [ethylenediaminetetraacetic acid], and pH 7.4) and free cysteines alkylated by addition of 100-mmol/L N-ethylmaleimide and incubated at 40 °C for 4 hours. Excess maleimide was removed by acetone precipitation; protein pellets were washed with 70% acetone (v/v), dried, and resolubilized in binding buffer (1% SDS [w/v], 100-mmol/L HEPES, 1-mmol/L EDTA, and pH 7.4). S-acylated proteins were captured on thiopropyl sepharose resin in the presence of 200-mmol/L hydroxylamine (pH 7.4) for 2.5 hours at room temperature. An identical reaction in which hydroxylamine was replaced with 200-mmol/L NaCl served as a negative control. Following the capture of acylated proteins, the beads were extensively washed in binding buffer, and proteins were eluted by heating for 10 minutes at 60 °C in SDS-PAGE (polyacrylamide gel electrophoresis) loading buffer supplemented with 100-mmol/L DTT. The input fraction, negative control (−HA [minus hydroxylamine]), and S-acylated (+HA) fractions were analyzed to assess the enrichment of the S-acylated fraction, by dividing the +HA fractions by the input fraction.

### Acyl-PEG Exchange

Acyl-PEG (polyethylene glycol) exchange was performed using an established protocol.^23^ Briefly, frozen tissue was incubated in blocking buffer (2.5% SDS [w/v], 100-mmol/L HEPES, 1-mmol/L EDTA, and pH 7.4) and free cysteines alkylated by incubation with 100-mmol/L N-ethylmaleimide at 40 °C for 4 hours. Excess maleimide was removed by acetone precipitation, and protein pellets were washed with 70% (v/v) acetone, dried, and then resolubilized in binding buffer (1% SDS [w/v], 100-mmol/L HEPES, 1-mmol/L EDTA, and pH 7.4). Acylated proteins were PEGylated using 2-mmol/L 5-kDa PEG maleimide in the presence of 200-mmol/L HA (pH 7.4) for 2 hours at room temperature. An identical reaction in which HA was replaced with 200-mmol/L NaCl served as a negative control.

### Subcellular Fractionation

Separation of the sarcolemmal membrane fraction from intracellular endosomes (low-density microsomal fraction) was performed as previously described.^24,25^ Briefly, cardiac tissue was incubated in a high-salt solution (20-mmol/L HEPES, 2-M NaCl, and 5-mmol/L NaN_3_), followed by centrifugation, resuspension in fractionation buffer (20-mmol/L HEPES, 250-mmol/L sucrose, 2-mmol/L EDTA, 1-mmol/L MgCl_2_, and 5-mmol/L NaN_3_), and homogenization using a glass hand-held homogenizer. Differential centrifugation was used to separate the different membrane fractions.

### Molecular Analyses

Cardiac triglyceride concentrations were measured using a Randox triglyceride assay kit following Folch extraction.^11^ RNA was extracted from cells and tissue with a RNeasy mini kit (QIAGEN), with cDNA conversion performed with a high-capacity RNA-to-cDNA kit (Applied Biosystems), using a SensoQuest Labcycler (Geneflow). Quantitative polymerase chain reaction (PCR) amplification was performed with Power SYBR Green PCR Master Mix with 15 ng/well of cDNA using a StepOnePlus Real-Time PCR System machine (Applied Biosystems). Relative gene expression was calculated using the 2^−ΔΔCt^ method, normalized to the housekeeper gene (Table S1).

For Western blotting analyses, tissue was lysed in ice-cold lysis buffer, and 10-μg protein was loaded onto SDS-PAGE gels and separated by electrophoresis. Primary antibodies were used (Table S2), in combination with the relevant secondary antibodies (Abcam). Even protein loading and transfer were confirmed using ponceau S total protein staining as a housekeeping loading control, and blots were imaged using a Chemidoc XRS+ imaging system (Bio-Rad) and Image Studio Software.

### Bioinformatic Analysis of the zDHHC4 Promotor

Binding motifs for the FoxO1 transcription factor around the *zDHHC4* promoter were identified using the JASPAR Transcription Factors Track Settings (minimum score, 300) on the University of California Santa Cruz (UCSC) browser using mouse sequence GRCm38/mm10 and rat sequence RGSC 5.0/rn5 sequence.^26,27^ FoxO1 transcription factor binding data (assessed by chromatin immunoprecipitation [ChIP]-seq) in the adult mouse heart were previously published^28^ and deposited on GEO with accession number GSM4278011. Enriched H3K4Me3 (histone H3 lysine 4 trimethylation) binding (assessed by ChIP-seq) in mouse cardiomyocytes was previously published^29^ and deposited on GEO with accession number GSM5255561. Both data sets were visualized using IGV (integrative genomics viewer).^30^

### RNA Sequencing

RNA was extracted from hiPSC-CM using the Qiagen RNeasy Mini kit following the manufacturer’s protocol and sequenced using an Illumina stranded paired 150-bp poly A enrichment protocol. FastQC (v 0.12.1) was used with default settings to assess read quality and check the trimming of sequencing adaptors. Trimming of Illumina adaptors and removal of low-quality reads (q<20) was performed with trim-galore (v 0.6.10). Salmon (v 1.10.2) tools were used for transcript quantification using the GRCh38 primary assembly.^31^ For differential expression analysis and normalization of gene-level counts, the DESEQ2^32^ pipeline was used. Gene set enrichment analysis was performed using the FGSEA^33^ package, with genes ranked by the Wald test statistic from the differential expression analysis performed with DESEQ2 between control and insulin-resistant hiPSC-CMs. The transcription factor targets pathway from msigDB^34^ (molecular signatures database) evaluated FOXO1 signaling. The genes determined to contribute to the enrichment (leading edge) were extracted and plotted as normalized and scaled data to visualize each pathway on a gene level. The gene set variation analysis (GSVA) algorithm^35^ calculated single-sample level scores for the *FOXO1* targets. Data are available and deposited on GEO with accession number GSE288708.

### ChIP-Quantitative PCR and Luciferase

The EZ ChIP kit (EMD Millipore) was used according to the manufacturer’s protocol. Briefly, differentiated H9c2 (H9c2 myoblasts) cardiac myocytes were transfected with *FoxO1* wild-type (WT) plasmid for 24 hours using Lipofectamine 2000 and treated with 1% formaldehyde to cross-link proteins to DNA following our previously published method.^36^ Cells were lysed with protease inhibitors, sonicated to shear DNA into fragments, and incubated with antibodies against FoxO1 or IgG (immunoglobulin G; negative control) overnight. The purified DNA and input genomic DNA were analyzed by real-time PCR (Table S1).

Mouse zDHHC4 promoter (−2000 to +100) was synthesized (Biobasic, Inc, Canada) and cloned in PGL3 (pGL3 luciferase reporter vector) basic vector at Sac I (sac I restriction site) and *Hin*dIII sites. Differentiated H9c2 cardiac myocytes were transfected with either *FoxO1* WT (Addgene plasmid 12148), *FoxO3* WT (Addgene plasmid 8360), or *FoxO4* WT (Addgene plasmid 17549) plasmids along with *zDHHC4* promoter-luciferase construct for 24 hrs using Lipofectamine 2000 (Thermo) as per manufacturer’s instructions. *FoxO1* WT and *FoxO4* WT were gifts from Domenico Accili (Addgene plasmid 12148 and Addgene plasmid 17549).^37^ FoxO3 WT was a gift from Michael Greenberg (Addgene plasmid 8360).^38^ Cells were lysed in reporter lysis buffer, and a luciferase assay was performed using the bright-glow luciferase assay system (Promega Corporation) as per the manufacturer’s instructions. The luciferase activity was normalized to the total protein amount and presented relative to the empty vector used for overexpression.

### Cell Silencing of FoxO1 and zDHHC4

FoxO1 and zDHHC4 were silenced in matured hiPSC-CM using 50 nmol/L of the SMARTPool *FOXO1* siRNA (small interfering RNA) (L-003006-00-0005) or *ZDHHC4* siRNA (L-016699-01-0005 Dharmacon) compared with nontarget pool (D-001810-10-05) siRNA using DharmaFECT 1 transfection reagent, and cells were collected 48 hours post-transfection. FoxO1 was also silenced in mouse endothelial sEnd.1 (skin derived endothelial cells 1) cells^39^ using 50 nM of the SMARTPool *Foxo1* siRNA (L-041127-00-0005) for 24 hours. zDHHC4 was also silenced in neonatal rat ventricular myocytes using LVRU6GH lentiviral particles containing either scrambled control sequence (control scramble shRNA) or *zDHHC4*-specific shRNA (*zDHHC4* shRNA; Genecopoeia). Neonatal rat ventricular myocytes were isolated and cultured according to our previously published protocol.^40^ Cells were transduced with viral particles at an MOI of 10 with polybrene at a final concentration of 10 μg/mL, and were harvested for acyl resin–assisted capture 4 days post-transduction.

### Statistics

Results are either presented as means±SEM or median±95% confidence limit on the median and were considered significant at *P*<0.05, analyzed (except where otherwise specified) with GraphPad Prism Version 10. Data were tested for normality in GraphPad using the Shapiro-Wilk test when n>5. For comparisons between 2 groups, normally distributed data were analyzed using a 2-tailed unpaired *t* test. If data were not normally distributed or n<5, it was analyzed using a nonparametric Mann-Whitney *U* test. For data comparing >2 groups either a nonparametric equivalent of the 1-way ANOVA, the Kruskal-Wallis test (with the Dunn multiple comparison post hoc test) was performed in Prism, or for 2-by-2 factorial designs, an aligned ranks transform-based nonparametric ANOVA (with post hoc pairwise comparisons with correction using the Benjamini-Hochberg procedure) was performed in R (version 4.2.0) utilizing the ARTool package.^41–43^ The Aligned Ranks Transform is reported, in contrast to other methods of undertaking a nonparametric ANOVA such as the Scheirer Ray Hare test, to have less inflation of type 1 error and greater statistical power.^44^ We have estimated post hoc pairwise contrasts through the method described by Elkin et al,^45^ again with the false discovery rate controlled by the Benjamini-Hochberg procedure. For these rank-based multigroup analyses, data are presented as median±95% CI.

## Results

### Increased FA Metabolism in Diabetes Is Associated With Increased Sarcolemmal CD36

**Figure 1.**
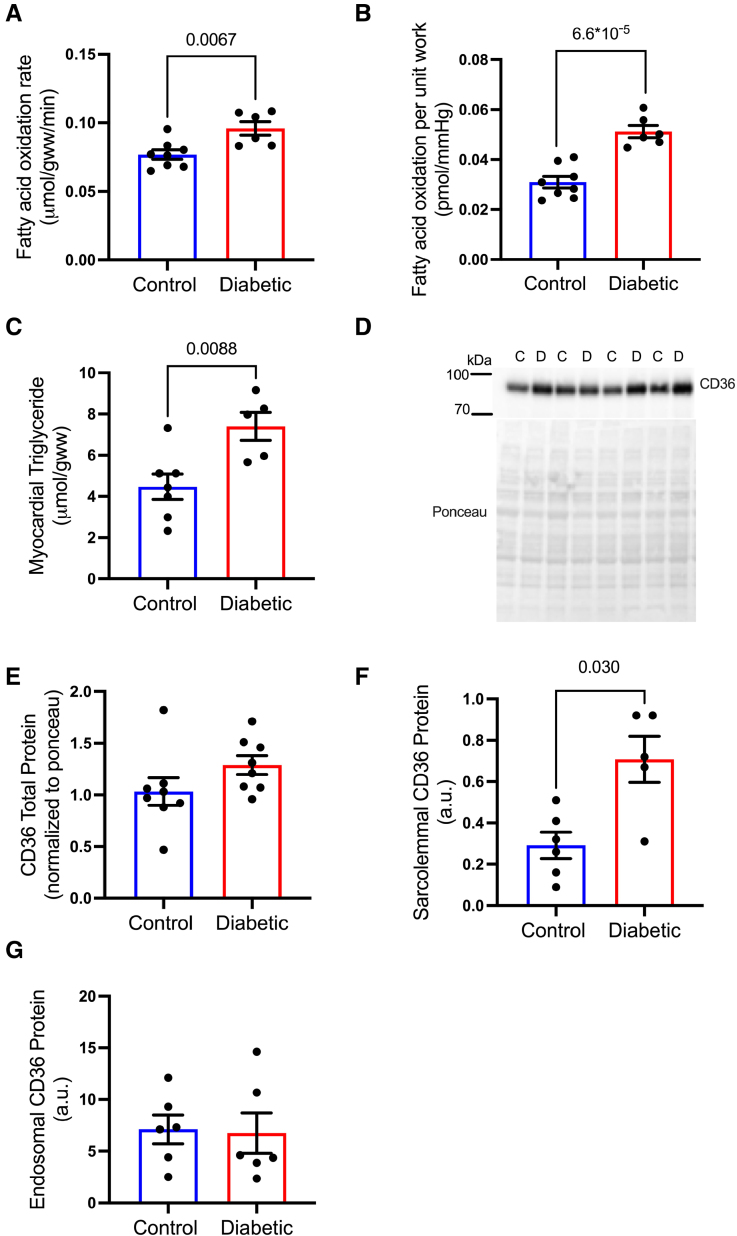
**Diabetes increases cardiac fatty acid metabolism associated with increased sarcolemmal CD36 (fatty acid translocase/cluster of differentiation 36).** Diabetic hearts had increased fatty acid oxidation rates (**A**), fatty acid oxidation per unit work (**B**), and myocardial triglyceride concentrations (**C**) compared with control hearts. Total CD36 protein (**D** and **E**) was not significantly increased, but sarcolemma content of CD36 was increased in diabetic hearts compared with controls (**F**), with no significant differences in endosomal CD36 content (**G**). Data (**A**, **B**, **E**, and **G**) were compared using a 2-tailed unpaired *t* test and (**C** and **F**) were compared using a Mann-Whitney *U* test (data show the mean±SEM).

Inducing T2D in the rat using a combination of high-fat diet and low-dose streptozotocin resulted in a mild model of T2D characterized by a 36% increase in fasting blood glucose, a 2-fold increase in nonesterified FA concentrations, and a 44% increase in fasting insulin concentrations, accompanied by increased adiposity with no significant differences in heart weight (Table S3). Perfusing hearts in contracting Langendorff mode with ^3^H-palmitate demonstrated that FA oxidation rates were increased by 25% in diabetic hearts (Figure 1A), resulting in a 66% increase in FA oxidation per unit work (Figure 1B; Figure S1), compared with control hearts. Furthermore, myocardial triglyceride concentrations were elevated by 65% in diabetes compared with controls (Figure 1C). CD36 is the predominant FA transporter responsible for importing FAs into the cardiomyocyte. Total CD36 protein levels were not significantly different between groups, though were trending upward in the diabetic hearts (Figure 1D and 1E). Subcellular fractionation of hearts demonstrated that the sarcolemmal content of CD36 was increased 2.2-fold (Figure 1F) in hearts from diabetic rats compared with controls, with no significant differences observed in CD36 within the endosomal compartment (Figure 1G). These results demonstrate that there is a preferential localization of CD36 protein to the sarcolemma in diabetes, facilitating increased FA uptake to fuel increased FA oxidation and storage.

### CD36 S-Acylation Is Increased in Diabetes and Conserved Between Species

**Figure 2.**
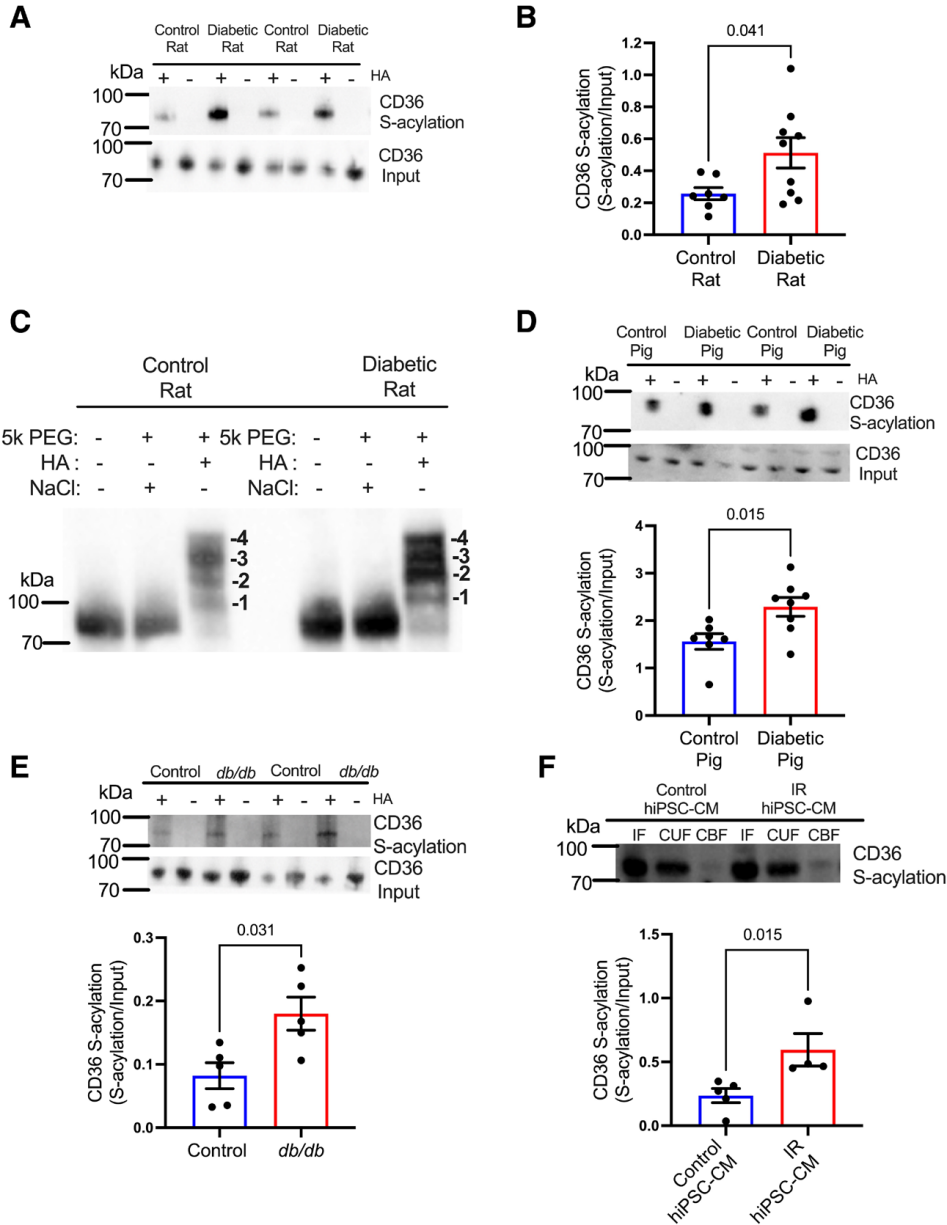
**Diabetes increases cardiac CD36 (fatty acid translocase/cluster of differentiation 36) S-acylation across species.** Diabetic rats had increased cardiac CD36 S-acylation (**A** and **B**) compared with controls. **C**, CD36 is S-acylated on 4 cysteine residues in hearts from control and diabetic rats. Cardiac CD36 S-acylation is increased in diabetic pigs (**D**), *db/db* mice (**E**), and insulin-resistant human induced pluripotent stem cell-derived cardiomyocyte (hiPSC-CM) (**F**) compared with their respective controls. Data (**B** and **D**) were compared using a 2-tailed unpaired *t* test and (**E** and **F**) compared using a Mann-Whitney *U* test (data show the mean±SEM). +HA or CBF indicates S-acylated protein (hydroxylamine); CUF, unacylated protein; −HA, negative control (minus hydroxylamine); and IF, input fraction.

The post-translational modification S-acylation mediates membrane trafficking of various proteins; therefore, we investigated if increased sarcolemmal CD36 in diabetic hearts was associated with increased S-acylation. Using acyl resin–assisted capture, we measured CD36 S-acylation in diabetic and control hearts and normalized this to CD36 in the input fraction (to correct for changes in total CD36 protein). CD36 S-acylation was increased 2-fold in the hearts of diabetic rats compared with controls after normalization for CD36 content (Figure 2A and 2B). Thus, a greater percentage of the CD36 pool is S-acylated in diabetic rat hearts than in controls. Furthermore, using acyl-PEG, it was determined that CD36 is S-acylated on up to 4 cysteine residues (Figure 2C) in control and diabetic hearts. No evidence of S-acylation was observed for the other FA transport protein FABPpm (fatty acid binding protein plasma membrane) or the glucose transporters GLUT1 (glucose transporter 1) and GLUT4 (glucose transporter 4) (Figure S2), suggesting that CD36 is selectively S-acylated among the main substrate transporters in the rat heart.

To determine whether the increase in CD36 S-acylation was conserved across species and between models of T2D, we investigated diabetic pigs, mice, and insulin-resistant human cardiomyocytes. CD36 S-acylation was increased by 47% in diabetic pig hearts (Figure 2D), increased by 2-fold in leptin receptor-deficient *db/db* mouse hearts (Figure 2E), and increased by 2.5-fold in insulin-resistant hiPSC-CMs (Figure 2F) compared with their respective controls.

### Diabetes Increases the Expression of the S-Acylating Enzyme zDHHC4

**Figure 3.**
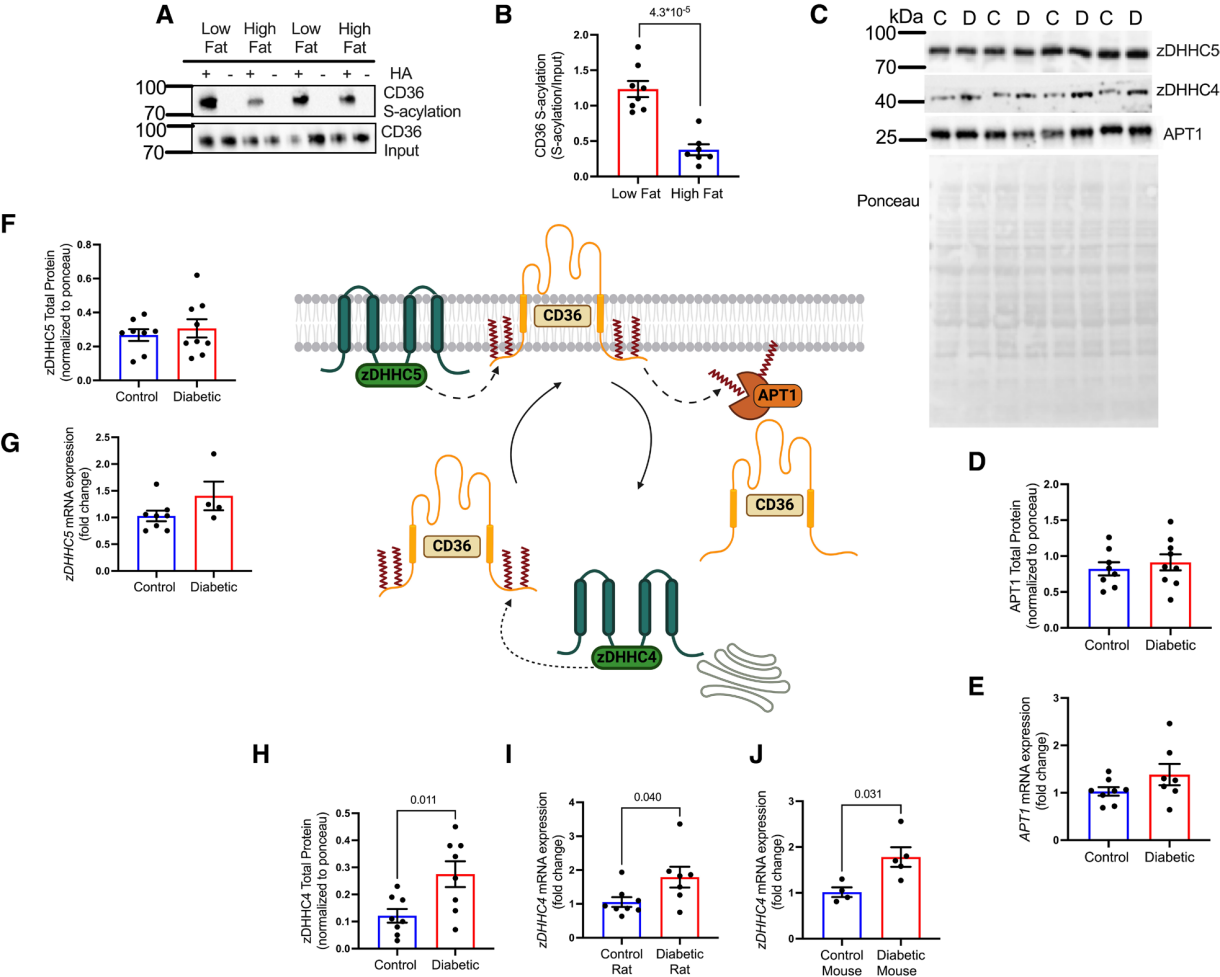
**Diabetes increases the expression of the S-acylating enzyme zDHHC4 (zinc finger DHHC-type palmitoyltransferase 4). A** and **B**, Perfusion with a high-fat buffer decreased CD36 (fatty acid translocase/cluster of differentiation 36) S-acylation compared with low-fat perfused heart. **C**, Representative Western blot images showing zDHHC4, zDHHC5 (zinc finger DHHC-type palmitoyltransferase 5), and APT1 (Acyl protein thioesterase 1) protein levels in control and diabetic hearts. APT1 (**D** and **E**) and zDHHC5 (**F** and **G**) protein and mRNA expressions were not significantly different between control and diabetic hearts. **H**, In contrast, zDHHC4 total protein was significantly increased in diabetic hearts compared with controls. *zDHHC4* mRNA from diabetic rats (**I**) and diabetic mice (**J**) were significantly increased compared with their respective controls. Data (**B**–**F**, **H**, and **I**) were compared using a 2-tailed unpaired *t* test and (**G** and **J**) were compared using a Mann-Whitney *U* test (data show the mean±SEM). +HA indicates S-acylated protein (hydroxylamine); and −HA, negative control (minus hydroxylamine).

We next investigated the mechanism driving increased CD36 S-acylation in diabetes. As palmitoyl-CoA (coenzyme A) is the main substrate for S-acylation, we investigated whether acutely increasing palmitate supply to the heart would increase CD36 S-acylation. Healthy rat hearts were perfused for 1 hour with low (0.2 mM) or high (1.2 mM) palmitate, causing no significant differences in cardiac function between groups (Figure S3). FA oxidation rates were increased 3.8-fold in the high-fat perfused hearts (Figure S3F). However, CD36 S-acylation significantly decreased in the high-fat perfused cohort compared with the low-fat group (Figure 3A and 3B), independently of changes in CAV (caveolin) 3 S-acylation (Figure S3B). Therefore, an increase in substrate availability for the zDHHCs does not directly drive enhanced CD36 S-acylation in intact beating hearts.

The main regulatory enzymes mediating CD36 S-acylation are zDHHC4 and zDHHC5 (zinc finger DHHC-type palmitoyltransferase 5),^10^ and the main de-S-acylating enzyme is APT1 (Acyl protein thioesterase 1)^9^; therefore, the expression of these proteins was investigated in diabetes (Figure 3C). APT1 displayed no significant differences in total protein or mRNA expression between control and diabetes (Figure 3D and 3E). Similarly, zDHHC5, which tethers CD36 to the sarcolemma and protects it from de-S-acylation, showed no significant differences in total protein or mRNA expression between diabetic and control hearts (Figure 3F and 3G). In contrast, zDHHC4, which S-acylates CD36 at the Golgi to target it for trafficking to the sarcolemma, displayed a 2.4-fold increase in protein abundance (Figure 3H) and a 73% increase in mRNA expression in diabetic hearts compared with controls (Figure 3I). These findings were recapitulated in a mouse model of T2D where *zDHHC4* mRNA was increased by 82% in diabetic hearts compared with controls (Figure 3J).

### The Transcription Factor FoxO1 Drives Enhanced zDHHC4 Expression

**Figure 4.**
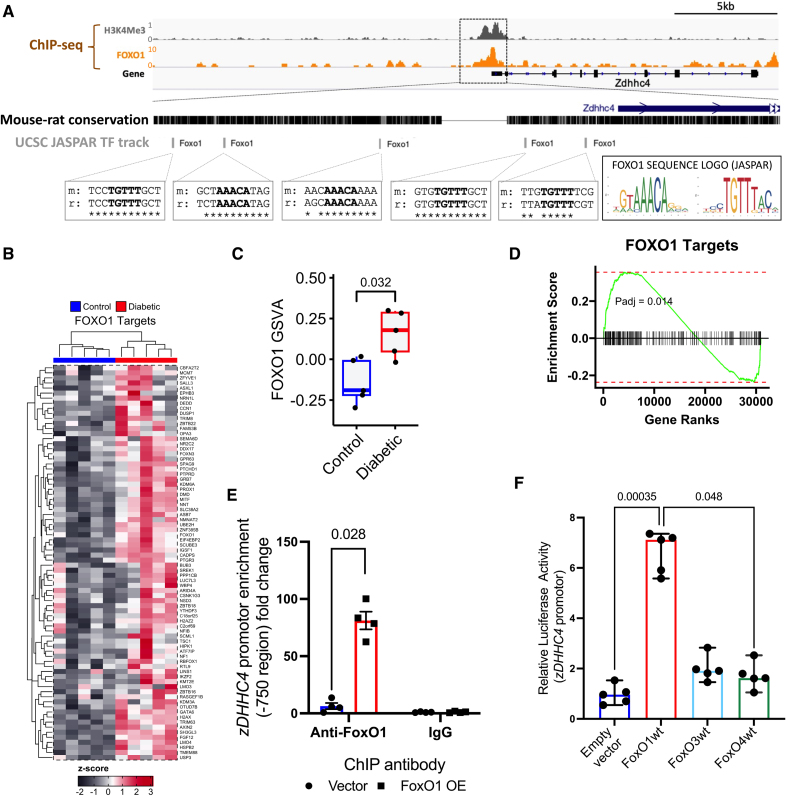
**The transcription factor FoxO1 (forkhead box O1) drives enhanced z*DHHC4* (zinc finger DHHC-type palmitoyltransferase 4) expression. A**, Genomic regions around the mouse z*DHHC4* gene alongside tracks showing chromatin immunoprecipitation (ChIP)-seq signal for the promoter mark H3K4Me3 in mouse cardiomyocytes and the FoxO1 transcription factor in adult mouse heart. JASPAR transcription factor track on University of California Santa Cruz (UCSC) browser identified 5 mouse-rat conserved FoxO1 binding motifs within the promoter region (core motif in bold alongside the JASPAR sequence logo for FoxO1 in both orientations; **A**). FoxO1 target gene enrichment scores (**D**) and heat map visualization of FoxO1 target genes demonstrate clustering between control and insulin-resistant human induced pluripotent stem cell-derived cardiomyocyte (hiPSC-CM; **B**). **C**, Single-sample enrichment scores for the FoxO1 pathway for the genes belonging to the FoxO1 pathway were increased in insulin-resistant hiPSC-CM compared with controls. **E**, FoxO1 binds to the *zDHHC4* promotor in H9c2 (H9c2 myoblasts) cardiomyocytes measured by chromatin immunoprecipitation/quantitative (q)PCR, which is absent in the IgG (immunoglobulin G) control group. **F**, Transfection of a luciferase reporter construct encoding the *zDHHC4* promoter into H9c2 cardiomyocytes demonstrated increased luciferase activity in the presence of the *FoxO1* wild-type (WT) plasmid relative to empty vector and *FoxO4* WT plasmid. Data (**C** and **E**) were compared using a Mann-Whitney *U* test (data show the mean±SEM). Data (**D**) present the Benjamini and Hochberg false discovery rate (FDR)–corrected *P* value for the enrichment of FOXO (controlling the FDR for the 1115 total pathways included). Data (**F**) were compared using a Kruskal-Wallis test with the Dunn multiple comparison post hoc test (data show the median±95% CI).

To identify the transcription factor responsible for increased *zDHHC4* expression in diabetes, we examined its promotor region using the JASPAR Transcription Factor Track Settings, an open-access database of transcription factors binding profiles, visualized on the UCSC browser (Figure 4A). This identified 5 binding motifs for the FoxO1 transcription factor conserved within both mouse and rat *zDHHC4* promotor sequences. Furthermore, examination of ChIP-seq data sets in adult mouse hearts^28^ also revealed an enrichment of direct FoxO1 binding over the *zDHHC4* promotor.

Differential expression analysis was used to capture the global differences in gene expression within our insulin-resistant hiPSC-CM model. The FOXO1 targets pathway was significantly enriched in genes ranked by the global differential expression comparison (Figure 4C and 4D). Visualizing the genes contributing to this enrichment demonstrates observable differences and robustly clusters the control and insulin-resistant groups (Figure 4B). Furthermore, calculating single-sample enrichment scores for the FOXO1 pathway confirms significantly increased expression for this pathway in the insulin-resistant hiPSC-CMs for the genes belonging to the pathway (Figure 4C). Thus, FoxO1 target genes are upregulated in the insulin-resistant state.

To confirm the binding of FoxO1 to the *zDHHC4* promotor, we performed ChIP-quantitative PCR experiments in H9c2 cardiomyocytes overexpressing empty vector or FoxO1, using primers designed to the *zDHHC4* promotor that in silico analysis had identified as a potential FoxO1 binding region. This demonstrated significant 80-fold enrichment of the *zDHHC4* promotor following FoxO1 immunoprecipitation in the FoxO1 overexpressing cardiomyocytes, with negligible enrichment using IgG control antibody (Figure 4E). In addition, we transfected H9c2 cardiac myocytes with *FoxO1* WT plasmid along with a luciferase reporter construct encoding the *zDHHC4* promoter. We found that *FoxO1* overexpression significantly increased luciferase activity 6-fold using this *zDHHC4* reporter construct compared with the empty vector control (Figure 4F). Conversely, transfection of a plasmid encoding for other *FoxO4* WT overexpression did not increase luciferase activity via the *zDHHC4* reporter construct.

### A FoxO1-zDHHC4-CD36 S-Acylation Axis in Diabetes

**Figure 5.**
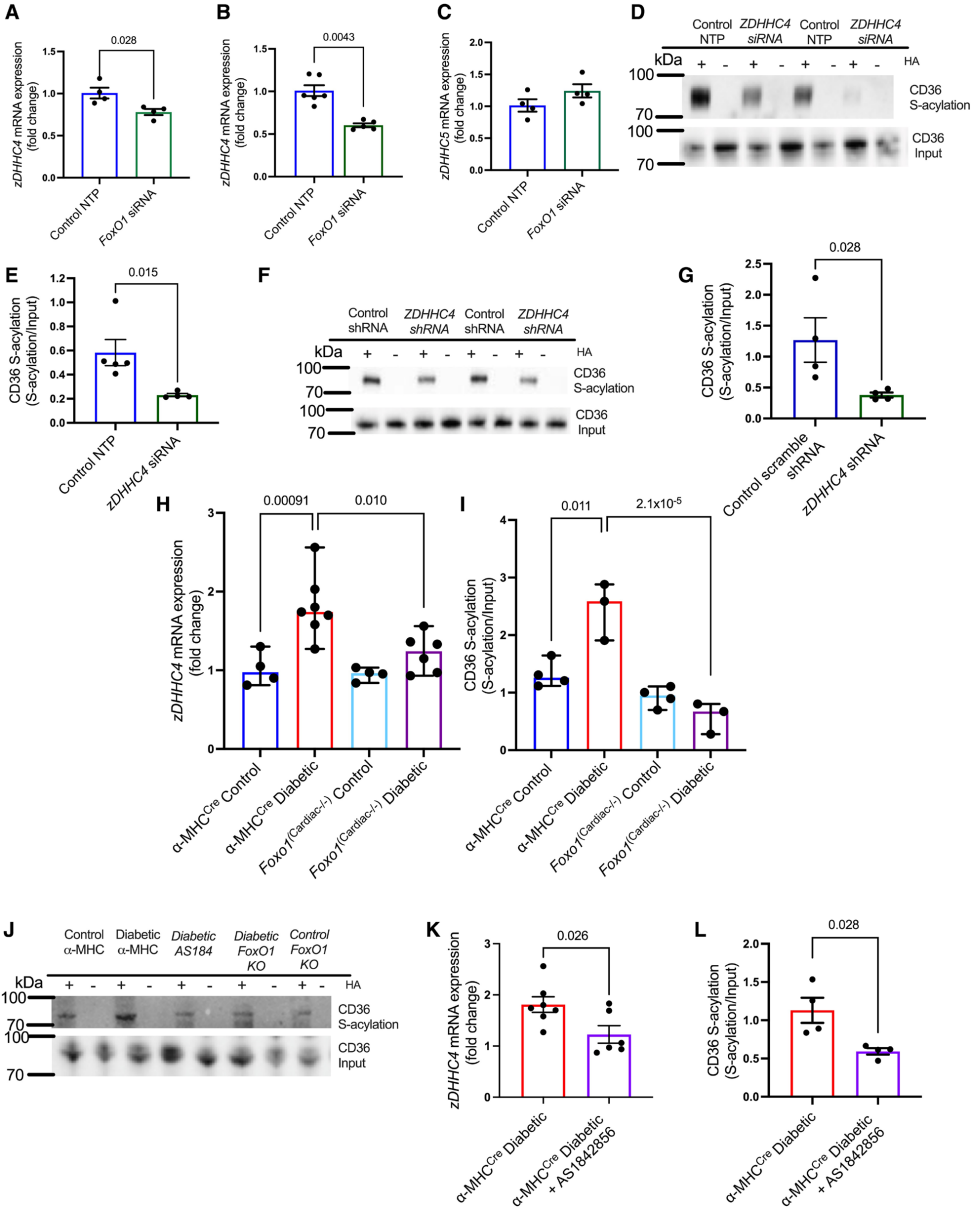
**A FoxO1 (forkhead box O1)-zDHHC4 (zinc finger DHHC-type palmitoyltransferase 4)-CD36 (fatty acid translocase/cluster of differentiation 36) S-acylation axis in diabetes.** In human induced pluripotent stem cell-derived cardiomyocyte (hiPSC-CM; **A**) and endothelial sEnd.1 (skin derived endothelial cells 1) cells (**B**), transfection with *FoxO1* siRNA (small interfering RNA) decreased the expression of *zDHHC4* mRNA relative to nontarget pool (NTP) control siRNA. **C**, In contrast, *FOXO1* siRNA did not significantly change the expression of *ZDHHC5* zinc finger DHHC-type palmitoyltransferase 5) in hiPSC-CM. **D** and **E**, In hiPSC-CM, transfection with *ZDHHC4* siRNA decreased CD36 S-acylation. In neonatal rat ventricular myocytes, lentivirus transduction with *zDHHC4* shRNA decreased CD36 S-acylation, relative to control scramble shRNA (**F** and **G**). Expression of *zDHHC4* mRNA (**H**) and CD36 S-acylation (**I** and **J**) were increased in diabetic αMHC^Cre^ (Alpha-myosin heavy chain Cre) mice compared with chow-fed αMHC^Cre^ controls but not in the diabetic *FoxO1*^Cardiac−/−^ littermates. *zDHHC4* mRNA expression (**K**) and CD36 S-acylation (**J** and **L**) were decreased in diabetic αMHC^Cre^ mice pharmacologically treated with the FoxO1 inhibitor AS1842856 compared with vehicle-treated diabetic αMHC^Cre^ mice. Data (**A**–**G** and **L**) were compared using a Mann-Whitney *U* test and (**K**) compared using an unpaired *t* test (data show the mean±SEM). Data (**H** and **I**) were compared using aligned ranks transform-based nonparametric ANOVA, with estimated post hoc pairwise contrasts through the method described by Elkin et al^45^ and the Benjamini-Hochberg false discovery rate (FDR) correction procedure (data show the median±95% CI). +HA indicates S-acylated protein (hydroxylamine); and −HA, negative control (minus hydroxylamine).

Using a variety of genetic approaches we next demonstrated a FoxO1-zDHHC4-CD36 S-acylation axis, which is upregulated in the T2D heart. In hiPSC-CM and endothelial cells, *FoxO1* siRNA decreased the expression of *zDHHC4* by 22% and 40%, respectively, compared with control nontarget pool siRNA (Figure 5A and 5B; Figure S4). However, there was no decrease in the expression of *ZDHHC5* upon FoxO1 silencing in hiPSC-CM (Figure 5C). In addition, *ZDHHC4* siRNA transfection decreased CD36 S-acylation by 60% in hiPSC-CM (Figure 5D and 5E; Figure S4). Similarly, lentivirus transduction of shRNA to *zDHHC4* decreased CD36 S-acylation by 70%, compared with scramble lentivirus shRNA in neonatal rat ventricular myocytes (Figure 5F and 5G; Figure S4).

To confirm that this FoxO1-zDHHC4-CD36 S-acylation axis was of relevance in T2D in vivo, we investigated the effects of FoxO1 genetic silencing or pharmacological inhibition in diabetic mice. Induction of diabetes in αMHC^Cre^ control mice increased *zDHHC4* expression by 78% (Figure 5H) and increased CD36 S-acylation 1.8-fold (Figure 5I and 5J). Inducing diabetes in the FoxO1-deficient littermates (*Foxo1*^Cardiac^^−/−^) prevented this response, with no significant increase in *zDHHC4* expression or CD36 S-acylation relative to controls in response to diabetes. We further confirmed this pharmacologically using AS1842856, which blocks the transcriptional activity of FoxO1.^14,36^ Pharmacologically treating diabetic αMHC^Cre^ mice with AS1842856 decreased *zDHHC4* mRNA levels by 32% (Figure 5K) and decreased CD36 S-acylation by 47% compared with vehicle-treated diabetic αMHC^Cre^ mice (Figure 5L and 5J). These changes in CD36 S-acylation following manipulation of FoxO1 were not seen for other S-acylated proteins such as CAV3 (Figure S4). Therefore, these results demonstrate a FoxO1-DHHC4-CD36 S-acylation axis, which is upregulated by diabetes.

### Decreasing CD36 S-Acylation in Diabetic Hearts Using the zDHHC Inhibitor CMA Corrects Metabolic and Contractile Dysfunction

**Figure 6.**
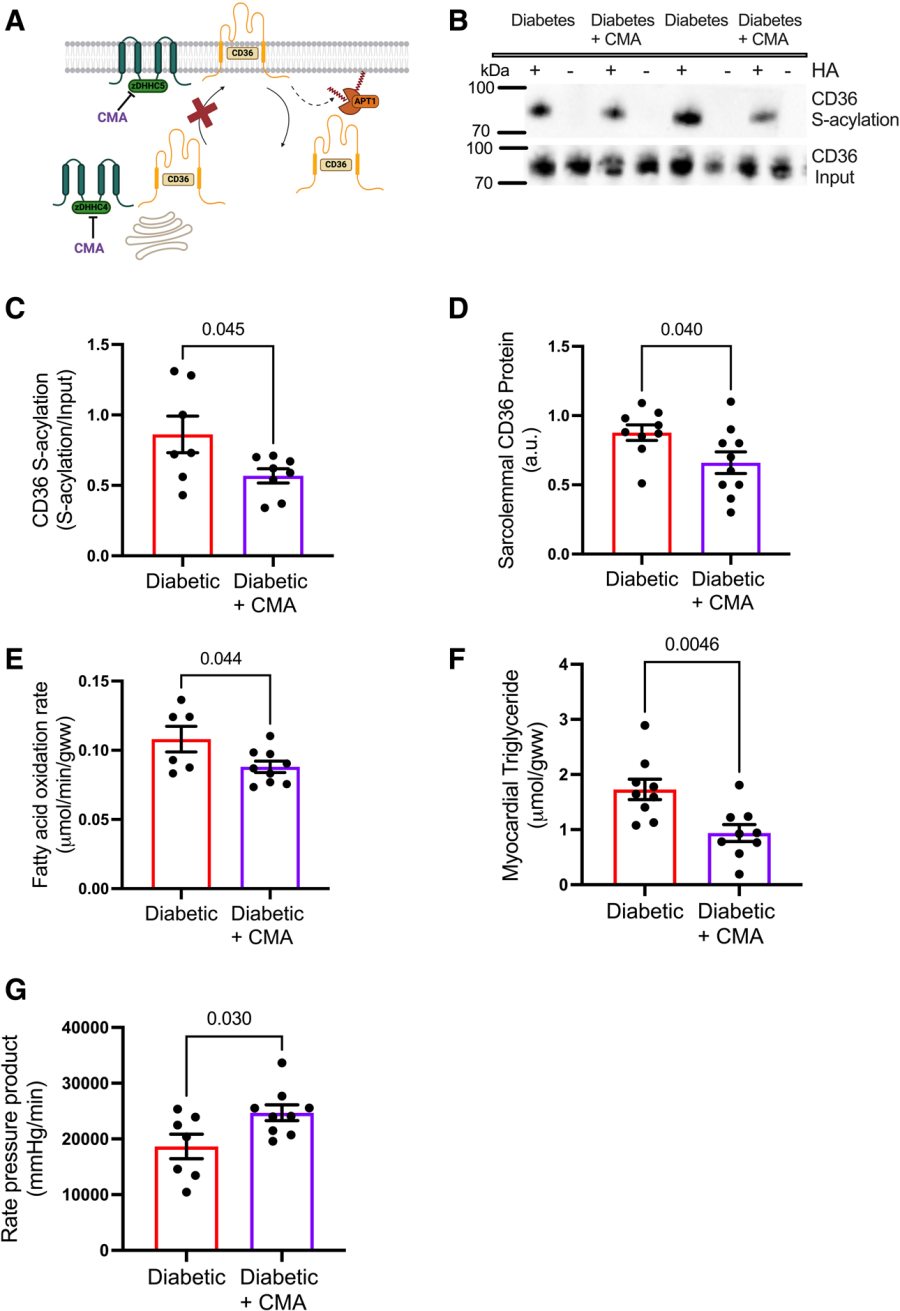
**Pharmacologically inhibiting the S-acylating zDHHC (zinc finger DHHC-type palmitoyltransferase) enzymes corrects metabolism and function in the diabetic heart.** The zDHHC inhibitor cyano-myracrylamide (CMA; **A**) decreased CD36 (fatty acid translocase/cluster of differentiation 36) S-acylation in diabetic hearts compared with untreated diabetic hearts (**B** and **C**). Sarcolemmal CD36 (**D**), fatty acid oxidation rates (**E**), and myocardial triglyceride concentrations (**F**) were decreased in diabetic hearts treated with CMA compared with untreated diabetic hearts. CMA treatment of diabetic hearts significantly improved cardiac function as assessed by rate pressure product (**G**) compared with untreated diabetic hearts. Data (**C**–**G**) were compared using a 2-tailed unpaired *t* test (data show the mean±SEM). +HA indicates S-acylated protein (hydroxylamine); and −HA, negative control (minus hydroxylamine).

To determine whether increased CD36 S-acylation contributes to cardiac metabolic and contractile dysfunction in diabetes, we infused the zDHHC inhibitor CMA into diabetic hearts (Figure 6A). In diabetic hearts, treatment with CMA resulted in a 34% decrease in CD36 S-acylation levels (Figure 6B and 6C), compared with untreated diabetic hearts. This decrease in CD36 S-acylation was associated with a 24% decrease in CD36 localization to the sarcolemma (Figure 6D). CMA treatment of diabetic hearts decreased FA oxidation rates by 21% (Figure 6E) and decreased myocardial triglycerides by 48% (Figure 6F). This direction of metabolic remodeling induced by CMA was toward that seen in control hearts. These improvements in metabolism were accompanied by a 43% increase in cardiac function, as measured by rate pressure product, which was driven by improvements in developed pressure and *dP/dt* (Figure 6G; Figure S5). Collectively, these data demonstrate that CMA treatment in diabetes decreases CD36 S-acylation and localization at the sarcolemma, which improves dysregulated lipid metabolism and enhances cardiac function.

### Pharmacologically Increasing CD36 S-Acylation in Control Hearts Recapitulates Diabetic Cardiac Metabolic Dysfunction

**Figure 7.**
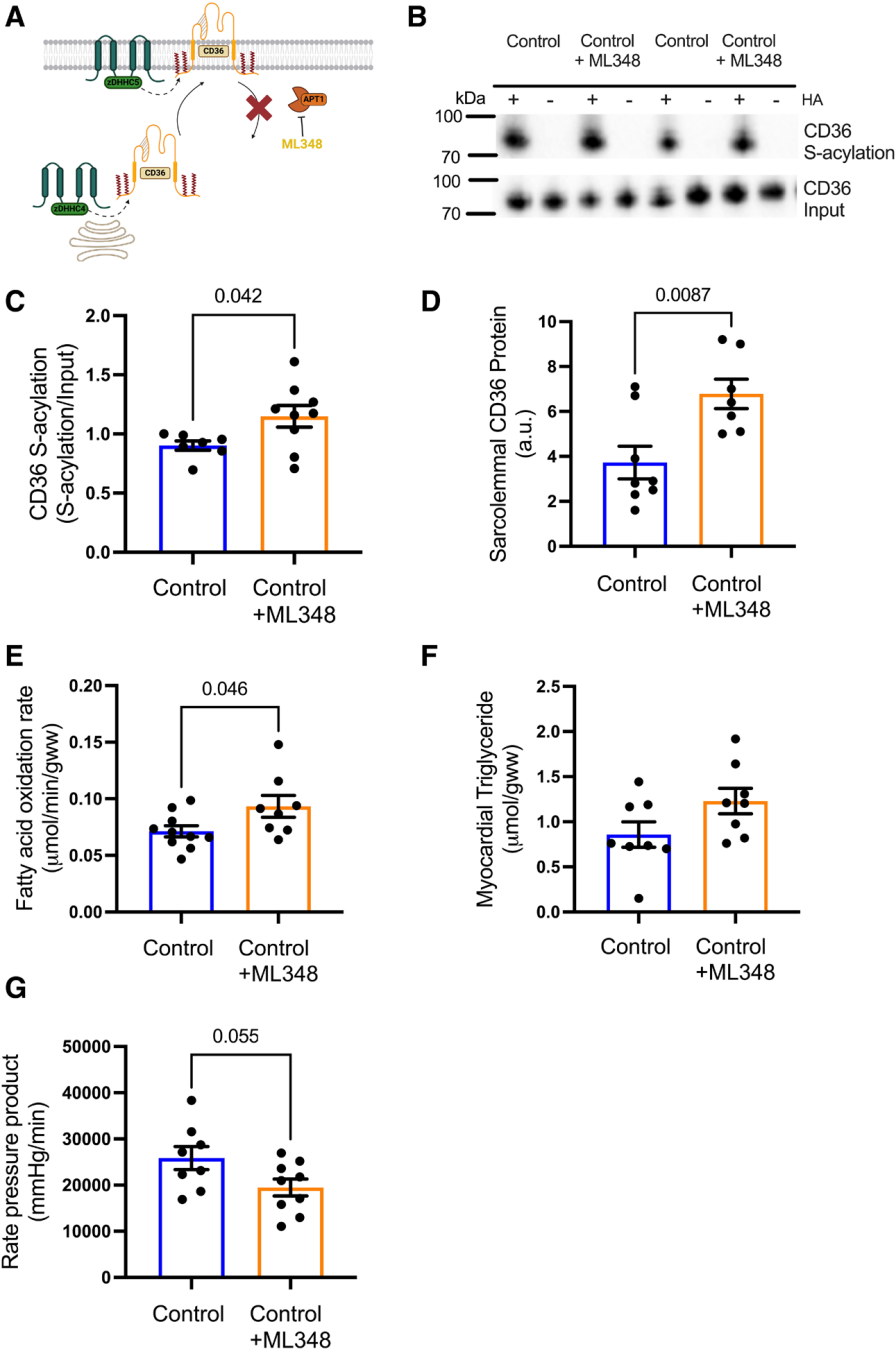
**Preventing deacylation in control hearts recapitulates the excessive fatty acid metabolism seen in diabetic hearts.** The APT1 (Acyl protein thioesterase 1) inhibitor ML348 (N-[2-chloro-5-(trifluoromethyl)phenyl]-4-(2-furanylcarbonyl)-1-piperazineacetamide) (**A**) increased CD36 (fatty acid translocase/cluster of differentiation 36) S-acylation in control hearts compared with untreated control hearts (**B** and **C**). Sarcolemmal CD36 (**D**), fatty acid oxidation rates (**E**), and myocardial triglyceride concentrations (*P*=0.08; **F**) were increased in control hearts treated with ML348 compared with untreated hearts. ML348 treatment of control hearts depressed cardiac function (*P*=0.05) as assessed by rate pressure product (**G**) compared with untreated control hearts. Data (**C**–**G**) were compared using a 2-tailed unpaired *t* test (data show the mean±SEM). +HA indicates S-acylated protein (hydroxylamine); and −HA, negative control (minus hydroxylamine).

ML348 is a well-characterized inhibitor of the deacylating enzyme APT1 (Figure 7A). We investigated whether increasing S-acylation of CD36 in a healthy heart would upregulate FA metabolism, mirroring the metabolic phenotype of the diabetic heart. In control hearts, treatment with the APT1 inhibitor ML348 increased CD36 S-acylation by 32% compared with untreated control hearts (Figure 7B and 7C). This increase in CD36 S-acylation was associated with a 50% increase in CD36 localization to the sarcolemma (Figure 7D), a 30% increase in FA oxidation rates (Figure 7E) but with no significant increase in myocardial triglyceride concentrations (Figure 7F) in hearts treated with ML348 compared with untreated controls. Finally, these metabolic changes were associated with a strong trend toward decreased cardiac function (*P*=0.055; Figure 7G), with a significant decrease in heart rate following ML348 treatment (Figure S6). Taken together, these metabolic changes induced by inhibiting deacylation in control hearts recapitulate the metabolic phenotype of the T2D heart, indicating a critical role for CD36 S-acylation in the development of cardiac metabolic dysfunction in diabetes.

## Discussion

Here, we show that CD36 S-acylation is increased in the diabetic heart due to increased expression of the S-acylating enzyme zDHHC4, upregulated by the transcription factor FoxO1. Inhibiting S-acylation decreases CD36 localization at the sarcolemma, correcting the excessive FA metabolism and improving contractile function in diabetes. Conversely, increasing CD36 acylation in healthy hearts recapitulates the increased CD36 S-acylation, membrane relocalization, and lipotoxic phenotype associated with diabetes. Thus, activation of the FoxO1-zDHHC4-CD36 axis drives metabolic dysfunction in diabetes.

### CD36 S-Acylation Is Increased in Diabetic Hearts Fueling Increased FA Metabolism

As the predominant FA transporter in the heart, CD36 sits as the metabolic gatekeeper governing FA uptake into the myocardium and determining onward metabolism into oxidative and storage pathways.^5^ Due to metabolic crosstalk between pathways as described by the Randle cycle,^46^ changes in CD36 activity regulate the utilization of other fuels and overall cardiac fuel preference. Preferential sarcolemmal relocalization of CD36 from intracellular vesicles to the plasma membrane has been identified as one of the first changes within muscle in response to a diabetogenic diet in rodents^7,47^ and humans,^48^ which, in time, is accompanied by an increase in CD36 total protein content as diabetes progresses.^49^ As such, understanding what drives this initial relocalization of CD36 to the membrane has been an active area of interest in many pathologies. In our mild model of diabetic cardiomyopathy, we have shown that a greater proportion of the CD36 pool in the heart is S-acylated, and these findings were conserved across diabetic and insulin-resistant species, including rats, mice, pigs, and humans. Although our insulin-resistant hiPSC-CMs are a model generated by replicating only some of the metabolic drivers of T2D,^18^ the magnitude of increase in CD36 S-acylation between the cardiomyocyte and in vivo models was conserved.

S-acylation not only regulates membrane trafficking of target proteins but enhances membrane affinity through enhanced hydrophobicity,^8^ which would provide a mechanism responsible for driving and maintaining a greater proportion of CD36 at the sarcolemma. Using acyl-PEG, CD36 was shown to be S-acylated on up to 4 cysteines in the heart, and others have shown that mutating these 4 cysteine residues located within the cytoplasmic tails of CD36 prevented translocation of the transporter to the membrane in response to physiological stimuli.^6^ Greater S-acylation of the CD36 pool and subsequent localization to the sarcolemma would increase the influx of FAs into the heart, fueling the increased FA oxidation and myocardial triglyceride content consistently demonstrated in both patients^2,3^ and animal models of diabetes.^50,51^

### Increased CD36 S-Acylation Is Driven by a FoxO1-zDHHC4-CD36 Axis in Diabetes

Understanding the molecular mechanism responsible for increased CD36 S-acylation is critical for identifying the drivers for metabolic dysfunction in diabetes. In adipocytes, elegant studies have shown that both zDHHC4 and zDHHC5 are required for CD36 S-acylation and trafficking to the membrane.^10^ Specifically, these 2 S-acylating enzymes work at different steps in the pathway, with zDHHC4 sending CD36 from intracellular compartments to the membrane, whereas zDHHC5 retains it at the plasma membrane upon arrival.^10^ Here, we demonstrate that *zDHHC4* is transcriptionally upregulated in diabetic hearts providing increased capacity for S-acylation, with no changes in *zDHHC5* or *APT1* expression. In addition, silencing *zDHHC4* in cardiomyocytes and endothelial cells is sufficient to decrease CD36 S-acylation.

To our knowledge, we are the first to identify that *zDHHC4* is transcriptionally regulated by the FoxO1 transcription factor. Using a combination of in silico analyses, genetic cellular studies, pharmacological approaches, and cardiomyocyte-specific knockout mice, we demonstrate that the upregulation of *zDHHC4* in diabetes is mediated by activation of the FoxO1 transcription factor. We demonstrate that FoxO1 binds to the promotor of the *zDHHC4* gene, inducing transcription of this S-acylating enzyme. Targeting FoxO1 was able to decrease *zDHHC4* and CD36 S-acylation, processes that were upregulated in the diabetic myocardium. Cardiac FoxO1 is known to be stimulated in diabetes,^52,53^ and its inhibition, through cardiac-specific FoxO1 deficient mice (*FoxO1*^Cardiac−/−^), was able to correct overall cardiac substrate metabolism.^14^ Thus, by identifying the axis between FoxO1-zDHHC4-CD36, we now provide a new mechanism to explain how FoxO1 dysregulates metabolism and contractile function in the diabetic heart. Nonetheless, as many genes involved in the regulation of metabolism, oxidative stress, and inflammation are under the transcriptional control of FoxO1,^54^ it is likely that actions independent of the FoxO1-zDHHC4-CD36 axis also contribute to how FoxO1 inhibition alleviates diabetes-related cardiac dysfunction. As an example, FoxO1 decreases glucose oxidation in the heart via promoting transcription of *Pdk4*, thereby increasing expression of PDH (pyruvate dehydrogenase) kinase 4, which phosphorylates and inactivates PDH, the rate-limiting enzyme of glucose oxidation.^36^ Moreover, pharmacological inhibition of FoxO1 fails to alleviate diastolic dysfunction in diabetic mice with a cardiac-specific deficiency of PDH,^14^ illustrating that increases in PDH activity and glucose oxidation contribute to how FoxO1 inhibition improves cardiac function in T2D.

### Pharmacological Control of S-Acylation Can Regulate Metabolism and Function of the Heart

Given that abnormal metabolism is one of the hallmarks of diabetic cardiomyopathy and has been shown to directly contribute to contractile dysfunction, strategies to correct metabolism have been of growing interest for treating the diabetic heart. We postulated that if increased S-acylation was one of the drivers for metabolic dysfunction, then strategies that decrease CD36 S-acylation should be beneficial in diabetes, and, to the contrary, strategies that increased CD36 S-acylation should be deleterious in control hearts by recapitulating the diabetic phenotype. To test the beneficial effects of decreased CD36 S-acylation on cardiac metabolism and function, we utilized the S-acylation inhibitor CMA. CMA has been shown to have greater specificity for the zDHHC enzymes with limited effects on the APTs compared with other S-acylation inhibitors such as 2-BP (2-bromopalmitate).^21^ CMA decreased CD36 S-acylation, sarcolemmal CD36, and lipid metabolism while simultaneously improving cardiac function in diabetic hearts, more closely resembling a control heart. These data demonstrate that diabetic hearts retain metabolic flexibility in response to zDHHC inhibition, and the negative effects of metabolism on function can be rapidly reversed in our mild model of diabetes. In contrast, the well-characterized APT1 inhibitor ML348 upregulated CD36 S-acylation in control hearts, and this was sufficient to recapitulate the metabolic dysfunction of diabetes. While these pharmacological agents are unable to confer specificity for the various targets of these S-acylating and deacylating enzymes, particularly as identifying the targets of these enzymes is still in its infancy, they provide proof of concept that S-acylation is critical for regulating metabolism and plays a key role in diabetic heart disease development.

In conclusion, CD36 S-acylation is increased in the diabetic heart driving sarcolemmal relocalization of CD36, resulting in increased FA metabolism. Activation of the FoxO1 transcription factor in diabetes upregulates the transcription of the S-acylating enzyme zDHHC4, driving increased CD36 S-acylation and membrane relocalization. Pharmacological approaches to modulate S-acylation regulate CD36 localization, FA metabolism, and cardiac function. This work, therefore, provides a novel FoxO1-zDHHC4-CD36 axis to explain the metabolic dysfunction in the diabetic heart, and pharmacologically targeting the regulatory enzymes involved in CD36 S-acylation is an attractive target for the treatment of the diabetic heart.

## ARTICLE INFORMATION

### Acknowledgments

The authors would like to thank Angela Russell and Leanne Hodson for their guidance. The authors are grateful to Jack Miller for statistical assistance.

Graphical images were created using BioRender (Castro Guarda M [2025] https://BioRender.com/hbzn5ke and Dennis K [2025] https://BioRender.com/3tix9ok, https://BioRender.com/x1adt9o, and https://BioRender.com/i9rqnci).

### Sources of Funding

This work was funded by grants from the British Heart Foundation (FS/17/58/33072 to L.C. Heather and FS/19/61/34900 to K.M.J.H. Dennis). This work was supported by a studentship from the Wellcome Trust (218514/Z/19/Z to R.M. Devereux), established with support from Merck Sharp and Dohme Corp and Janssen Pharmaceutica NV, and a studentship from the Biotechnology and Biological Science Research Council (BB/M011224/1 to R.D. Carter). The authors also acknowledge the Netherlands Organization for Scientific Research (NWO-ALW grant ALWOP.367 to J.J.F.P. Luiken), the Dutch CardioVascular Alliance: an initiative with the support of the Dutch Heart Foundation (grant 2020B008 RECONNEXT to D.J. Duncker), the National Institute of General Medical Sciences of the National Institutes of Health (NIH; grant R35 GM119840 to B.C. Dickinson), and the National Institute of Diabetes and Digestive and Kidney Diseases (grant F30 DK125088 to S.-A. Azizi) of the US NIH. D Aksentijevic acknowledges a Wellcome Trust Career Re-Entry Fellowship (grant 221604/Z/20/Z). J.R. Ussher is supported by an End Diabetes Award from Diabetes Canada (grant OG-3-22-5606-JU). This article is based upon work from COST Action (EU-METAHEART, CA22169), supported by COST (European Cooperation in Science and Technology).

### Disclosures

None.

### Supplemental Material

Tables S1–S3

Figures S1–S6

Major Resource Table and ARRIVE Guidelines

Uncut Gel Blots
