# FoxO1-zDHHC4-CD36 S-acylation axis drives metabolic dysfunction in diabetes

**DOI:** 10.1161/CIRCRESAHA.124.325918

**Published:** 2025-05-13

**Authors:** Kaitlyn M.J.H. Dennis, Keshav Gopal, Claudia N. Montes Aparicio, Jiashuo Aaron Zhang, Marcos Castro-Guarda, Thomas Nicol, Ríona M. Devereux, Ryan D. Carter, Saara-Anne Azizi, Tong Lan, Ujang Purnama, Carolyn A. Carr, Gul Simsek, Eleanor K. Gill, Pawel Swietach, Oana Sorop, Ilkka H.A. Heinonen, Francesco Schianchi, Joost J.F.P. Luiken, Dunja Aksentijevic, Dirk J. Duncker, Bryan C. Dickinson, Sarah De Val, John R. Ussher, William Fuller, Lisa C. Heather

**Affiliations:** 1 Department of Physiology, Anatomy and Genetics, University of Oxford, Oxford, United Kingdom; 2 Faculty of Pharmacy and Pharmaceutical Sciences, University of Alberta, Edmonton, AB, Canada; 3 Department of Chemistry, University of Oxford, Oxford, United Kingdom; 4 Department of Chemistry, University of Chicago, Chicago, Illinois, United States; 5 Division of Experimental Cardiology, Department of Cardiology, Erasmus MC, University Medical Center Rotterdam, Rotterdam, Netherlands; 6 Turku PET Centre, University of Turku and Turku University Hospital, Turku, Finland; 7 Department of Genetics and Cell Biology, Faculty of Health, Medicine and Life Sciences, Maastricht University, 6200 MD Maastricht, The Netherlands; 8 William Harvey Research Institute, Barts and the London Faculty of Medicine and Dentistry, Queen Mary University of London, London, United Kingdom; 9 School of Cardiovascular and Metabolic Health, University of Glasgow, United Kingdom

## Abstract

**Background:**

The fatty acid transporter CD36 is the gatekeeper of cardiac fatty acid metabolism. Preferential localisation of CD36 to the sarcolemma is one of the initiating cellular responses in the development of muscle insulin resistance and in the type 2 diabetic heart. Post-translational S-acylation controls protein trafficking, and in this study we hypothesised that increased CD36 S-acylation may underpin the preferential sarcolemmal localisation of CD36, driving metabolic and contractile dysfunction in diabetes.

**Methods and Results:**

Type 2 diabetes increased cardiac CD36 S-acylation, CD36 sarcolemmal localisation, fatty acid oxidation rates and triglyceride storage in the diabetic heart. CD36 S-acylation was increased in diabetic rats, *db/db* mice, diabetic pigs and insulin-resistant human iPSC-derived cardiomyocytes, demonstrating conservation between species. The enzyme responsible for S-acylating CD36, zDHHC4, was transcriptionally upregulated in the diabetic heart, and genetic silencing of zDHHC4 using siRNA or lentiviral shRNA decreased CD36 S-acylation. We identified that *zDHHC4* expression is under the regulation of the transcription factor forkhead box O1 (FoxO1), as FoxO1 binds to the promotor of zDHHC4 and induces its transcription, as assessed using ChIP-seq, ChIP-qPCR, luciferase assays and siRNA silencing. Diabetic mice with cardiomyocyte-specific FoxO1 deletion had decreased cardiac *zDHHC4* expression and decreased CD36 S-acylation, which was further confirmed using diabetic mice treated with the FoxO1 inhibitor AS1842856. Pharmacological inhibition of zDHHC enzymes in diabetic hearts decreased CD36 S-acylation, sarcolemmal CD36 content, fatty acid oxidation rates and triglyceride storage, culminating in improved cardiac function in diabetes. Conversely, inhibiting the de-acylating enzymes in control hearts increased CD36 S-acylation, sarcolemmal CD36 content and fatty acid metabolic rates in control hearts, recapitulating the metabolic phenotype seen in diabetic hearts.

**Conclusions:**

Activation of the FoxO1-zDHHC4-CD36 S-acylation axis in diabetes drives metabolic and contractile dysfunction in the type 2 diabetic heart.

## Abbreviations

CD36: fatty acid translocase/cluster of differentiation 36.
T2D: type 2 diabetes
zDHHC: zDHHC family of S-acyltransferases
FoxO: forkhead box O transcription factors
FA: fatty acid
PTM: post-translational modification
APT: acyl-protein thioesterases
IR: insulin resistance
CAV: caveolin
CMA: cyano-myracrylamide

## Introduction

Cardiovascular disease is the leading cause of mortality in type 2 diabetes (T2D) patients, due to the development of diabetic cardiomyopathy, increased incidence of myocardial infarction and accelerated progression of heart failure. There is a growing body of research demonstrating that abnormal cardiac metabolism is a key mechanism driving cardiac dysfunction in diabetes^1^. Diabetes causes a metabolic shift within the heart, increasing fatty acid oxidation and myocardial triglyceride storage, with concomitant decreases in glucose oxidation and glycolysis^2,3^. As such, T2D is a systemic metabolic disorder associated with a cardiac pathological overdependence on fatty acid utilisation^4^.

The primary step in cardiac fatty acid metabolism is uptake across the sarcolemma, and the fatty acid transporter CD36 is responsible for the majority of fatty acid uptake into the heart^5^. CD36 is an integral membrane protein that translocates from intracellular endosomes to the sarcolemma in response to stimuli, to increase fatty acid uptake and fuel onward metabolism^6^. In diabetes, CD36 is preferentially relocated to the sarcolemma mediating increased FA uptake, with increased sarcolemmal localisation identified as one of the earliest changes in the development of insulin resistance in skeletal muscle^7^. Therefore, metabolic dysfunction in diabetes is associated with abnormal CD36 trafficking, though the mechanisms driving this remain unclear.

Recently, a lipid post-translational modification (PTM), S-acylation, which regulates membrane trafficking of various proteins, has sparked interest in the field of metabolism. S-acylation involves the covalent addition of a long chain fatty acid, most commonly palmitate, to a cysteine thiol through a thioester bond^8^ (also referred to as S-palmitoylation). S-acylated proteins can undergo reversible cycles of S-acylation and de-S-acylation with timescales ranging from seconds to hours^8^. The S-acylation reaction is catalysed by zDHHC enzymes, and de-S-acylation reaction is catalysed by a family of enzymes including acyl protein thioesterases (APT) and α/β-hydrolase domain (ABHD) enzymes^8^. S-acylation of CD36 regulates its trafficking in adipocytes^9,10^.

This study sets out to investigate if S-acylation of CD36 is a driving force for metabolic and contractile dysfunction in the diabetic heart, and, critically, if pharmacologically targeting the S-acylation and de-S-acylation enzymes can reverse dysfunction in diabetes. Using a combination of rodent, pig and human tissue in combination with genetic and pharmacological *in vivo* and *ex vivo* approaches we demonstrate increased CD36 S-acylation in diabetes, which is conserved between species and is sufficient to drive metabolic and contractile dysfunction. We identify a novel FoxO1-zDHHC4-CD36 S-acylation axis, which causes relocation of CD36 to the sarcolemma, increasing fatty acid metabolism and decreasing cardiac function. Critically, we demonstrate that pharmacological inhibition of zDHHC activity directly, or indirectly via FoxO1 inhibition and decreased zDHHC4 expression, normalizes CD36 S-acylation and localisation, fatty acid metabolism and cardiac function in type 2 diabetes. Taken together, we demonstrate that activation of the FoxO1-zDHHC4-CD36 S-acylation axis drives metabolic and contractile dysfunction in the type 2 diabetic heart, which can be reversed by pharmacologically targeting the S-acylation enzymes.

## Methods

### Rat Model of Type 2 Diabetes

Type 2 diabetes (T2D) was induced as previously described, generating an early-stage model of the disease presenting with mild hyperglycemia, hyperlipidemia, and hyperinsulinemia^11,12^. Briefly, male Wistar rats (starting body weight ∼300 g; Envigo) were fed a high-fat diet (cat. no. 829197, 60% calories from fats; Special Diet Services) for 5 weeks, and on day 14 they received a single low-dose intraperitoneal (IP) injection of streptozotocin (25 mg/kg body wt). Control rats were fed a standard chow diet for 5 weeks. Experiments conformed to the Home Office Guidance on the Operation of the Animals (Scientific Procedures) Act, 1986, and were approved by a local ethics committee (University of Oxford, UK). Males have a higher prevalence of type 2 diabetes (estimated at 17.7 million more males than females worldwide)^13^, so this study focused on the effect in male animals.

### Mouse models of Type 2 Diabetes

Snap-frozen cardiac tissue from male 13-week C57BL/KsJ-lepr^*db*^/lepr^*db*^ (*db/db*) and lean control heterozygote (*db/*+) mice (Charles River, Italy) were investigated. Alpha-myosin heavy chain Cre (αMHC^Cre^) (The Jackson Laboratory) and cardiac-specific FoxO1 deficient (*Foxo1*^fl/fl^ αMHC^Cre^, hereafter referred to as *Foxo1*^Cardiac−/−^) littermates on a C57BL/6J background were generated as previously described^14^. αMHC^Cre^ mice were either fed a chow diet (αMHC^Cre^ Control), or a high fat diet (HFD, 60% kcal from lard, Research Diets D12492) for 10 weeks with a single IP injection of streptozotocin (75 mg/kg body wt) at 4 weeks (αMHC^Cre^ Diabetic). A subset of these diabetic mice received twice daily oral gavage with the FoxO1 inhibitor AS1842856 (100 mg/kg) (MedChemExpress LLC, USA) for the final 2 weeks of the dietary protocol (αMHC^Cre^ Diabetic + AS1842856). Cardiac-specific FoxO1 deficient (*Foxo1*^(Cardiac−/−)^ mice^14^ were either fed chow or underwent the same HFD/single IP injection of streptozotocin to induce diabetes as the littermate αMHC^Cre^ mice, to give models of both pharmacological and genetic FoxO1 inhibition on the same genetic and diabetes background. Neither *Foxo1*^(Cardiac−/−)^ mice or AS1842856-treated mice showed any difference in body weight or food intake when compared with their respective control^14^.

### Porcine model of Type 2 Diabetes

Frozen heart tissue (left ventricular free wall) from diabetic and control adult male Göttingen minipigs were studied^15^. Briefly, diabetes was induced in the swine by intravenous injections of streptozotocin (25mg/kg/day) over 3 days. One week later, the swine were fed a high fat and high sugar diet (25% saturated fats, 10% sucrose and 15% fructose) for 5 months, whereas the healthy control swine consumed normal pig chow^15^.

### Insulin resistant human induced pluripotent stem cell-derived cardiomyocyte (hiPSC-CM)

Human induced pluripotent stem cells (hiPSCs) (IMR90) were differentiated into cardiomyocytes and matured using our previously published protocol^16,17^. Insulin resistance was induced in the matured contracting hiPSC-CM as previously described^17^ and characterised^18^. Briefly, mature hiPSC-CM were cultured for 3 days in glucose-free insulin resistance media comprising DMEM no glucose, supplemented with 0.3 mM palmitic acid:BSA (bound 6:1), 1.7 µM insulin, 5 µg/mL vitamin B12, 0.5 mM vitamin C, 0.84 µM biotin, 1X nonessential amino acids, 0.1X Penicillin/Streptomycin and 10% horse serum. On day 4, the media was switched to insulin resistance media as described above but also containing 12 mM glucose and 3.4 µM insulin for an additional 3 days.

### Isolated Heart Perfusion

Hearts were isolated and arrested in ice-cold Krebs-Henseleit buffer, rapidly cannulated via the aorta, and then perfused in retrograde Langendorff mode according to our published protocol^12,19^. A fluid-filled PVC balloon connected to a pressure transducer was inserted into the left ventricle and inflated to give an end-diastolic pressure of 4–8 mmHg, and the left ventricle contracted against a constant afterload pressure of 100 mmHg. Left ventricular developed pressure was calculated as peak systolic minus end-diastolic pressure, and rate pressure product was calculated as the developed pressure multiplied by the heart rate. Hearts were perfused with recirculating Krebs-Henseleit buffer supplemented with 11 mM glucose, 0.4 mM palmitate (bound to BSA) and gassed with 95% O_2_ and 5% CO_2_, maintained at 37°C. For measurement of palmitate oxidation rates, this buffer was supplemented with 0.2 µCi/mL [9,10-^3^H] palmitate. Buffer aliquots were collected at 4-min intervals throughout the perfusion, and the conversion of ^3^H-palmitate into ^3^H_2_O was measured following Folch extraction, with the upper aqueous phase counted for radioactivity. For the low and high fat perfusions, the palmitate concentration of the KH buffer was decreased to 0.2 mM and increased to 1.2 mM, respectively, for 60 mins^20^. For the cyano-myracrylamide^21^ (CMA 100 µM) and ML348 (100 µM) perfusion experiments, the compounds were dissolved in DMSO and added into the perfusion buffer after cardiac function had been stable for 10 mins and recirculated for a further hour. T2D rat hearts were perfused with CMA and control rat hearts were perfused with ML348. Following perfusion, hearts were freeze-clamped on the cannula for subsequent analysis.

### Acyl resin-assisted capture (Acyl-RAC)

S-acylated proteins were purified from snap frozen tissue using acyl resin-assisted capture (Acyl-RAC)^22^. 10 mg of tissue was incubated in blocking buffer (2.5% SDS (w/v), 100 mM HEPES, 1 mM EDTA, pH 7.4) and free cysteines alkylated by addition of 100 mM N-ethylmaleimide and incubated at 40°C for 4 h. Excess maleimide was removed by acetone precipitation, protein pellets were washed with 70% acetone (v/v), dried and resolubilized in binding buffer (1% SDS (w/v), 100 mM HEPES, 1 mM EDTA, pH 7.4). S-acylated proteins were captured on thiopropyl sepharose resin in the presence of 200 mM hydroxylamine (pH 7.4) for 2.5 h at room temperature. An identical reaction in which hydroxylamine was replaced with 200 mM NaCl served as a negative control. Following capture of acylated proteins, the beads were extensively washed in binding buffer, and proteins were eluted by heating for 10 min at 60°C in SDS-PAGE loading buffer supplemented with 100 mM DTT. The input fraction (IF), negative control (-HA), and S-acylated (+HA) fractions were analysed to assess the enrichment of the S-acylated fraction, by dividing the +HA fractions by the IF.

### Acyl-PEG exchange

Acyl-PEG exchange was carried out using an established protocol^23^. Briefly, frozen tissue was incubated in blocking buffer (2.5% SDS (w/v), 100 mM HEPES, 1 mM EDTA, pH 7.4) and free cysteines alkylated by incubation with 100 mM N-ethylmaleimide at 40°C for 4 h. Excess maleimide was removed by acetone precipitation, protein pellets were washed with 70% (v/v) acetone, dried, then resolubilized in binding buffer (1% SDS (w/v), 100 mM HEPES, and 1 mM EDTA, pH 7.4). Acylated proteins were PEGylated using 2 mM 5 kDa PEG maleimide in the presence of 200 mM hydroxylamine (HA, pH 7.4) for 2 h at room temperature. An identical reaction in which HA was replaced with 200 mM NaCl served as a negative control.

### Subcellular fractionation

Separation of the sarcolemmal membrane fraction from intracellular endosomes (low-density microsomal fraction) was carried out as previously described^24,25^. Briefly, cardiac tissue was incubated in a high-salt solution (20 mM HEPES, 2 M NaCl, and 5 mM NaN_3_), followed by centrifugation, resuspension in fractionation buffer (20 mM HEPES, 250 mM sucrose, 2 mM EDTA, 1 mM MgCl_2_, 5 mM NaN_3_), and homogenization using a glass hand-held homogenizer. Differential centrifugation was used to separate the different membrane fractions.

### Molecular analyses

Cardiac triglyceride concentrations were measured using a Randox triglyceride assay kit following Folch extraction^11^. RNA was extracted from cells and tissue with a RNeasy mini kit (QIAGEN), with cDNA conversion carried out with a high-capacity RNA-to-cDNA kit (Applied Biosystems), using a SensoQuest Labcycler (Geneflow). Quantitative PCR amplification was performed with Power SYBR Green PCR Master Mix with 15 ng/well of cDNA, using a StepOnePlus Real-Time PCR System machine (Applied Biosystems). Relative gene expression was calculated using the 2^−ΔΔCt^ method, normalized to the housekeeper gene (Supplementary Table 1).

For western blotting analyses, tissue was lysed in ice cold lysis buffer and 10 μg protein was loaded onto SDS-PAGE gels and separated by electrophoresis. Primary antibodies were used (Supplementary Table 2), in combination with the relevant secondary antibodies (Abcam). Even protein loading and transfer were confirmed using ponceau S total protein staining as a housekeeping loading control, and blots were imaged using a Chemidoc XRS+ imaging system (Bio-Rad) and Image Studio Software.

### Bioinformatic analysis of the zDHHC4 promotor

Binding motifs for the FoxO1 transcription factor around the *zDHHC4* promoter were identified using the JASPAR Transcription Factors Track Settings (minimum score 300) on the UCSC browser using mouse sequence GRCm38/mm10 and rat sequence RGSC 5.0/rn5 sequence^26,27^. FoxO1 transcription factor binding data (assessed by ChIP-seq) in the adult mouse heart was previously published^28^ and deposited on GEO with accession number GSM4278011. Enriched H3K4Me3 binding (assessed by ChIP-seq) in mouse cardiomyocytes was previously published^29^ and deposited on GEO with accession number GSM5255561. Both datasets were visualized using IGV^30^.

### RNA Sequencing

RNA was extracted from hiPSC-CM using the Qiagen RNeasy Mini kit following the manufacturer’s protocol and sequenced using an Illumina stranded paired 150-bp poly-A enrichment protocol. FastQC (v 0.12.1) was used with default settings to assess read quality and check the trimming of sequencing adaptors. Trimming of Illumina adaptors and removal of low-quality reads (q < 20) was performed with trim-galore (v 0.6.10). Salmon (v 1.10.2) tools were used for transcript quantification using the GRCh38 primary assembly^31^. For differential expression analysis and normalisation of gene-level counts, the DESEQ2^32^ pipeline was used. Gene set enrichment analysis was performed using the FGSEA^33^ package, with genes ranked by the Wald test statistic from the differential expression analysis performed with DESEQ2 between control and insulin-resistant hiPSC-CMs. The transcription factor targets pathway from msigDB^34^ evaluated FOXO1 signalling. The genes determined to contribute to the enrichment (leading edge) were extracted and plotted as normalised and scaled data to visualise each pathway on a gene level. The GSVA algorithm^35^ calculated single sample level scores for the *FOXO1* targets. Data is available deposited on GEO with an at accession number GSE288708.

### ChIP-qPCR and Luciferase

The EZ chromatin immunoprecipitation (ChIP) kit (EMD Millipore) was used according to the manufacturer’s protocol. Briefly, differentiated H9c2 cardiac myocytes were transfected with *FoxO1* wild type (WT) plasmid for 24 h using Lipofectamine 2000 and treated with 1% formaldehyde to cross-link proteins to DNA following our previously published method^36^. Cells were lysed with protease inhibitors, sonicated to shear DNA into fragments, and incubated with antibody against FoxO1 or IgG (negative control) overnight. The purified DNA and input genomic DNA were analysed by real-time PCR (Supplementary Table 1).

Mouse zDHHC4 promoter (-2000 to +100) was synthesized (Biobasic Inc., Canada) and cloned in PGL3 basic vector at Sac I and HindIII sites. Differentiated H9c2 cardiac myocytes were transfected with either *FoxO1* WT (Addgene plasmid #12148), *FoxO3* WT (Addgene plasmid #8360), or *FoxO4* WT (Addgene plasmid #17549) plasmids along with *zDHHC4* promoter-luciferase construct for 24 hrs using Lipofectamine 2000 (Thermo, USA) as per manufacturer’s instructions. *FoxO1* WT and *FoxO4* WT were gifts from Domenico Accili (Addgene plasmid # 12148 and Addgene plasmid # 17549)^37^. FoxO3 WT was a gift from Michael Greenberg (Addgene plasmid # 8360)^38^. Cells were lysed in reporter lysis buffer and a luciferase assay was performed using the bright-glow luciferase assay system (Promega Corporation, USA) as per the manufacturer’s instructions. The luciferase activity was normalized to the total protein amount and presented relative to the empty vector used for overexpression.

### Cell silencing of FoxO1 and zDHHC4

FoxO1 and zDHHC4 were silenced in matured hiPSC-CM using 50 nM of the SMARTPool *FOXO1* siRNA (L-003006-00-0005) or *ZDHHC4* siRNA (L-016699-01-0005 Dharmacon) compared with non-target pool (D-001810-10-05) siRNA using DharmaFECT 1 transfection reagent, and cells were collected 48 hours post-transfection. FoxO1 was also silenced in mouse endothelial sEnd.1 cells^39^ using 50nM of the SMARTPool *Foxo1* siRNA (L-041127-00-0005) for 24 hours. zDHHC4 was also silenced in neonatal rat ventricular myocytes (NRVMs) using LVRU6GH lentiviral particles containing either scrambled control sequence (Control scramble shRNA) or *zDHHC4*-specific shRNA (*zDHHC4* shRNA) (Genecopoeia). NRVMs were isolated and cultured according to our previously published protocol^40^. Cells were transduced with viral particles at an MOI of 10 with polybrene at a final concentration of 10 μg/mL, and were harvested for acyl-RAC 4 days post transduction.

### Statistics

Results are either presented as means ± SEM or median ± a 95% confidence limit on the median, and were considered significant at *p* values <0.05, analysed (except where otherwise specified) with GraphPad Prism Version 10. Data was tested for normality in GraphPad using the Shapiro-Wilk test when *n* > 5. For comparisons between two groups, normally distributed data was analysed using a two-tailed unpaired *t-*test. If data was not normally distributed or *n* < 5, it was analysed using a non-parametric Mann-Whitney test. For data comparing more than 2 groups either a nonparametric equivalent of the one-way ANOVA, the Kruskal-Wallis test (with Dunn’s multiple comparison post hoc test) was performed in Prism; or, for two-by-two factorial designs an aligned ranks transform based nonparametric ANOVA (with post-hoc pairwise comparisons with correction using the Benjamini-Hochberg procedure) was performed in R (version 4.2.0) utilising the ‘ARTool’ package ^41–43^. The Aligned Ranks Transform is reported, in contrast to other methods of undertaking a nonparametric ANOVA such as the Scheirer Ray Hare test, to have less inflation of type 1 error and greater statistical power^44^. We have estimated post-hoc pairwise contrasts through the method described by Elkin et al ^45^, again with the false discovery rate controlled by Benjamini-Hochberg’s procedure. For these rank-based multigroup analyses, data are presented as median ± 95% confidence interval.

## Results

### Increased fatty acid metabolism in diabetes is associated with increased sarcolemmal CD36

**Figure 1:**
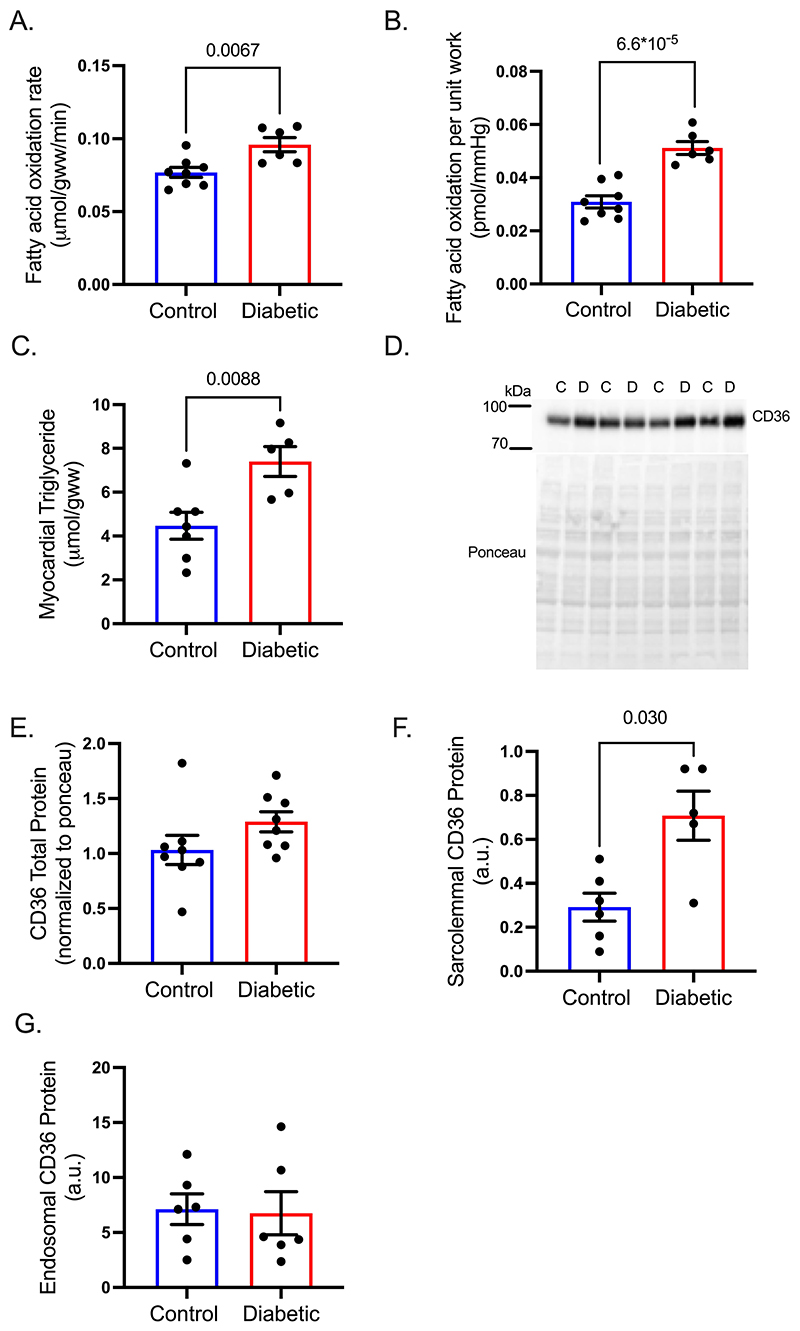
Diabetes increases cardiac fatty acid metabolism associated with increased sarcolemmal CD36. Diabetic hearts had increased fatty acid oxidation rates (**A**), fatty acid oxidation per unit work (**B**), and myocardial triglyceride concentrations (**C**), compared with control hearts. Total CD36 protein (**D-E**) was not significantly increased but sarcolemma content of CD36 was increased in diabetic hearts compared with controls (**F**), with no significant differences in endosomal CD36 content (**G**). Data (A,B,E,G) were compared using a two-tailed unpaired *t* test and (C,F) were compared using a Mann-Whitney test (data show the mean ± SEM).

Inducing type 2 diabetes (T2D) in the rat using a combination of high-fat diet and low-dose streptozotocin resulted in a mild model of T2D characterised by a 36% increase in fasting blood glucose, a 2-fold increase in non-esterified fatty acid (NEFA) concentrations and a 44% increase in fasting insulin concentrations, accompanied by increased adiposity with no significant differences in heart weight (Supplementary Table 3). Perfusing hearts in contracting Langendorff mode with ^3^H-palmitate demonstrated that fatty acid oxidation rates were increased by 25% in diabetic hearts (Figure 1A), resulting in a 66% increase in fatty acid oxidation per unit work (Figure 1B and Supplementary Figure 1), compared with control hearts. Furthermore, myocardial triglyceride concentrations were elevated by 65% in diabetes compared with controls (Figure 1C). CD36 is the predominant fatty acid transporter responsible for importing fatty acids into the cardiomyocyte. Total CD36 protein levels were not significantly different between groups, though was trending upwards in the diabetic hearts (Figure 1D-E). Subcellular fractionation of hearts demonstrated that the sarcolemmal content of CD36 was increased 2.2-fold (Figure 1F) in hearts from diabetic rats compared with controls, with no significant differences observed in CD36 within the endosomal compartment (Figure 1G). These results demonstrate that there is a preferential localisation of CD36 protein to the sarcolemma in diabetes, facilitating increased fatty acid uptake to fuel increased fatty acid oxidation and storage.

### CD36 S-acylation is increased in diabetes and conserved between species

**Figure 2:**
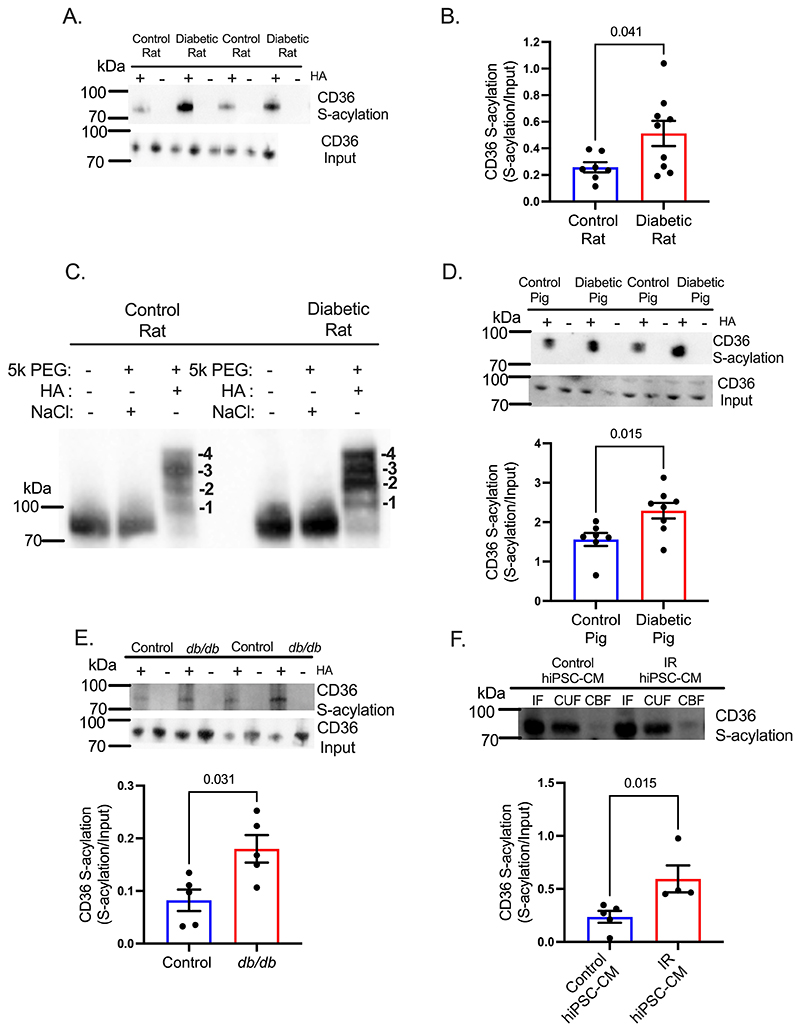
Diabetes increases cardiac CD36 S-acylation across species. Diabetic rats had increased cardiac CD36 S-acylation (**A-B**), compared with controls. CD36 is S-acylated on four cysteine residues in hearts from control and diabetic rats (**C**). Cardiac CD36 S-acylation is increased in diabetic pigs (**D**), *db/db* mice (**E**) and insulin-resistant human iPSC-CM (**F**), compared with their respective controls. +HA or CBF, S-acylated protein; -HA, negative control; IF, Input fraction; CUF, un-acylated protein. Data (B,D) were compared using a two-tailed unpaired *t* test, and (E,F) compared using a Mann-Whitney test (data show the mean ± SEM).

The post-translational modification S-acylation mediates membrane trafficking of various proteins, therefore, we investigated if increased sarcolemmal CD36 in diabetic hearts was associated with increased S-acylation. Using acyl-RAC we measured CD36 S-acylation in diabetic and control hearts, and normalized this to CD36 in the input fraction (to correct for changes in total CD36 protein). CD36 S-acylation was increased 2-fold in hearts from diabetic rats compared with controls, after normalization for CD36 content (Figure 2A-B).

Thus, a greater percentage of the CD36 pool is S-acylated in diabetic rat hearts than in controls. Furthermore, using acyl-PEG, it was determined that CD36 is S-acylated on up to four cysteine residues (Figure 2C) in control and diabetic hearts. No evidence of S-acylation was observed for the other fatty acid transport protein FABPpm or the glucose transporters GLUT1 and GLUT4 (Supplementary Figure 2), suggesting that CD36 is selectively S-acylated among the main substrate transporters in the rat heart.

To determine if the increase in CD36 S-acylation was conserved across species and between models of type 2 diabetes, we investigated diabetic pigs, mice and insulin-resistant human cardiomyocytes. CD36 S-acylation was increased by 47% in diabetic pig hearts (Figure 2D), increased by 2-fold in leptin receptor-deficient *db/db* mouse hearts (Figure 2E), and increased by 2.5-fold in insulin resistant (IR) human induced pluripotent stem cell-derived cardiomyocytes (hiPSC-CM) (Figure 2F), compared with their respective controls.

### Diabetes increases the expression of the S-acylating enzyme zDHHC4

**Figure 3:**
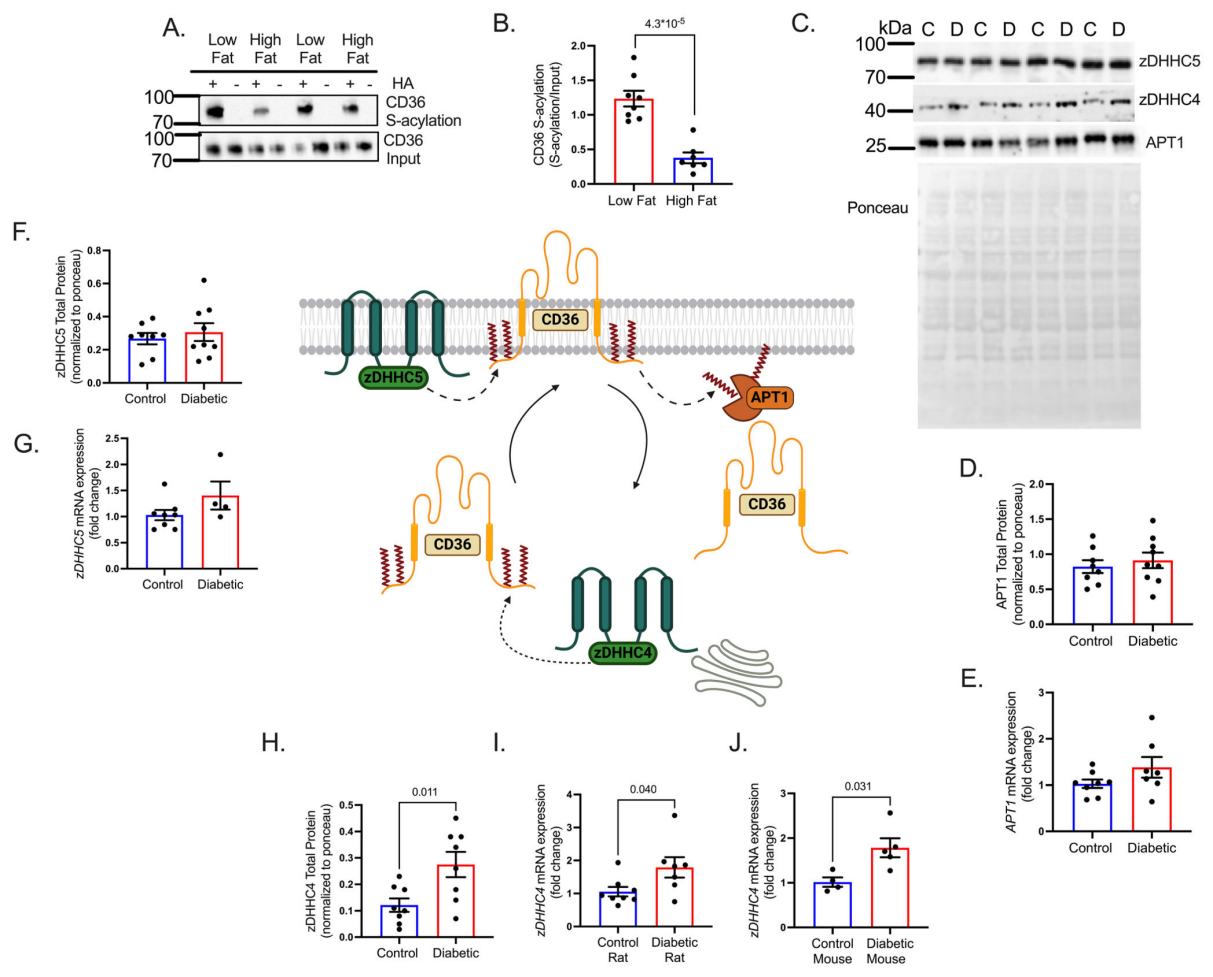
Diabetes increases expression of the S-acylating enzyme zDHHC4. Perfusion with a high fat buffer decreased CD36 S-acylation, compared with low fat perfused heart (**A-B**). Representative western blot images showing zDHHC4, zDHHC5 and APT1 protein levels in control and diabetic hearts (**C**). APT1 (**D-E**) and zDHHC5 (**F-G**) protein and mRNA expression were not significantly different between control and diabetic hearts. In contrast, zDHHC4 total protein was significantly increased in diabetic hearts, compared with controls (**H**). *zDHHC4* mRNA from diabetic rats (**I**) and diabetic mice (**J**) were significantly increased, compared with their respective controls. +HA, S-acylated protein; -HA, negative control. Data (B-F,H-I) were compared using a two-tailed unpaired *t* test, and (G,J) were compared using a Mann-Whitney test (data show the mean ± SEM).

We next investigated the mechanism driving increased CD36 S-acylation in diabetes. As palmitoyl-CoA is the main substrate for S-acylation, we investigated whether acutely increasing palmitate supply to the heart would increase CD36 S-acylation. Healthy rat hearts were perfused for 1 hour with low (0.2mM) or high (1.2mM) palmitate, causing no significant differences in cardiac function between groups (Supplementary Figure 3). Fatty acid oxidation rates were increased 3.8-fold in the high-fat perfused hearts (Supplementary Figure 3F). However, CD36 S-acylation significantly decreased in the high-fat perfused cohort compared with the low-fat group (Figure 3A-B), independently of changes in CAV3 S-acylation (Supplementary Figure 3B). Therefore, an increase in substrate availability for the zDHHCs does not directly drive enhanced CD36 S-acylation in intact beating hearts.

The main regulatory enzymes mediating CD36 S-acylation are zDHHC4 and zDHHC5^10^, and the main de-S-acylating enzyme is APT1^9^, therefore, the expression of these proteins was investigated in diabetes (Figure 3C). APT1 displayed no significant differences in total protein or mRNA expression between control and diabetes (Figure 3D-E), similarly zDHHC5, which tethers CD36 to the sarcolemma and protects it from de-S-acylation, showed no significant differences in total protein or mRNA expression between diabetic and control hearts (Figure 3F-G). However, zDHHC4, which S-acylates CD36 at the Golgi to target it for trafficking to the sarcolemma, displayed a 2.4-fold increase in protein abundance (Figure 3H) and a 73% increase in mRNA expression in diabetic hearts compared with controls (Figure 3I). These findings were recapitulated in a mouse model of T2D where *zDHHC4* mRNA was increased 82% in diabetic mouse hearts compared with controls (Figure 3J).

### The transcription factor FoxO1 drives enhanced zDHHC4 expression

**Figure 4:**
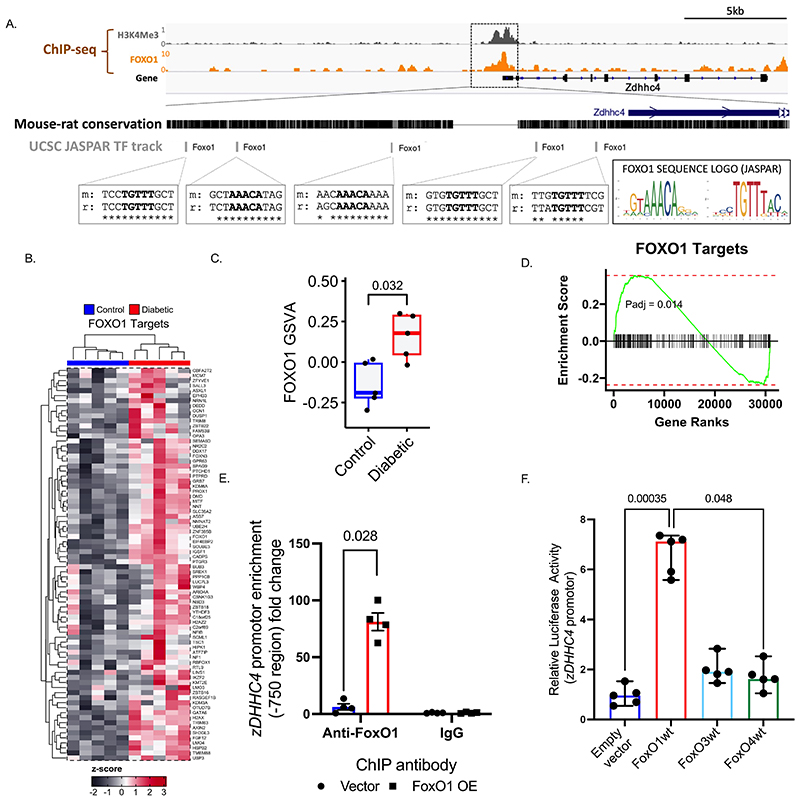
The transcription factor FoxO1 drives enhanced z*DHHC4* expression. Genomic regions around the mouse z*DHHC4* gene alongside tracks showing ChIP-seq signal for the promoter mark H3K4Me3 in mouse cardiomyocytes, and the FoxO1 transcription factor in adult mouse heart (**A**). JASPAR transcription factor track on UCSC identified five mouse-rat conserved FoxO1 binding motifs within the promoter region (core motif in bold alongside the JASPAR sequence logo for FoxO1 in both orientations) (**A**). FoxO1 target gene enrichment scores (**D**) and heat map visualisation of FoxO1 target genes demonstrate clustering between control and insulin-resistant h iPSC-CM (**B)**. Single sample enrichment scores for the FoxO1 pathway for the genes belonging to the FoxO1 pathway were increased in insulin-resistant hiPSC-CM, compared with controls (**C**). FoxO1 binds to the *zDHHC4* promotor in H9c2 cardiomyocytes measured by chromatin immunoprecipitation/quantitative (q)PCR, which is absent in the IgG control group (**E**). Transfection of a luciferase reporter construct encoding the *zDHHC4* promoter into H9c2 cardiomyocytes demonstrated increased luciferase activity in the presence of the *FoxO1* WT plasmid relative to empty vector and *FoxO4* WT plasmid (**F**). Data (C, E) were compared using a Mann-Whitney test (data show the mean ± SEM). Data (D) presents the Benjamini and Hochberg FDR corrected *p* value for the enrichment of FOXO (controlling the FDR for the 1,115 total pathways included). Data (F) were compared using a Kruskal-Wallis test with Dunn’s multiple comparison post hoc test (data show the median ± 95% CI).

To identify the transcription factor responsible for increased *zDHHC4* expression in diabetes we examined its promotor region using the JASPAR Transcription Factor Track Settings, an open-access database of transcription factors binding profiles, visualised on the UCSC browser (Figure 4A). This identified 5 binding motifs for the FoxO1 transcription factor conserved within both mouse and rat *zDHHC4* promotor sequences. Further, examination of ChIP-seq datasets in adult mouse hearts^28^, also revealed an enrichment of direct FoxO1 binding over the *zDHHC4* promotor.

Differential expression analysis was used to capture the global differences in gene expression within our insulin-resistant hiPSC-CM model. The FOXO1 targets pathway was significantly enriched in genes ranked by the global differential expression comparison (Figure 4C-D). Visualising the genes contributing to this enrichment demonstrates observable differences and robustly clusters the control and insulin-resistant groups (Figure 4B). Furthermore, calculating single sample enrichment scores for the FOXO1 pathway confirms significantly increased expression for this pathway in the insulin-resistant hiPSC-CMs for the genes belonging to the pathway (Figure 4C). Thus, FoxO1 target genes are upregulated in the insulin resistant state.

To confirm binding of FoxO1 to the *zDHHC4* promotor we performed ChIP-qPCR experiments in H9c2 cardiomyocytes overexpressing empty vector or FoxO1, using primers designed to the *zDHHC4* promotor that *in silico* analysis had identified as a potential FoxO1 binding region. This demonstrated significant 80-fold enrichment of the *zDHHC4* promotor following FoxO1 immunoprecipitation in the FoxO1 overexpressing cardiomyocytes, with negligible enrichment using IgG control antibody (Figure 4E). Additionally, we transfected H9c2 cardiac myocytes with *FoxO1* WT plasmid along with a luciferase reporter construct encoding the *zDHHC4* promoter. We found that *FoxO1* overexpression significantly increased luciferase activity 6-fold using this *zDHHC4* reporter construct, compared with the empty vector control (Figure 4F). Conversely, transfection of a plasmid encoding for other *FoxO4* WT overexpression did not increase luciferase activity via the *zDHHC4* reporter construct.

### A FoxO1-zDHHC4-CD36 S-acylation axis in diabetes

**Figure 5:**
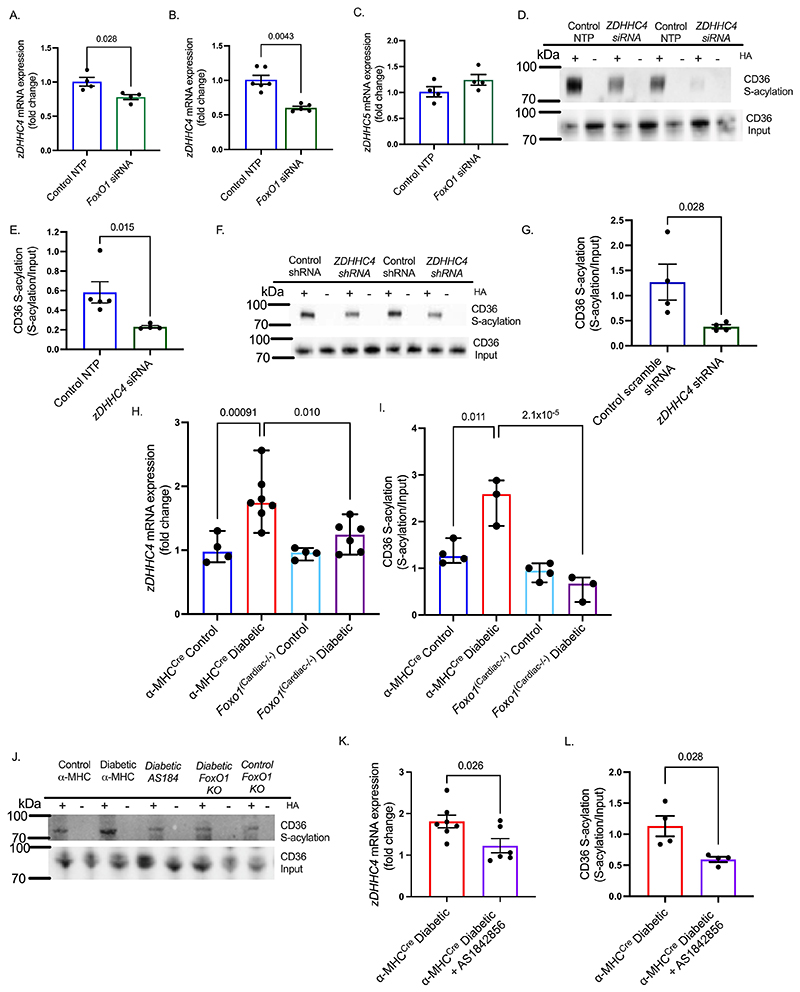
A FoxO1-zDHHC4-CD36 S-acylation axis in diabetes In hiPSC-CM (**A**) and endothelial sEnd.1 cells (**B**), transfection with *FoxO1* siRNA decreased the expression of *zDHHC4* mRNA relative to non-target pool (NTP) control siRNA. In contrast, *FOXO1* siRNA did not significantly change the expression of *ZDHHC5* in hiPSC-CM (**C**). In hiPSC-CM, transfection with *ZDHHC4* siRNA decreased CD36 S-acylation (**D-E**). In neonatal rat ventricular myocytes, lentivirus transduction with *zDHHC4* shRNA decreased CD36 S-acylation, relative to control scramble shRNA (**F-G**). Expression of *zDHHC4* mRNA (**H**) and CD36 S-acylation (**I-J**) were increased in diabetic *α*MHC^Cre^ mice compared with chow fed MHC^Cre^ controls, but not in the diabetic *FoxO1*^Cardiac-/-^ littermates. *zDHHC4* mRNA expression (**K**) and CD36 S-acylation (**J**,**L**) were decreased in diabetic *α*MHC^Cre^ mice pharmacologically treated with the FoxO1 inhibitor AS1842856, compared with vehicle-treated diabetic *α*MHC^Cre^ mice. +HA, S-acylated protein; -HA, negative control. Data (A-G, L) were compared using a Mann-Whitney test, and (K) compared using an unpaired t-test (data show the mean ± SEM). Data (H-I) were compared using aligned ranks transform based nonparametric ANOVA, with estimated post-hoc pairwise contrasts through the method described by Elkin *et al* ^45^ and the Benjamini-Hochberg FDR correction procedure (data show the median ± 95% CI).

Using a variety of genetic approaches we next demonstrated a FoxO1-zDHHC4-CD36 S-acylation axis, which is upregulated in the T2D heart. In hiPSC-CM and endothelial cells, *FoxO1* siRNA decreased the expression of *zDHHC4* by 22% and 40%, respectively, when compared with control non-target pool (NTP) siRNA (Figure 5A-B, Supplementary Figure 4). However, there was no decrease in the expression of *ZDHHC5* upon FoxO1 silencing in hiPSC-CM (Figure 5C). Additionally, *ZDHHC4* siRNA transfection decreased CD36 S-acylation by 60% in hiPSC-CM (Figure 5D-E, Supplementary Figure 4). Similarly, lentivirus transduction of shRNA to *zDHHC4* decreased CD36 S-acylation by 70% compared with scramble lentivirus shRNA in neonatal rat ventricular myocytes (Figure 5F-G, Supplementary Figure 4).

To confirm this FoxO1-zDHHC4-CD36 S-acylation axis was of relevance in T2D *in vivo*, we investigated the effects of FoxO1 genetic silencing or pharmacological inhibition in diabetic mice. Induction of diabetes in *α*MHC^Cre^ Control mice increased *zDHHC4* expression by 78% (Figure 5H) and increased CD36 S-acylation 1.8-fold (Figure 5I-J). Inducing diabetes in the FoxO1-deficient littermates (*Foxo1*^Cardiac-/-^) prevented this response, with no significant increase in *zDHHC4* expression or CD36 S-acylation relative to controls in response to diabetes. We further confirmed this pharmacologically using AS1842856, which blocks the transcriptional activity of FoxO1^14,36^. Pharmacologically treating diabetic *α*MHC^Cre^ mice with AS1842856 decreased *zDHHC4* mRNA levels by 32% (Figure 5K) and decreased CD36 S-acylation by 47% compared with vehicle-treated diabetic *α*MHC^Cre^ mice (Figure 5L,J). These changes in CD36 S-acylation following manipulation of FoxO1 were not seen for other S-acylated proteins such as CAV3 (Supplementary Figure 4). Therefore, these results demonstrate a FoxO1-DHHC4-CD36 S-acylation axis, which is upregulated by diabetes.

### Decreasing CD36 S-acylation in diabetic hearts using the zDHHC inhibitor CMA corrects metabolic and contractile dysfunction

**Figure 6:**
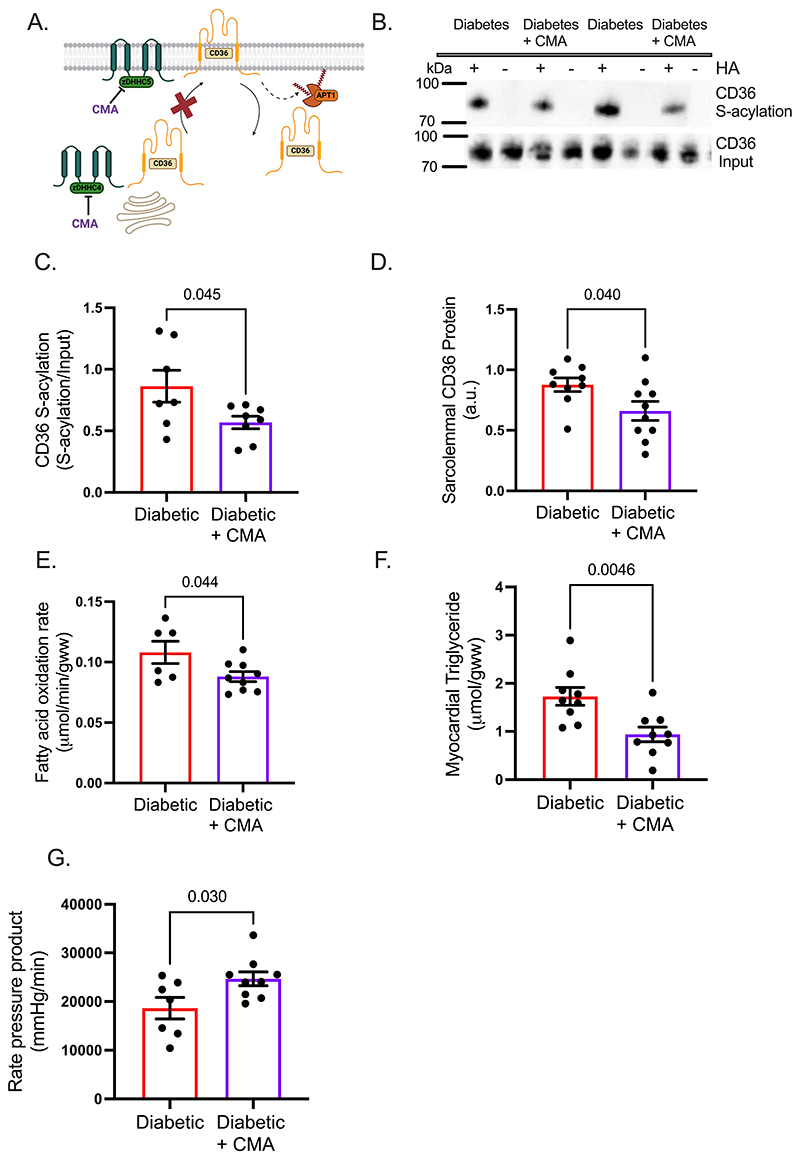
Pharmacologically inhibiting the S-acylating zDHHC enzymes corrects metabolism and function in the diabetic heart. The zDHHC inhibitor cyano-myracrylamide (CMA) (**A**) decreased CD36 S-acylation in diabetic hearts, compared with untreated diabetic hearts (**B-C**). Sarcolemmal CD36 (**D**), fatty acid oxidation rates (**E**) and myocardial triglyceide concentrations (**F**) were decreased in diabetic hearts treated with CMA, compared with untreated diabetic hearts. CMA-treatment of diabetic hearts significantly improved cardiac function as assessed by rate pressure product (**G**), compared with untreated diabetic hearts. +HA, S-acylated protein; -HA, negative control. Data (C-G) were compared using a two-tailed unpaired *t* test (data show the mean ± SEM).

To determine if increased CD36 S-acylation contributes to the cardiac metabolic and contractile dysfunction in diabetes, we infused the zDHHC inhibitor CMA into diabetic hearts (Figure 6A). In diabetic hearts, treatment with CMA resulted in a 34% decrease in CD36 S-acylation levels (Figure 6B-C), compared with untreated diabetic hearts. This decrease in CD36 S-acylation was associated with a 24% decrease in CD36 localisation to the sarcolemma (Figure 6D). CMA treatment of diabetic hearts decreased fatty acid oxidation rates by 21% (Figure 6E) and decreased myocardial triglycerides by 48% (Figure 6F). This direction of metabolic remodelling induced by CMA was towards that seen in control hearts. These improvements in metabolism were accompanied by a 43% increase in cardiac function, as measured by rate pressure product, which was driven by improvements in developed pressure and d*P*/d*t* (Figure 6G and Supplementary Figure 5). Collectively, these data demonstrate that CMA treatment in diabetes decreases CD36 S-acylation and localisation at the sarcolemma, which improves dysregulated lipid metabolism and enhances cardiac function.

### Pharmacologically increasing CD36 S-acylation in control hearts recapitulates the diabetic cardiac metabolic dysfunction

**Figure 7:**
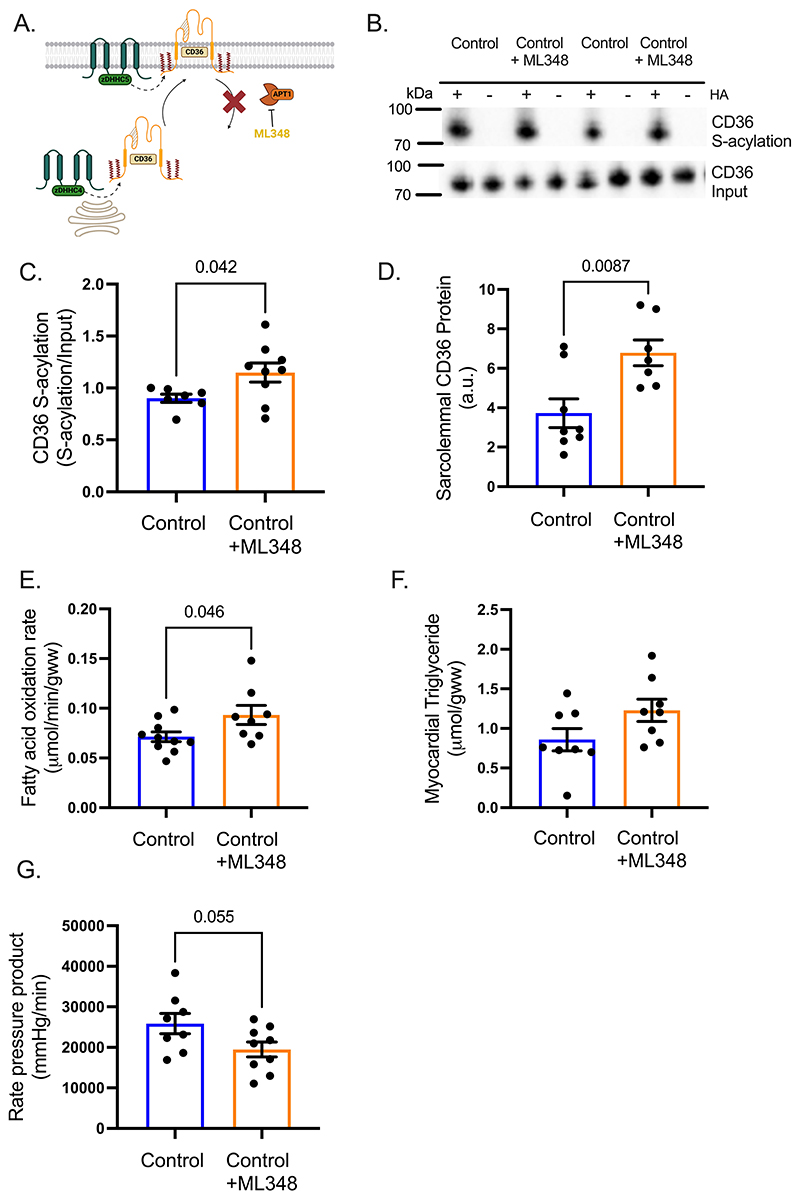
Preventing de-acylation in control hearts recapitulates the excessive fatty acid metabolism seen in diabetic hearts. The APT1 inhibitor ML348 (**A**) increased CD36 S-acylation in control hearts, compared with untreated control hearts (**B-C**). Sarcolemmal CD36 (**D**), fatty acid oxidation rates (**E**) and myocardial triglyceride concentrations (p = 0.08) (**F**) were increased in control hearts treated with ML348, compared with untreated hearts. ML348-treatment of control hearts depressed cardiac function (p = 0.05) as assessed by rate pressure product (**G**), compared with untreated control hearts. +HA, S-acylated protein; -HA, negative control. Data (C-G) were compared using a two-tailed unpaired *t* test (data show the mean ± SEM).

ML348 is a well-characterized inhibitor of the de-acylating enzyme APT1 (Figure 7A). We investigated whether increasing S-acylation of CD36 in a healthy heart would upregulate fatty acid metabolism, mirroring the metabolic phenotype of the diabetic heart. In control hearts, treatment with the APT1 inhibitor ML348 increased CD36 S-acylation by 32% compared with untreated control hearts (Figure 7B-C). This increase in CD36 S-acylation was associated with a 50% increase in CD36 localisation to the sarcolemma (Figure 7D), a 30% increase in fatty acid oxidation rates (Figure 7E) but with no significant increase in myocardial triglyceride concentrations (Figure 7F) in hearts treated with ML348 compared with untreated controls. Finally, these metabolic changes were associated with a strong trend towards decreased cardiac function (p= 0.055) (Figure 7G), with a significant decrease in heart rate following ML348 treatment (Supplementary Figure 6). Taken together, these metabolic changes induced by inhibiting de-acylation in control hearts recapitulate the metabolic phenotype of the T2D heart, indicating a critical role for CD36 S-acylation in the development of cardiac metabolic dysfunction in diabetes.

## Discussion

Here we show that CD36 S-acylation is increased in the diabetic heart due to increased expression of the S-acylating enzyme zDHHC4, upregulated by the transcription factor FoxO1. Inhibiting S-acylation decreases CD36 localisation at the sarcolemma, correcting the excessive fatty acid metabolism and improving contractile function in diabetes. Conversely, increasing CD36 acylation in healthy hearts recapitulates the increased CD36 S-acylation, membrane relocalisation and lipotoxic phenotype associated with diabetes. Thus, activation of the FoxO1-zDHHC4-CD36 axis drives metabolic dysfunction in diabetes.

### CD36 S-acylation is increased in diabetic hearts fueling increased fatty acid metabolism

As the predominant FA transporter in the heart, CD36 sits as the metabolic gatekeeper governing fatty acid uptake into the myocardium and determining onward metabolism into oxidative and storage pathways^5^. Due to metabolic crosstalk between pathways as described by the Randle cycle^46^, changes in CD36 activity regulate the utilisation of other fuels and overall cardiac fuel preference. Preferential sarcolemmal relocalisation of CD36 from intracellular vesicles to the plasma membrane has been identified as one of the first changes within muscle in response to a diabetogenic diet in rodents^7,47^ and humans^48^, which in time is accompanied by an increase in CD36 total protein content as diabetes progresses^49^. As such, understanding what drives this initial relocalisation of CD36 to the membrane has been an active area of interest in many pathologies. In our mild model of diabetic cardiomyopathy, we have shown that a greater proportion of the CD36 pool in the heart is S-acylated, and that these findings were conserved across diabetic and insulin resistant species, including rat, mouse, pig and human. Although our insulin resistant hiPSC-CM are a model generated by replicating only some of the metabolic drivers of T2D^18^, the magnitude of increase in CD36 S-acylation between the cardiomyocyte and *in vivo* models was conserved.

S-acylation not only regulates membrane trafficking of target proteins but enhances membrane affinity through enhanced hydrophobicity^8^, which would provide a mechanism responsible for driving and maintaining a greater proportion of CD36 at the sarcolemma. Using acyl-PEG, CD36 was shown to be S-acylated on up to four cysteines in the heart, and others have shown that mutating these four cysteine residues located within the cytoplasmic tails of CD36 prevented translocation of the transporter to the membrane in response to physiological stimuli^6^. Greater S-acylation of the CD36 pool and subsequent localisation to the sarcolemma would increase influx of fatty acids into the heart, fuelling the increased fatty acid oxidation and myocardial triglyceride content consistently demonstrated in both patients^2,3^ and animal models of diabetes^50,51^.

### Increased CD36 S-acylation is driven by a FoxO1-zDHHC4-CD36 axis in diabetes

Understanding the molecular mechanism responsible for increased CD36 S-acylation is critical for identifying the drivers for metabolic dysfunction in diabetes. In adipocytes, elegant studies have shown that both zDHHC4 and zDHHC5 are required for CD36 S-acylation and trafficking to the membrane^10^. Specifically, these two S-acylating enzymes work at different steps in the pathway, with zDHHC4 sending CD36 from intracellular compartments to the membrane, whereas zDHHC5 retains it at the plasma membrane upon arrival^10^. Here we demonstrate that *zDHHC4* is transcriptionally upregulated in diabetic hearts providing increased capacity for S-acylation, with no changes in *zDHHC5* or *APT1* expression. Additionally, silencing *zDHHC4* in cardiomyocytes and endothelial cells is sufficient to decrease CD36 S-acylation.

To our knowledge, we are the first to identify that *zDHHC4* is transcriptionally regulated by the FoxO1 transcription factor. Using a combination of *in silico* analyses, genetic cellular studies, pharmacological approaches and cardiomyocyte-specific knockout mice we demonstrate that the upregulation of *zDHHC4* in diabetes is mediated by activation of the FoxO1 transcription factor. We demonstrate that FoxO1 binds to the promotor of the *zDHHC4* gene, inducing transcription of this S-acylating enzyme. Targeting FoxO1 was able to decrease *zDHHC4* and CD36 S-acylation, processes that were upregulated in the diabetic myocardium. Cardiac FoxO1 is known to be stimulated in diabetes^52,53^, and its inhibition, through cardiac-specific FoxO1 deficient mice (*FoxO1*^Cardiac-/-^), was able to correct overall cardiac substrate metabolism^14^. Thus, by identifying the axis between FoxO1-zDHHC4-CD36, we now provide a new mechanism to explain how FoxO1 dysregulates metabolism and contractile function in the diabetic heart. Nonetheless, as many genes involved in the regulation of metabolism, oxidative stress, and inflammation are under the transcriptional control of FoxO1^54^, it is likely that actions independent of the FoxO1-zDHHC4-CD36 axis also contribute to how FoxO1 inhibition alleviates diabetes-related cardiac dysfunction. As an example, FoxO1 decreases glucose oxidation in the heart via promoting transcription of *Pdk4*, thereby increasing expression of pyruvate dehydrogenase (PDH) kinase 4, which phosphorylates and inactivates PDH, the rate-limiting enzyme of glucose oxidation^36^. Moreover, pharmacological inhibition of FoxO1 fails to alleviate diastolic dysfunction in diabetic mice with a cardiac-specific deficiency of PDH^14^, illustrating that increases in PDH activity and glucose oxidation contribute to how FoxO1 inhibition improves cardiac function in T2D.

### Pharmacological control of S-acylation can regulate metabolism and function of the heart

Given that abnormal metabolism is one of the hallmarks of diabetic cardiomyopathy and has been shown to directly contribute to contractile dysfunction, strategies to correct metabolism have been of growing interest for treating the diabetic heart. We postulated that if increased S-acylation was one of the drivers for metabolic dysfunction then strategies that decrease CD36 S-acylation should be beneficial in diabetes, and, to the contrary, strategies that increased CD36 S-acylation should be deleterious in control heart by recapitulating the diabetic phenotype. To test the beneficial effects of decreased CD36 S-acylation on cardiac metabolism and function, we utilized the S-acylation inhibitor CMA. CMA has been shown to have greater specificity for the zDHHC enzymes with limited effects on the APTs, compared with other S-acylation inhibitors such as 2-BP^21^. CMA decreased CD36 S-acylation, sarcolemmal CD36 and lipid metabolism, while simultaneously improving cardiac function in diabetic hearts, more closely resembling a control heart. This data demonstrates that diabetic hearts retain metabolic flexibility in response to zDHHC inhibition, and that the negative effects of metabolism on function can be rapidly reversed in our mild model of diabetes. In contrast, the well-characterized APT1 inhibitor ML348 upregulated CD36 S-acylation in control hearts, and this was sufficient to recapitulate the metabolic dysfunction of diabetes. While these pharmacological agents are unable to confer specificity for the various targets of these S-acylating and de-acylating enzymes, particularly as identifying the targets of these enzymes is still in its infancy, they provide proof of concept that S-acylation is critical for regulating metabolism, and plays a key role in diabetic heart disease development.

In conclusion, CD36 S-acylation is increased in the diabetic heart driving sarcolemmal relocalisation of CD36, resulting in increased fatty acid metabolism. Activation of the FoxO1 transcription factor in diabetes upregulates the transcription of the S-acylating enzyme zDHHC4, driving increased CD36 S-acylation and membrane relocalisation. Pharmacological approaches to modulate S-acylation regulate CD36 localisation, fatty acid metabolism and cardiac function. This work therefore provides a novel FoxO1-zDHHC4-CD36 axis to explain the metabolic dysfunction in the diabetic heart, and that pharmacologically targeting the regulatory enzymes involved in CD36 S-acylation is an attractive target for treatment of the diabetic heart.

## Supplementary Material

325918 Data Supplement

325918 Uncut Gel Blots

## Data Availability

The data that support the findings of this study are available from the corresponding author upon reasonable request.

## Novelty and Significance

### What is known?

- Metabolic dysfunction drives diabetic cardiomyopathy, however the underlying mechanism still remain elusive.
- Changes in fatty acid metabolism occur early on during disease progression.
- Understanding the mechanism responsible for increased cardiac fatty acid uptake in diabetes is critical, as this is the primary regulated step in fatty acid metabolism in the heart.
