# Supplementary material for: FoxO1-zDHHC4-CD36 S-acylation axis drives metabolic dysfunction in diabetes
Source: Circ Res. Author manuscript; Available in PMC 2025 Jun 9. (PMC12136392; doi:10.1161/CIRCRESAHA.124.325918)
Supplement: 325918 Data Supplement [file EMS204896-supplement-325918_Data_Supplement.pdf]

SUPPLEMENTAL MATERIALS

FoxO1-zDHHC4-CD36 S-acylation axis drives metabolic dysfunction in diabetes

Kaitlyn M.J.H. Dennis[1], Keshav Gopal[2], Claudia N. Montes Aparicio[1], Jiashuo Aaron Zhang[1], Marcos Castro-Guarda[1], Thomas Nicol[1], Ríona M. Devereux[1,3], Ryan D. Carter[1], Saara-Anne Azizi[4], Tong Lan[4], Ujang Purnama[1], Carolyn A. Carr[1], Gul Simsek[1], Eleanor K. Gill[1], Pawel Swietach[1], Oana Sorop[5], Ilkka H.A. Heinonen[5,6], Francesco Schianchi[7], Joost J.F.P. Luiken[7], Dunja Aksentijevic[8], Dirk J. Dunker[5], Bryan C. Dickinson[4], Sarah De Val[1], John R. Ussher[2], William Fuller[9], and Lisa C. Heather[1]*.

[1]Department of Physiology, Anatomy and Genetics, University of Oxford, Oxford, United Kingdom.
[2]Faculty of Pharmacy and Pharmaceutical Sciences, University of Alberta, Edmonton, AB, Canada.
[3]Department of Chemistry, University of Oxford, Oxford, United Kingdom.
[4]Department of Chemistry, University of Chicago, Chicago, Illinois, United States.
[5]Division of Experimental Cardiology, Department of Cardiology, Erasmus MC, University Medical Center Rotterdam, Rotterdam, Netherlands.
[6]Turku PET Centre, University of Turku and Turku University Hospital, Turku, Finland.
[7]Department of Genetics and Cell Biology, Faculty of Health, Medicine and Life Sciences, Maastricht University, 6200 MD Maastricht, The Netherlands.
[8]William Harvey Research Institute, Barts and the London Faculty of Medicine and Dentistry, Queen Mary University of London, London, United Kingdom.
[9]School of Cardiovascular and Metabolic Health, University of Glasgow, United Kingdom.

Supp 1

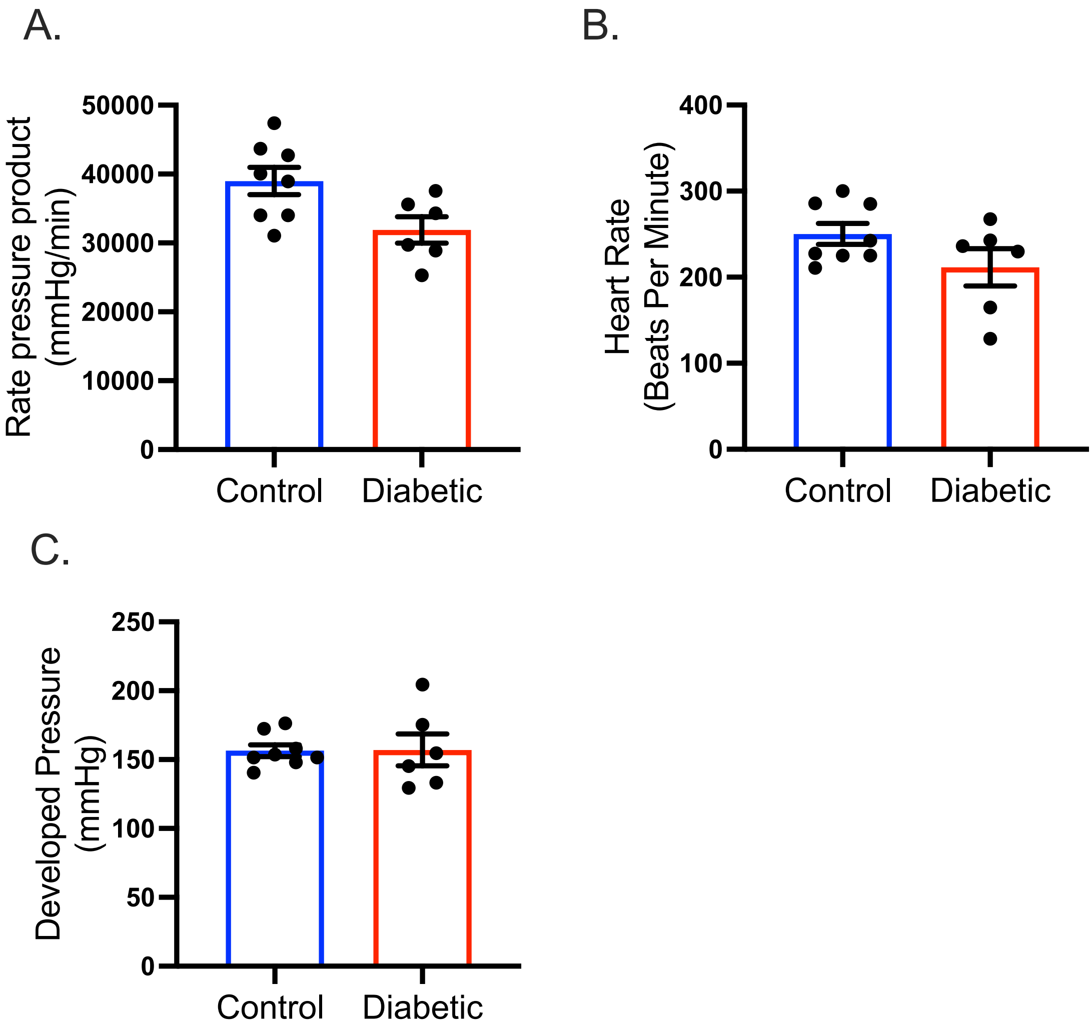

Supplementary Figure 1: Following perfusion for 1hr, rate pressure product (**A**), heart rate (**B**) and developed pressure (**C**) were not significantly different between hearts from control and diabetic rats. Data (A-C) were compared using a two-tailed unpaired *t* test (data show the mean ± SEM).

Supp 2.

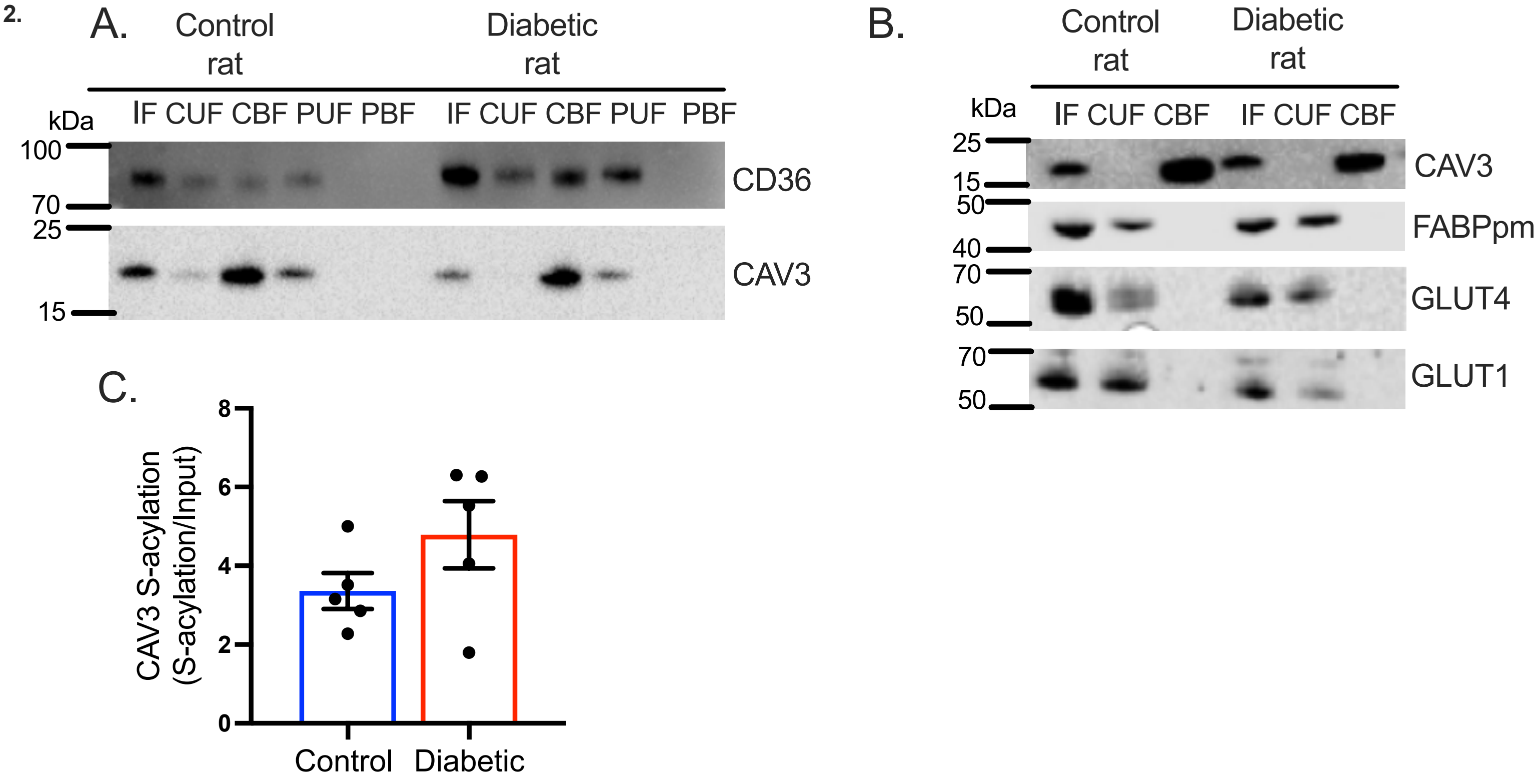

Supplementary Figure 2: The acyl-RAC assay optimization for CD36 (**A**) using CAV3 as a positive control (**A**). IF (Input fraction= total protein), CUF (cleaved proteins not caught on thiopropyl-Sepharose beads, un-S-acylated fraction of the protein), CBF (HA treated, S-acylated protein), PUF (uncleaved proteins not caught on thiopropyl-Sepharose beads, all un-S-acylated proteins), PBF (NaCl treated proteins caught on thiopropyl-Sepharose beads). No evidence for S-acylation of FABPpm, GLUT4 and GLUT1 (**B**) between control and diabetic hearts. CAV3 (**C**) S-acylation levels were not different between control and diabetes. Data (C) were compared using a Mann-Whitney test (data show the mean ± SEM).

Supp 3

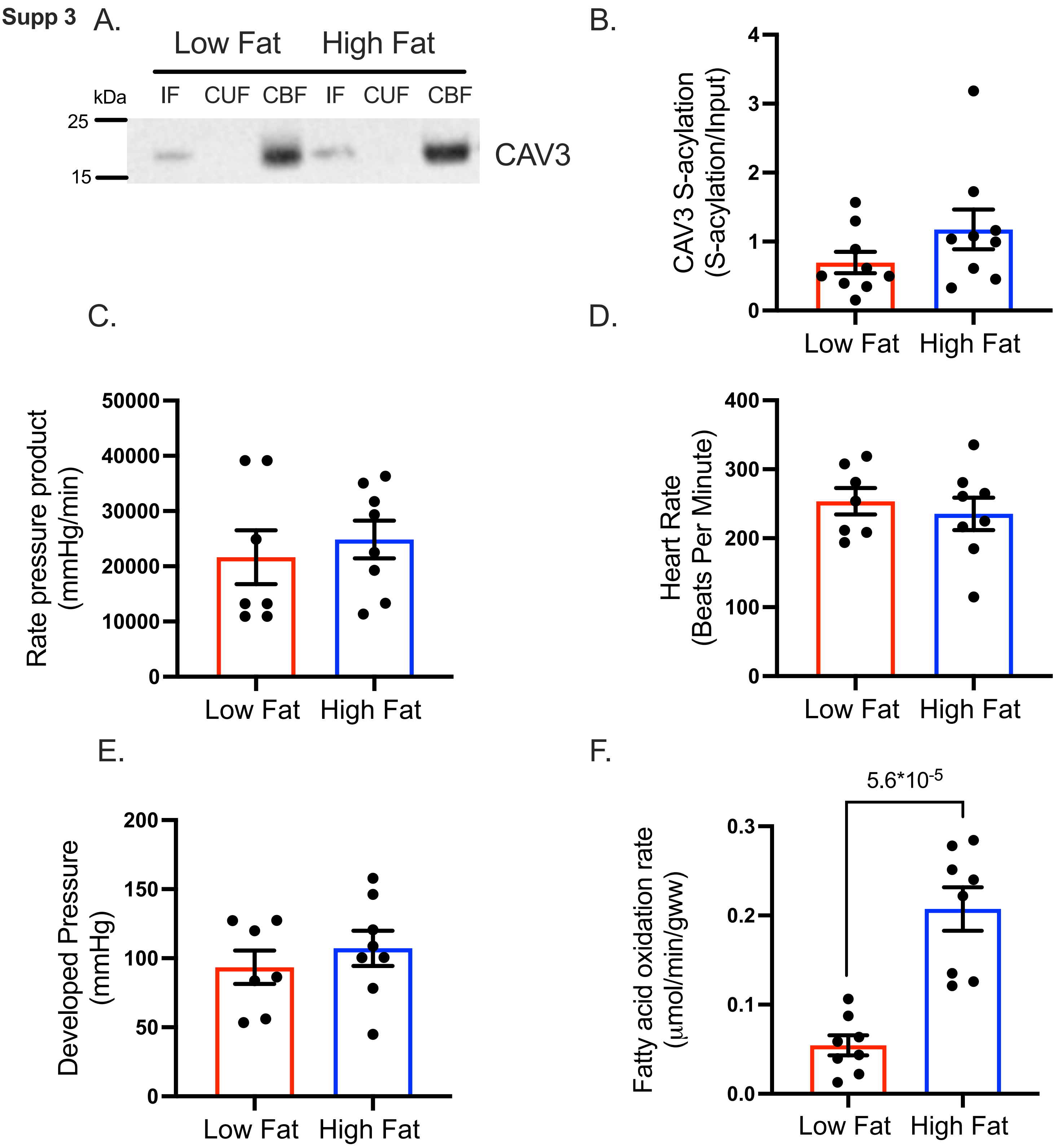

Supplementary Figure 3: CAV3 S-acylation was not significantly different between low fat and high fat perfused groups (**A-B**). Following one hour perfusion with low fat or high fat, no significant differences were observed between rate pressure product (**C**), heart rate (**D**) or developed pressure (**E**) between groups. Fatty acid oxidation rates were increased in high fat perfused hearts compared with low fat perfused hearts (**F**). Data (D-F) were compared using a two-tailed unpaired *t* test, and (B-C) compared using a Mann-Whitney test (data show the mean ± SEM).

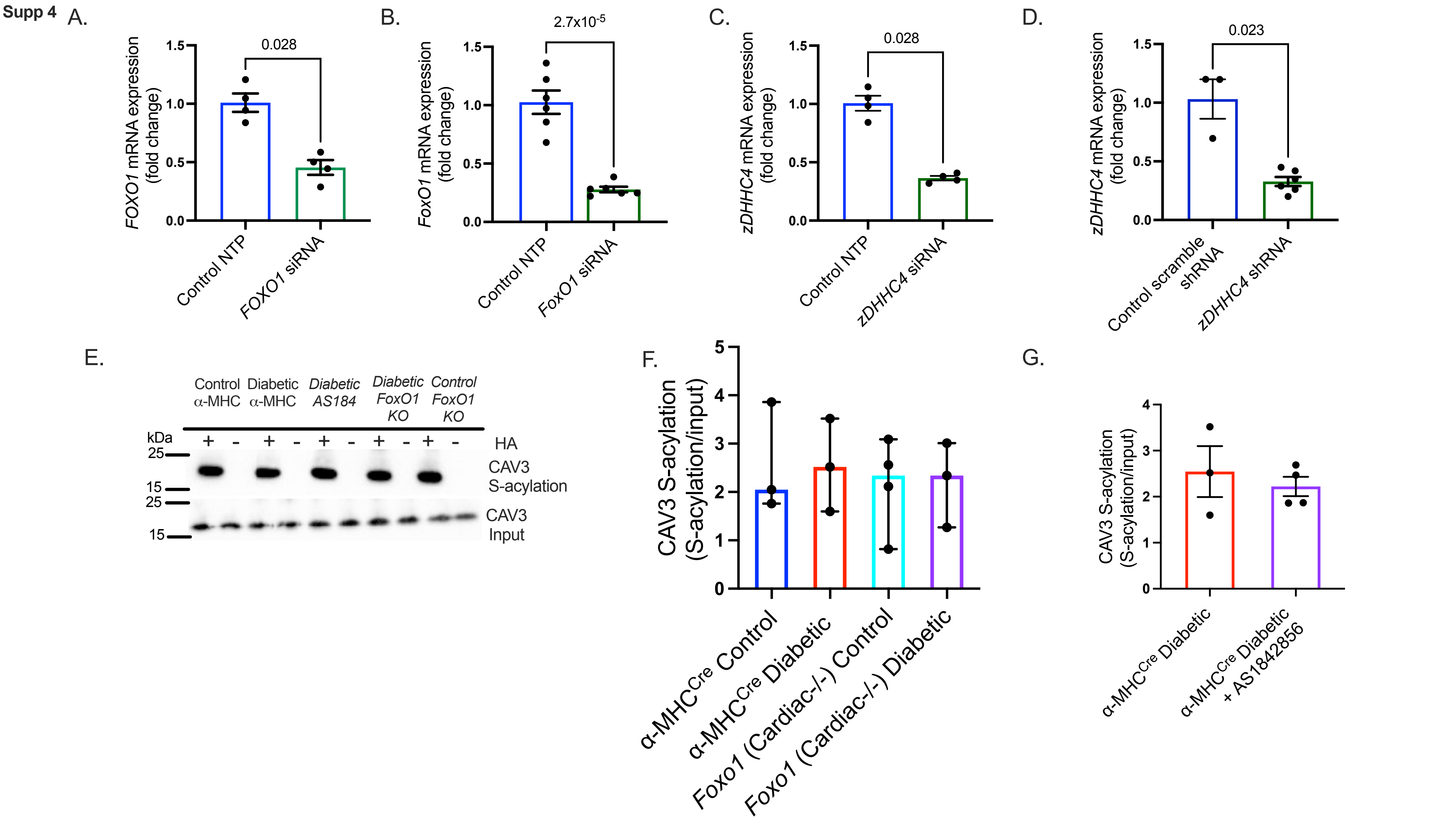

Supplementary Figure 4: FoxO1 expression in human iPSC-CM (**A**) and mouse sEND.1 (**B**) cells following silencing with FoxO1 siRNA. zDHHC4 expression in human iPSC-CM (**C**) and rat neonatal ventricular myocytes (**D**) following silencing with zDHHC4 siRNA and shRNA, respectively. CAV3 S-acylation was not significantly different following FoxO1 pharmacological and genetic manipulation (**E-G**). CAV3 S-acylation from chow fed -MHC^{Cre} control mice, -MHC^{Cre} diabetic mice, -MHC^{Cre} diabetic mice fed the FoxO1 inhibitor AS1842856 and diabetic *FoxO1*$^{fl/fl}$ αMHC^{Cre} littermates. Data (A,C-D,G) were compared using a Mann-Whitney test, and data (B) compared using a two-tailed unpaired *t* test (data show the mean ± SEM). Data expressed in (F) were compared using aligned ranks transform based nonparametric ANOVA, with estimated post-hoc pairwise contrasts through the method described by Elkin *et al* [45] and the Benjamini-Hochberg FDR correction procedure (data show the median ± 95% CI).

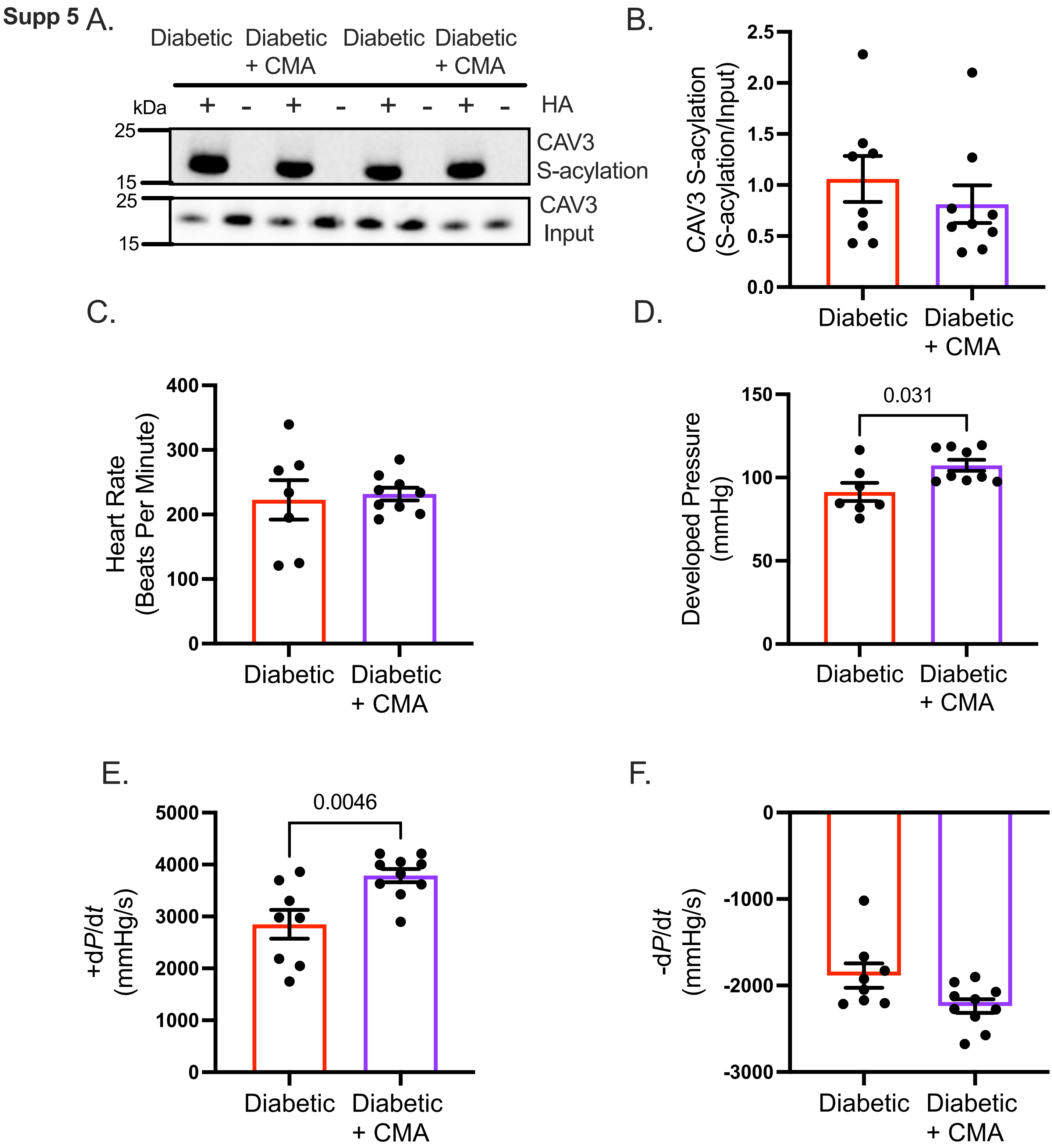

Supplementary Figure 5: CAV3 S-acylation was not significantly different between diabetic CMA and untreated diabetic hearts (**A-B**). No significant differences in heart rate were observed between CMA treated and untreated diabetic hearts (**C**), whereas developed pressure was increased in CMA treated diabetic hearts compared with untreated diabetic hearts (**D**). Positive d*P*/d*t* was significantly different in CMA treated diabetic hearts compared with untreated diabetic hearts, whereas negative d*P*/d*t* was not significantly different (**E-F**). Data (C, E) were compared using a two-tailed unpaired *t* test, and (B, D, F) compared using a Mann-Whitney test (data show the mean ± SEM).

Supp 6

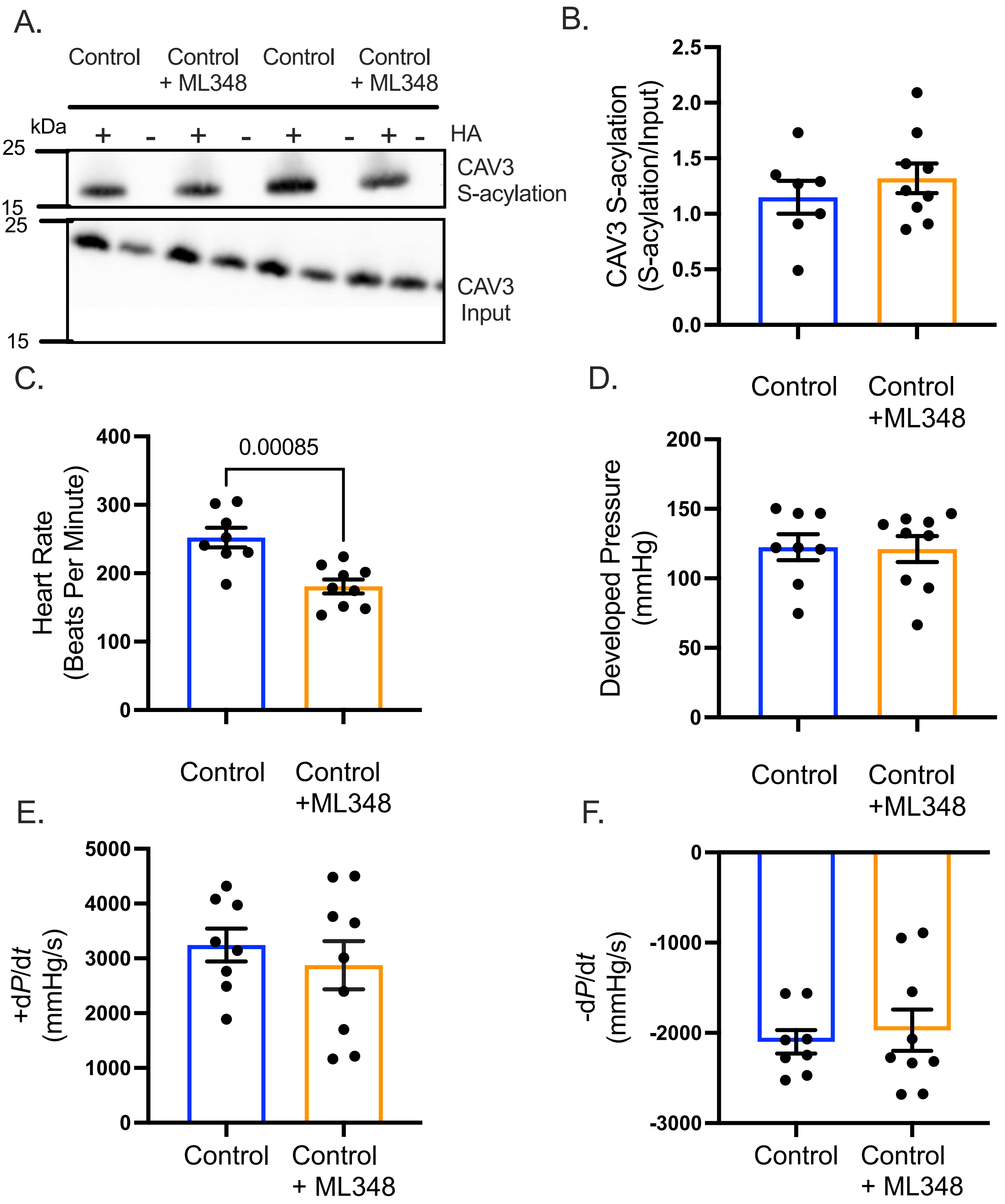

Supplementary Figure 6: CAV3 S-acylation was not significantly different between ML348 treated and untreated control rat hearts (**A-B**). Heart rate was significantly decreased in ML348 treated control hearts, compared with untreated control hearts (**C**), whereas developed pressure was not significantly different between groups (**D**). Positive d*P*/d*t* and negative d*P*/d*t* were not significantly different between groups (**E-F**). Data (B-C, E-F) were compared using a two-tailed unpaired *t* test, and (D) compared using a Mann-Whitney test (data show the mean ± SEM).

<u>Supplementary Tables.</u>

Supplementary Table 1: qPCR primer sequences

SDHA (rat)	Fp: TCCTTCCCACTGTGCATTACAA<br>Rp: CGTACAGACCAGGCACAATCTG
zDHHC4 (rat tissue)	Fp: CCTAGGGCTTACCCACCGAT<br>Rp: CTGCTGAGGGATTTCTCCCC
zDHHC4 (rat cells)	Fp: CACAGGCTTTCTCATTCAGCACC<br>Rp: CAAAGCAAAGCACAGGTAGCC
zDHHC5 (rat)	Fp: GTATCGGCCAGGTTACAGCA<br>Rp: TCTCCACGACTCAACTTGGC
APT1 (rat)	Fp: ACAGCTCATGTCAGCAGGAAAT<br>Rp: TGTGATGCTGGTGAACGTCT
APT2 (rat)	Fp: CCGGCTTCCTCATGTCAAGTA<br>Rp: CCCATCAGGTCAAACCAGGAG
zDHHC4 (mouse)	Fp: TGATTTGTGTTGTCCTGATCTGC<br>Rp: GGAGGCACTGCGGGATTAC
PPIA (mouse)	Fp: GCTGGACCAAACACAAACG<br>Rp: ATGCCTTCTTTCACCTTCCC
ZDHHC4 (human)	Fp: ATGTCTTCAGAGAGCCGTGC<br>Rp: GACAATGAAGGTGTGGTTTCTCG
ZDHHC5 (human)	Fp:CTGAGGCAGGACGGCAC<br>Rp: GGTTACTGTGTCTCACCCGC
UBC (human)	Fp: CCTGGTGCTCCGTCTTAGAG<br>Rp: TTTCCCAGCAAAGATCAACC
zDHHC4 (mouse sEnd.1 cells)	Fp: CCGGTTTGGGCCGGTTC<br>Rp: TCAGATAGCAGCTCCGCTTG
B2M (mouse sEnd.1 cells)	Fp: TTCTGGTGCTTGTCTCACTGA<br>Rp: CAGTATGTTCGGCTTCCCATTC
**ChIP-qPCR**	
zDHHC4 (-750 region)	Fp (-795): GCCAGTCGTGTATAATTTGGTGGG<br>Rp (-633): CAGCTAGGCATAGTGGCTCTTG

Supplementary Table 2: Western blotting antibodies

**Antibody**	
CD36	Monoclonal antibody MO25; a gift from Narendra Tandon, Otsuka Maryland Medicinal Laboratories, Rockville, MD
APT1	Abcam ab91606
zDHHC4	Invitrogen PA5-116794
zDHHC5	Sigma Aldrich HPA014670
CAV3	Abcam (ab2912)
GLUT1	Abcam (ab652)
GLUT4	A gift from Prof Geoff Holman, University of Bath, UK.
FABPpm	A gift from Dr Jorge Calles-Escandon, Wake Forest University School of Medicine, USA.

Supplementary Table 3: Plasma metabolites and physical characteristics from control and type 2 diabetic rats.

	Control	Diabetic	*p-value*
**Insulin (pmol/L)**	72.1 ± 25.5	110.85 ± 45.25	*0.023*
**Glucose (mmol/L)**	5.87 ± 2.40	8.00 ± 3.27	*0.022*
**NEFA (mmol/L)**	0.06 ± 0.02	0.17 ± 0.06	*0.025*
**Heart to body weight ratio (mg.g)**	3.77 ± 1.33	3.81 ± 1.56	*0.700*
**Fat pad to body weight (mg.g)**	18.3 ± 6.5	27.4 ±11.2	$7.0x10^{-4}$

N = 6 - 8 per group. NEFA; Non-esterified fatty acid. Data compared using a two-tailed unpaired *t* test (data show the mean ± SEM).

Major Resources Table

In order to allow validation and replication of experiments, all essential research materials listed in the Methods should be included in the Major Resources Table below. Authors are encouraged to use public repositories for protocols, data, code, and other materials and provide persistent identifiers and/or links to repositories when available. Authors may add or delete rows as needed.

Animals (in vivo studies)

Species	Vendor or Source	Background Strain	Sex	Persistent ID / URL
Rat	Envigo	Wistar	M	
Pig	Tissue was a gift from Dr. Dirk Duncker (Erasmus University Medical Center, Netherlands)	Gottingen minipigs	M	https://hdl.handle.net/1871.1/5c2e8cc1-b5c2-4dc3-9dda-ae9ff608c621
Mouse	The Jackson Laboratory (University of Alberta, Canada)	C57BL/6J	M	https://doi.org/10.1016/j.celrep.2021.108935

Genetically Modified Animals

	Species	Vendor or Source	Background Strain	Other Information	Persistent ID / URL
**Frozen tissue sent as a gift**	mouse	Charles River (Italy)	C57BL/KsJ-lepr^{db}/lepr^{db} (*db/db*)		
**Frozen tissue sent as a gift**	mouse	Charles River (Italy)	lean control heterozygote (*db/+*) mice		
**Frozen tissue sent as a gift**	mouse	The Jackson Laboratory (University of Alberta, Canada)	Alpha-myosin heavy chain Cre (αMHC^{Cre}) on a C57BL/6J background		
**Frozen tissue sent as a gift**	mouse	The Jackson Laboratory (University of Alberta, Canada)	*FoxO1*$^{fl/fl}$ αMHC^{Cre} on a C57BL/6J background		

Antibodies

Target antigen	Vendor or Source	Catalog #	Working concentration	Lot # (preferred but not required)	Persistent ID / URL
CD36	A gift from Narendra Tandon, Otsuka Maryland Medicinal Laboratories, Rockville, MD	NA	1/50 000		
APT1	Abcam	Ab91606	1/1000		

DOI [to be added]

DHHC4	Invitrogen	PA5-116794	1/1000		
DHHC5	Sigma	HPA014670	1/1000		
CAV3	Abcam	Ab2912	1/5000		
GLUT1	Abcam	Ab652	1/1000		
GLUT4	A gift from Prof Geoff Holman, University of Bath, UK	N/A	1/1500		
FABPpm	A gift from Dr Jorge Calles-Escandon, Wake Forest University School of Medicine, USA	N/A	1/1000		
Goat anti Rabbit	Abcam	ab6721	1/1000		
Goat anti Mouse	Abcam	ab205719	1/1000		

DNA/cDNA Clones

Clone Name	Sequence	Source / Repository	Persistent ID / URL
FoxO1 wildtype plasmid	FLAG-*FOXO1*-pCMV5	Addgene plasmid #12148	https://www.addgene.org/12148/
Mouse zDHHC4 promoter (-2000 to +100)	zDHHC4 promoter-PGL3 basic	Bio Basic Inc., ON, Canada	https://www.biobasic.com/
FoxO3 WT plasmid	FLAG-*FOXO3*-pECE	Addgene plasmid #8360	https://www.addgene.org/8360/
FoxO4 WT plasmid	FLAG-*FOXO4*-pBR322	Addgene plasmid #17549	https://www.addgene.org/17549/

Cultured Cells

Name	Vendor or Source	Sex (F, M, or unknown)	Persistent ID / URL
IMR90	Provided by Imperial College London as part of the BHF Centre of Regenerative Medicine	F	
NRVM	Gift from Pawel Swietach, University of Oxford	U	
sEND.1	Gift from Thomas Nicol, University of Oxford	U	
H9C2	ECACC (catalogue number 88092904)	U	

Data & Code Availability

DOI [to be added]

Description	Source / Repository	Persistent ID / URL
Binding motifs for the FoxO1 transcription factor around zDHHC4 promoter	Done on UCSC browser using mouse sequence GRCm38/mm10 and rat sequence RGSC 5.0/rn5 sequence	
FoxO1 transcription factor binding data in adult mouse heart (previously published)	GEO (GSM4278011)	
Enriched H3K4Me3 binding in mouse cardiomyocytes (previously published)	GEO (GSM5255561)	
RNA sequencing data of hiPSC-CMs control and insulin resistant	GEO (GSE288708)	

Other

Description	Source / Repository	Persistent ID / URL
Acyl-RAC CAPTUREome Thiol Resin	Badrilla	https://badrilla.com/product/captureome-thiol-resin/
Acyl-RAC Capture Resins (dry powder)	Nanocs	https://www.nanocs.net/Acyl-rac-capture-resin-P2.htm
Hydroxylamine Sulfate (HA)	Thermo Scientific Chemicals	https://www.thermofisher.com/order/catalog/product/198530010
^{3}H-Palmitic Acid	Perkin Elmer	https://content.perkinelmer.com/

DOI [to be added]

ARRIVE GUIDELINES

The ARRIVE guidelines (https://arriveguidelines.org/) are a checklist of recommendations to improve the reporting of research involving animals. Key elements of the study design should be included below to better enable readers to scrutinize the research adequately, evaluate its methodological rigor, and reproduce the methods or findings.

Study Design

Groups	Sex	Age	Number (prior to experiment)	Number (after termination)	Littermates (Yes/No)	Other description
Control rats	M	Adults	50	50		
Type-2 diabetes rats	M	Adults	34	34		
Control Pig	M	Adults	7	7		Frozen tissue as a gift
Diabetic Pig	M	Adults	8	8		Frozen tissue as a gift
lean control heterozygote (*db/+*) mice	M		4	4		Frozen tissue as a gift
C57BL/KsJ-lepr^{db}/lepr^{db} (*db/db*)	M		5	5		Frozen tissue as a gift
αMHC^{Cre} Control	M		3	3		Frozen tissue as a gift
αMHC^{Cre} Diabetic	M		3	3		Frozen tissue as a gift
FoxO1 $^{(Cardiac-/-)}$ Control	M		3	3		Frozen tissue as a gift
FoxO1 $^{(Cardiac-/-)}$ Diabetic	M		3	3		Frozen tissue as a gift
αMHC^{Cre} Diabetic + AS1842856	M		4	4		Frozen tissue as a gift

Sample Size: *Please explain how the sample size was decided Please provide details of any a prior sample size calculation, if done.*

Group sizes were determined by power calculations.

When we compared differences between control and treated rats ($p<0.05$ with a power of 80%), perfused hearts from wildtype rats have a fatty acid oxidation rate of 0.1 ± 0.01 µmol/gww/min. A minimum of 6 rats per group would be needed in unpaired studies to assess a 30% change in fatty acid oxidation.

Inclusion Criteria

Animals were included in the study if they successfully completed the control or High Fat Diet (HFD). We had no mortality with this protocol.

DOI [to be added]

Exclusion Criteria

Animals were excluded if Rate Pressure Product was below 10,000 mmHg/min during Langendorff Perfusions. Cardiac tissue samples used in western blotting were excluded if quantification of a protein band was not possible due to an air bubble on the membrane following transfer. Acyl-resin assisted capture western blot samples were excluded if there was low binding to the thiopropyl resin in the HA treated samples. Western blot samples were also excluded if they were identified as outliers on the Grubb's test on prism.

Randomization

All Pig and mouse tissue was a gift.

<u>Rats:</u>

Type-2 diabetes: animals were randomised by cage into control and diabetic groups, with animals within one cage typically receiving the same treatment. Feeding of the high fat diet needs to be done by cage and animals are normally allocated to STZ treatment by cage, to ensure appropriate monitoring.

Ex-vivo perfusions: Where animals were killed sequentially through the day, care was taken to ensure that animals from different groups are randomised through the day.

When studying the effect of an ex vivo therapeutic intervention, tissue is randomly assigned to treatment.

Blinding

Pig and mouse tissue were a gift.

<u>Rats:</u>

It is generally not possible to be blinded to the genotype of the animal or to administration of substances during experiments, as these details are on the cage card and necessary for appropriate monitoring. Functional cardiac data was analysed using an automated LabChart 8 computer software, run on all samples.

DOI [to be added]
