# Supplementary material for: FoxO1-zDHHC4-CD36 S-acylation axis drives metabolic dysfunction in diabetes
Source: Circ Res. Author manuscript; Available in PMC 2025 Jun 9. (PMC12136392; doi:10.1161/CIRCRESAHA.124.325918)
Supplement: 325918 Uncut Gel Blots [file EMS204896-supplement-325918_Uncut_Gel_Blots.pdf]

Gel Images

FoxO1-zDHHC4 axis increases CD36 S-acylation driving metabolic dysfunction in the type 2 diabetic heart

Kaitlyn M.J.H. Dennis[1], Keshav Gopal[2], Claudia N. Montes Aparicio[1], Jiashuo Aaron Zhang[1], Marcos Castro-Guarda[1], Thomas Nicol[1], Ríona M. Devereux[1,3], Ryan D. Carter[1], Saara-Anne Azizi[4], Tong Lan[4], Ujang Purnama[1], Carolyn A. Carr[1], Gul Simsek[1], Eleanor K. Gill[1], Pawel Swietach[1], Oana Sorop[5], Ilkka H.A. Heinonen[5,6], Francesco Schianchi[7], Joost J.F.P. Luiken[7], Dunja Aksentijevic[8], Dirk J. Dunker[5], Bryan C. Dickinson[4], Sarah De Val[1], John R. Ussher[2], William Fuller[9], and Lisa C. Heather[1]*.

[1]Department of Physiology, Anatomy and Genetics, University of Oxford, Oxford, United Kingdom.

[2]Faculty of Pharmacy and Pharmaceutical Sciences, University of Alberta, Edmonton, AB, Canada.

[3]Department of Chemistry, University of Oxford, Oxford, United Kingdom.

[4]Department of Chemistry, University of Chicago, Chicago, Illinois, United States.

[5]Division of Experimental Cardiology, Department of Cardiology, Erasmus MC, University Medical Center Rotterdam, Rotterdam, Netherlands.

[6]Turku PET Centre, University of Turku and Turku University Hospital, Turku, Finland.

[7]Department of Genetics and Cell Biology, Faculty of Health, Medicine and Life Sciences, Maastricht University, 6200 MD Maastricht, The Netherlands.

[8]William Harvey Research Institute, Barts and the London Faculty of Medicine and Dentistry, Queen Mary University of London, London, United Kingdom.

[9]School of Cardiovascular and Metabolic Health, University of Glasgow, United Kingdom.

Figure 1 D: Total CD36

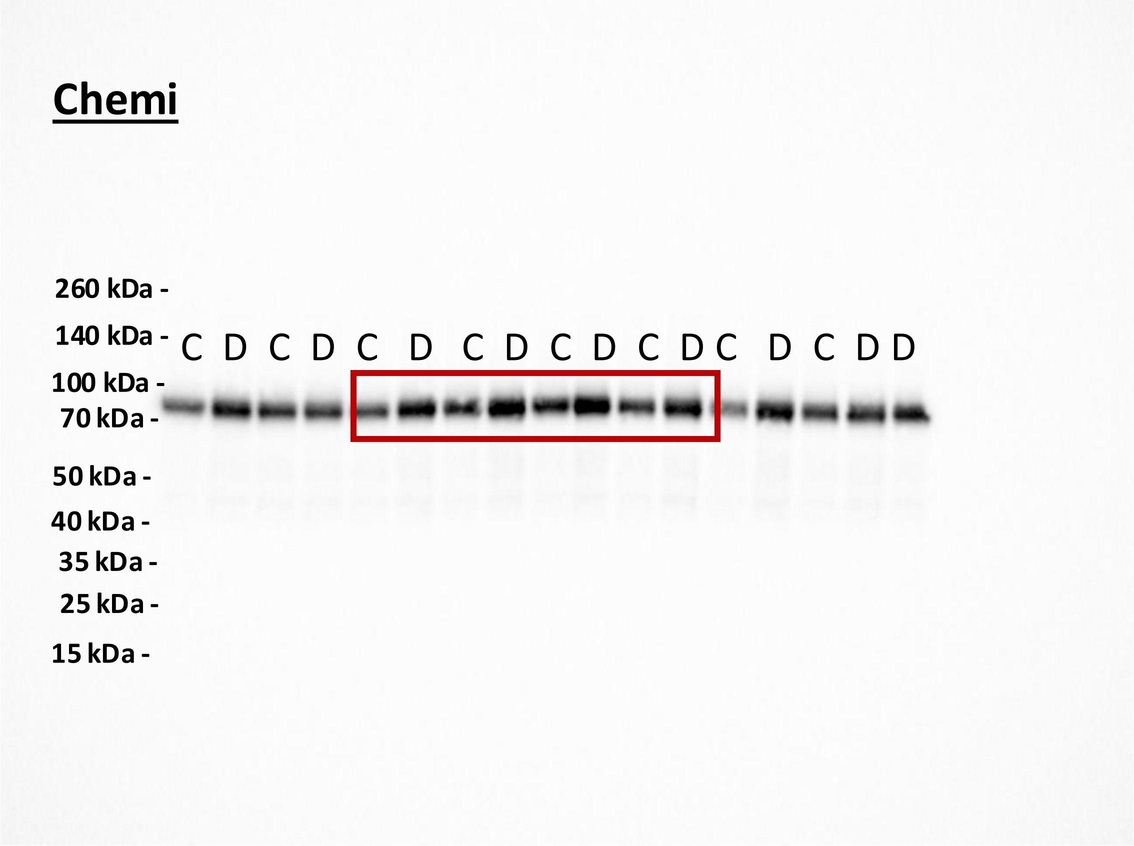

Marker

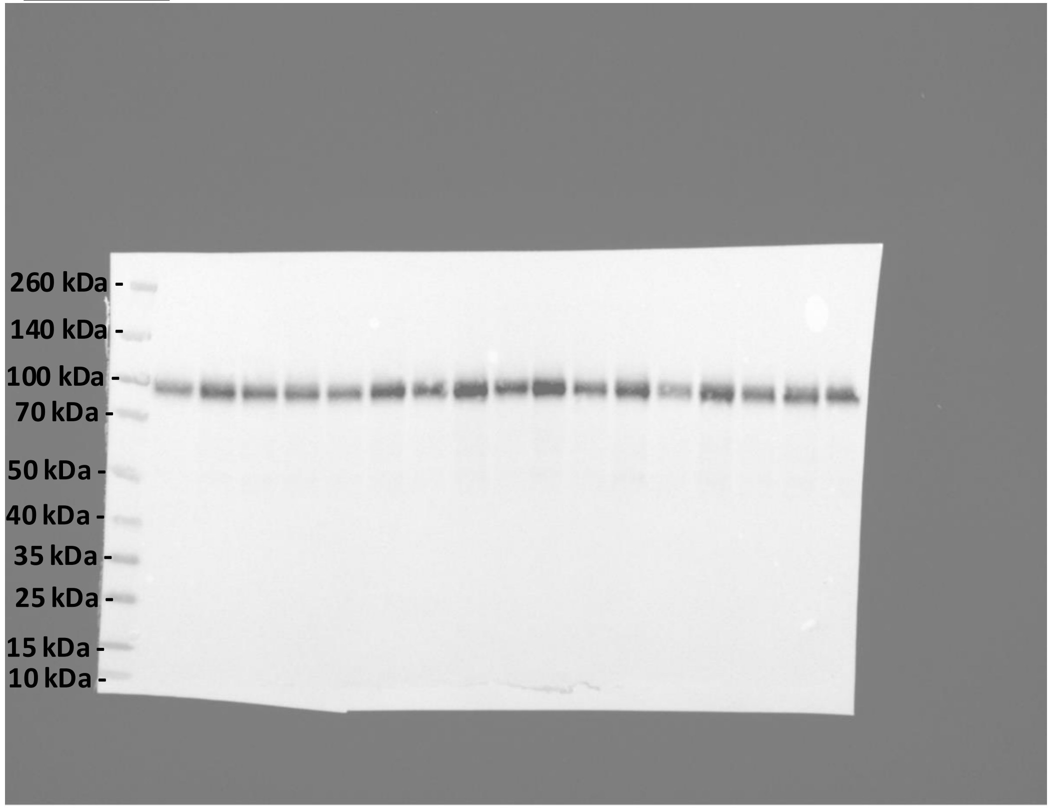

Ponceau image of membrane

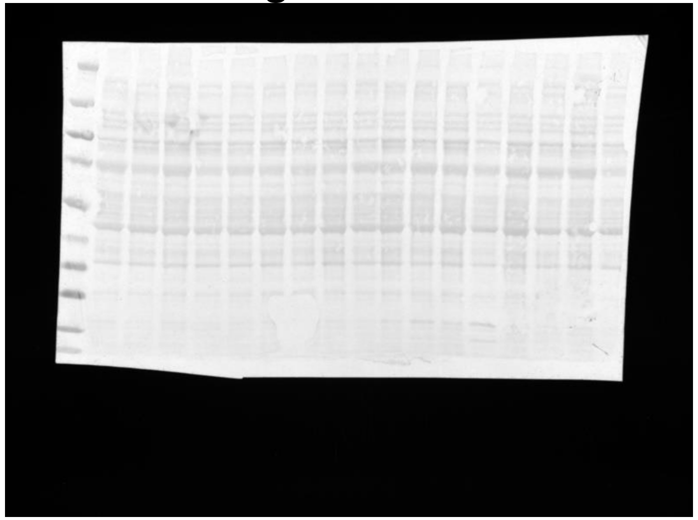

Uncropped gel and membrane – only one band for CD36 at 88kDa

Figure 3:

zDHHC4 (44 kDa), APT1 (25 kDa) and zDHHC5 (78kDa) all cut from the same membrane – same Ponceau housekeeper

Chemi overexposed to show outline of membranes

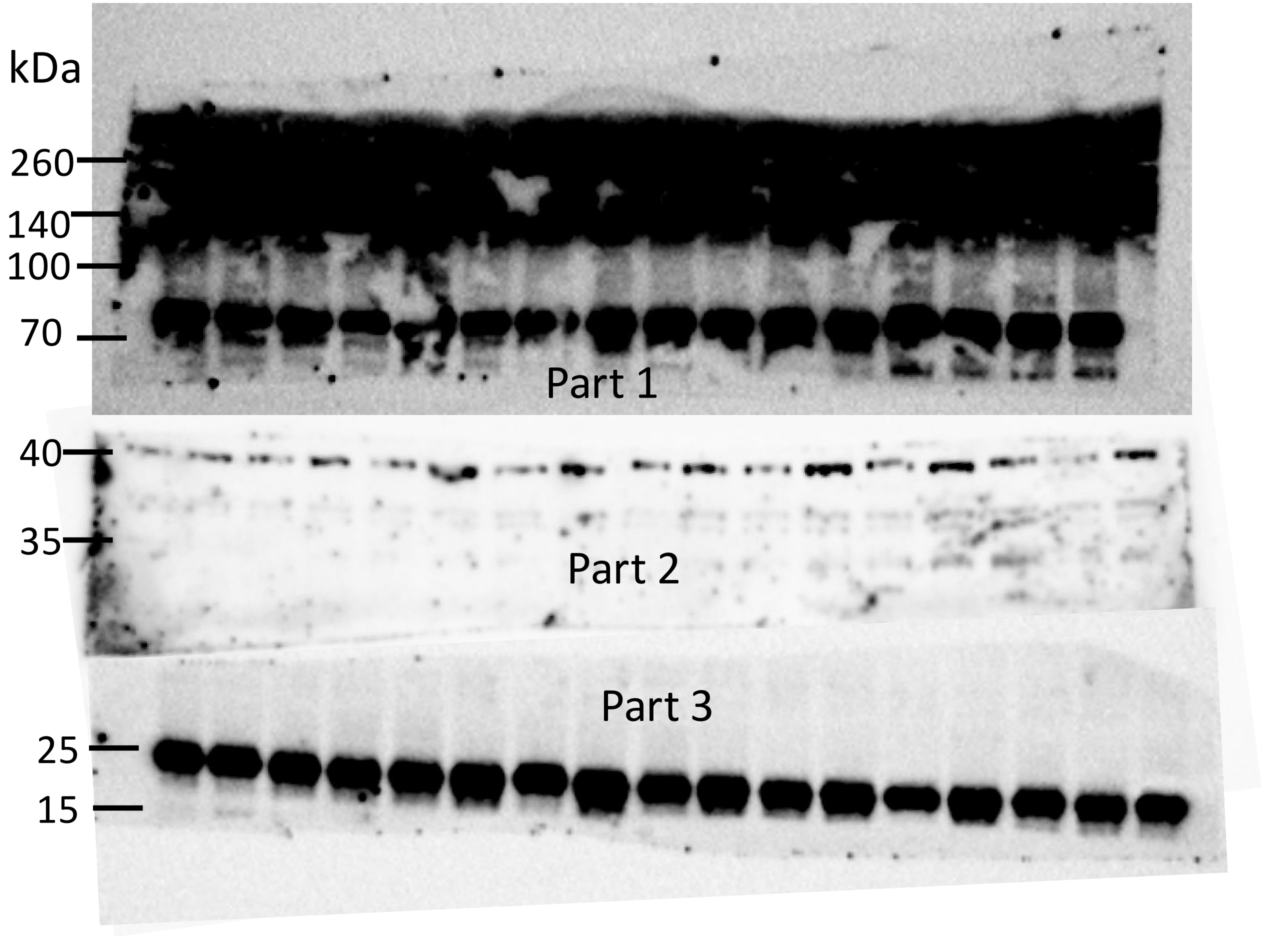

Markers

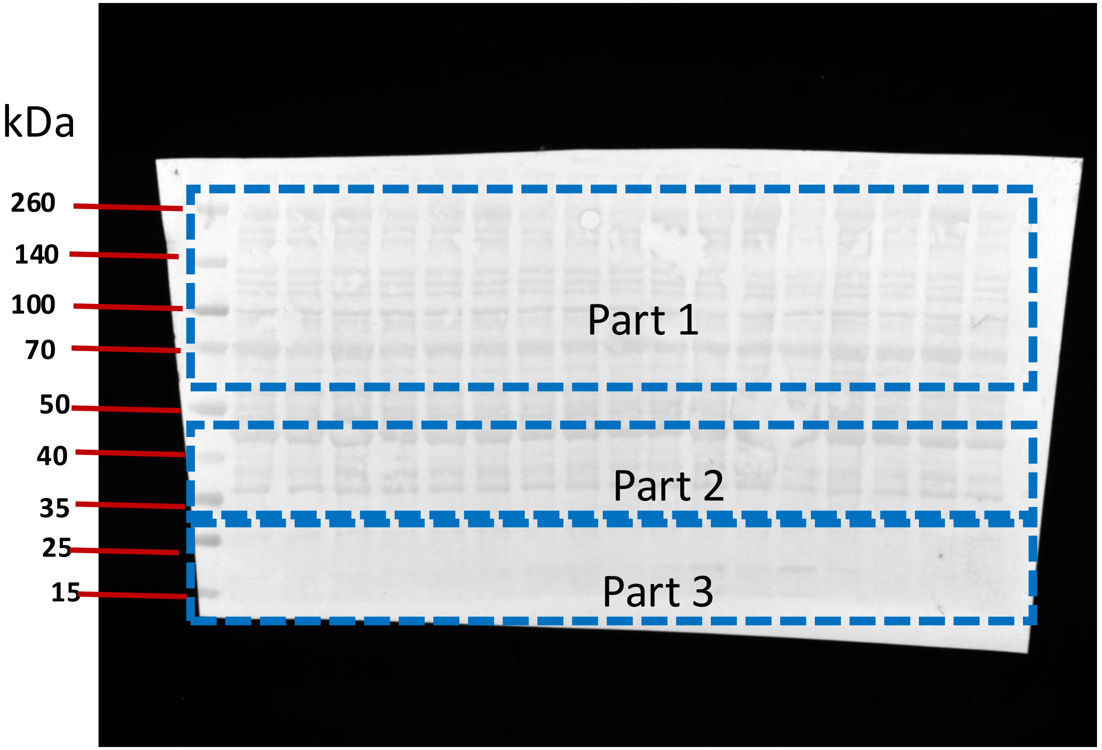

APT1, zDHHC5 and zDHHC4 all taken from the same membrane, which was cut to blot for the multiple targets

Figure 3: zDHHC5 (MW 78 kDa)

zDHHC4 (44 kDa), APT1 (25 kDa) and zDHHC5 (78kDa) all cut from the same membrane – same Ponceau housekeeper

Chemi

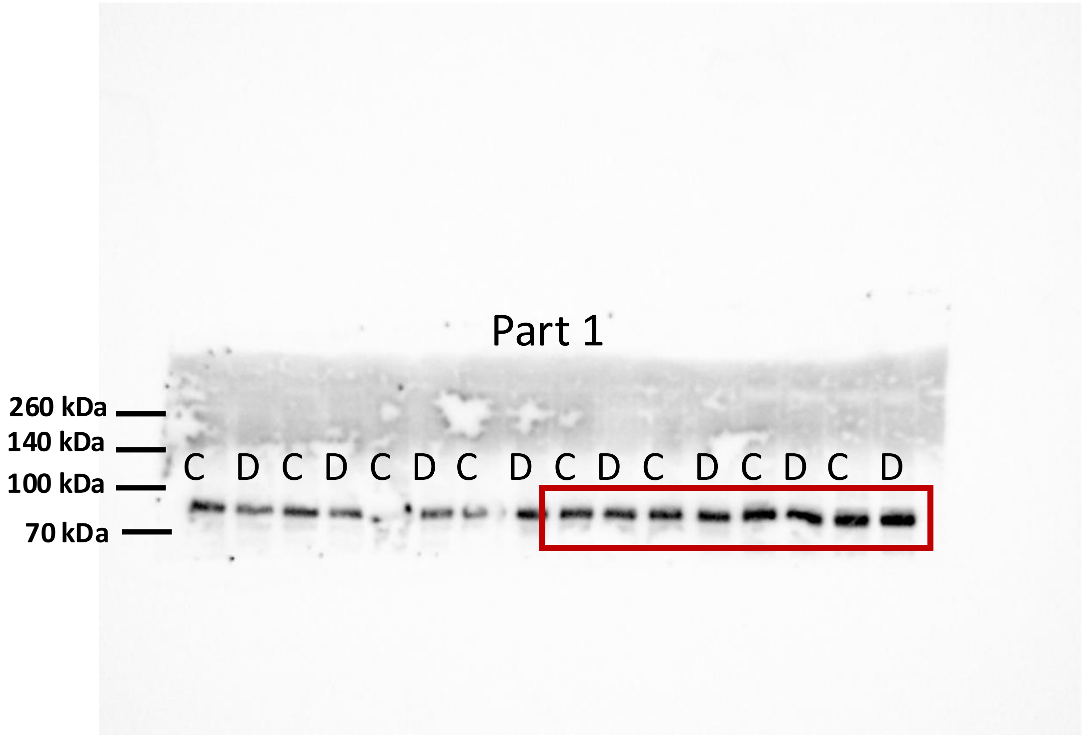

Markers

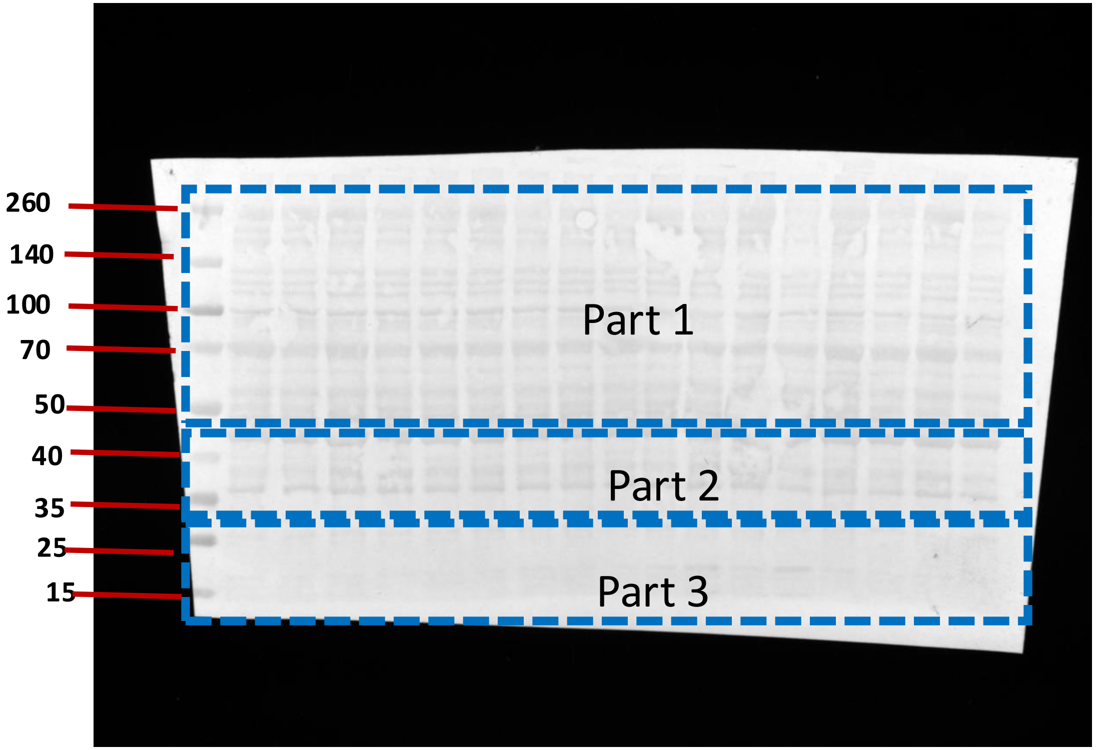

Figure 3: zDHHC4 (MW: 40 kDa)

zDHHC4 (44 kDa), APT1 (25 kDa) and zDHHC5 (78kDa) all cut from the same membrane – same Ponceau housekeeper

Quantified image – from Ponceau

Chemi

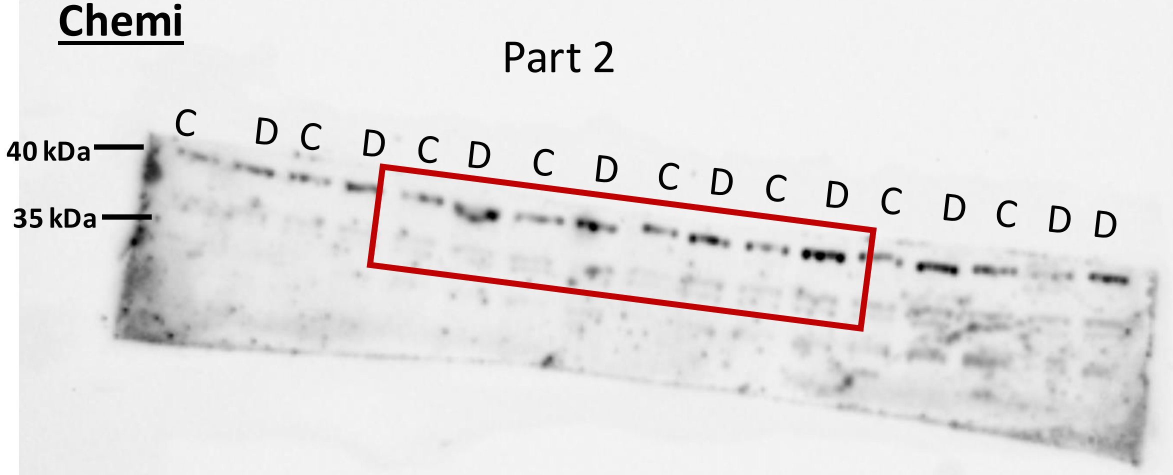

Replicate – not used for graph but still significant between groups

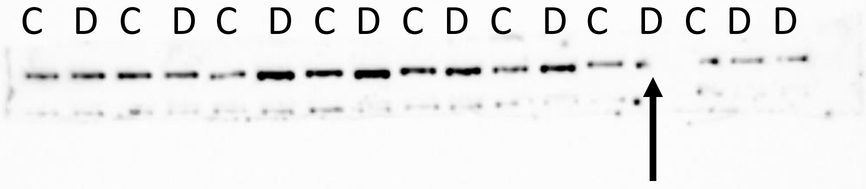

Sample removed due to air bubble on membrane

Markers

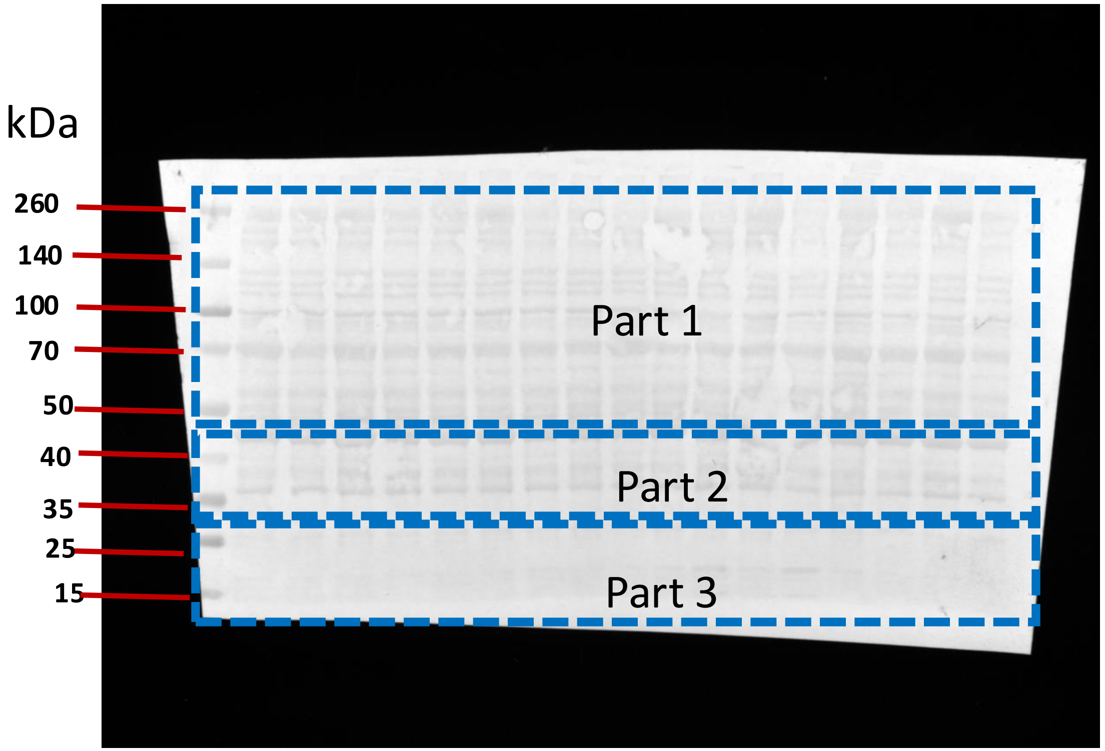

Figure 3: APT1 (MW 25 kDa)

zDHHC4 (44 kDa), APT1 (25 kDa) and zDHHC5 (78kDa) all cut from the same membrane – same Ponceau housekeeper

Chemi

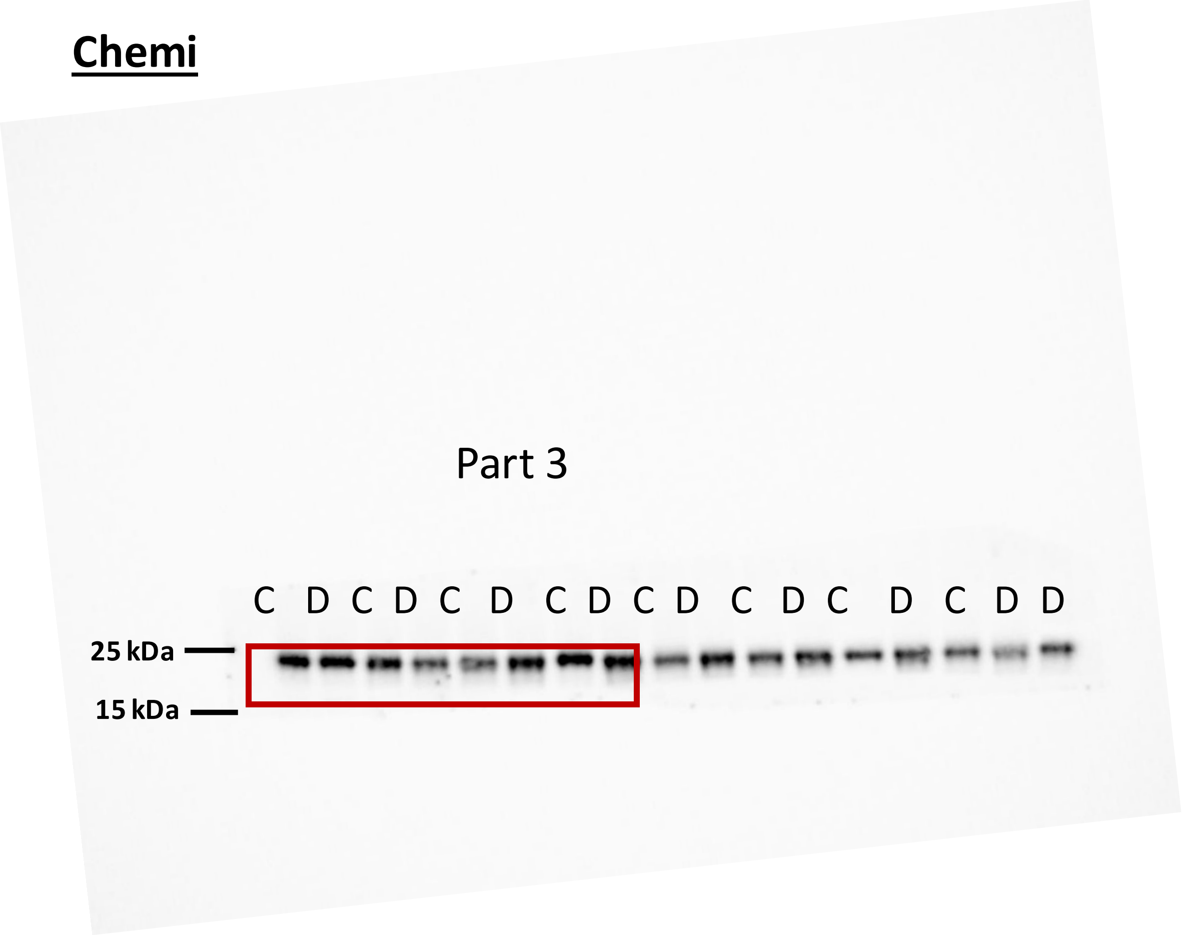

Markers

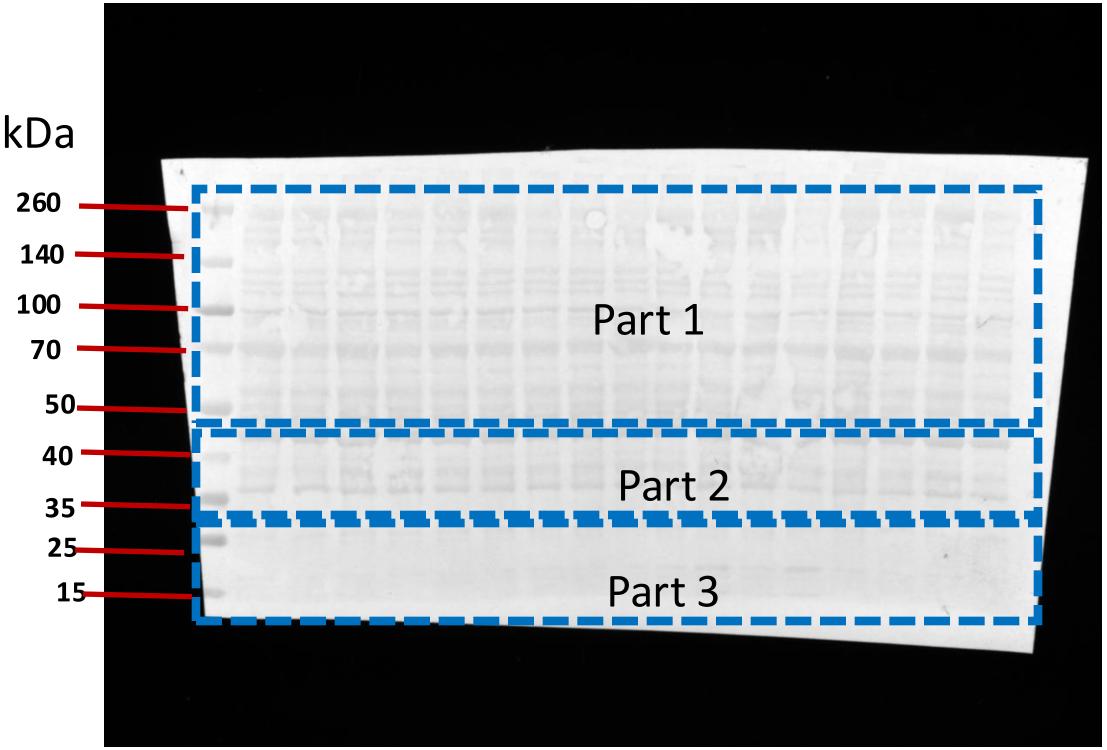

APT1, zDHHC5 and zDHHC4 all taken from the same membrane, which was cut to blot for the multiple targets

Figure 1 E and F: Sarcolemmal CD36 membrane 1 of 2

4 gels transferred on to one membrane – due to the large number of fractions generated per heart.

Gels cut just above the 140 Mw band and below the 50 Mw band – then all transferred on to the same membrane, to minimize heterogeneity between samples through transfer, antibody incubation and developing.

Chemi

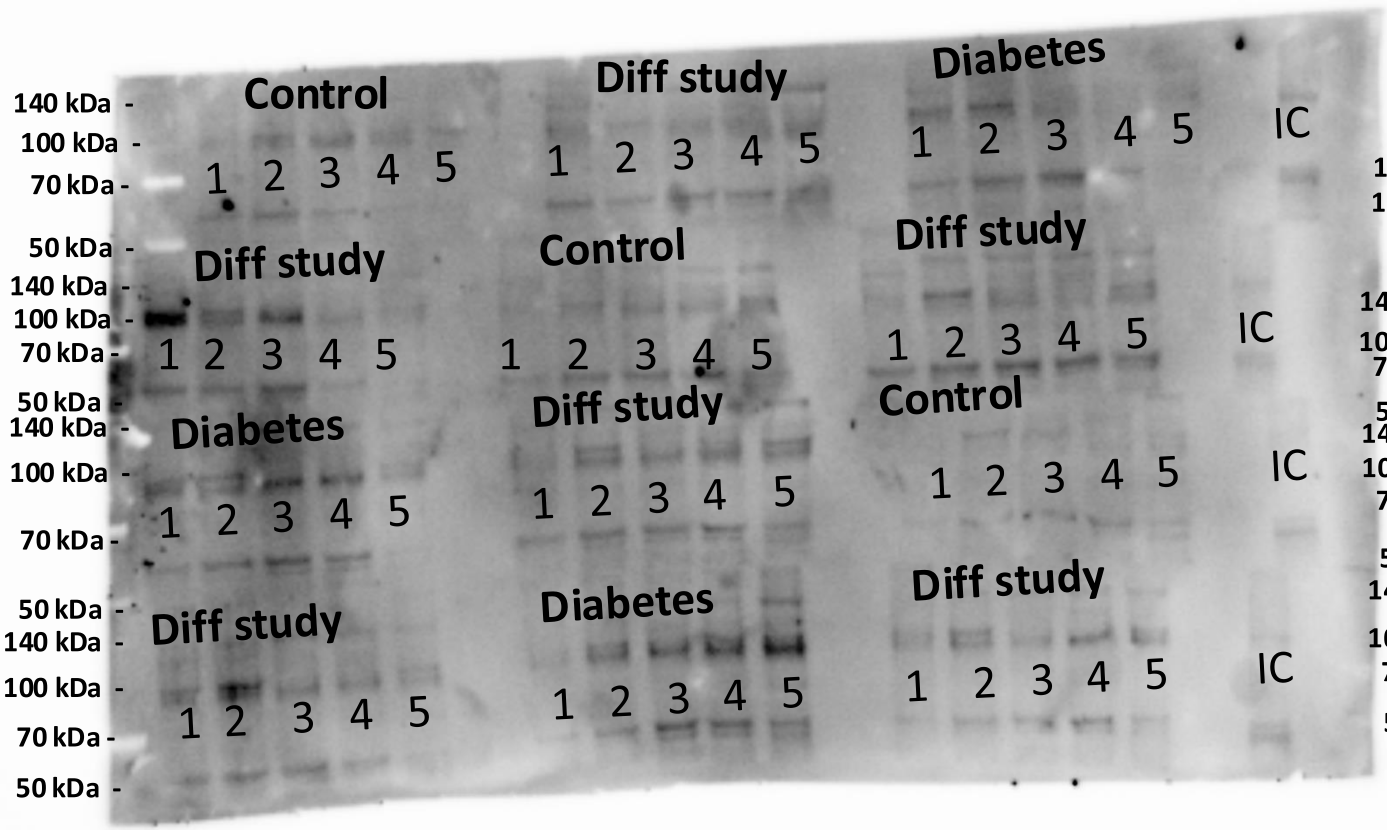

Marker

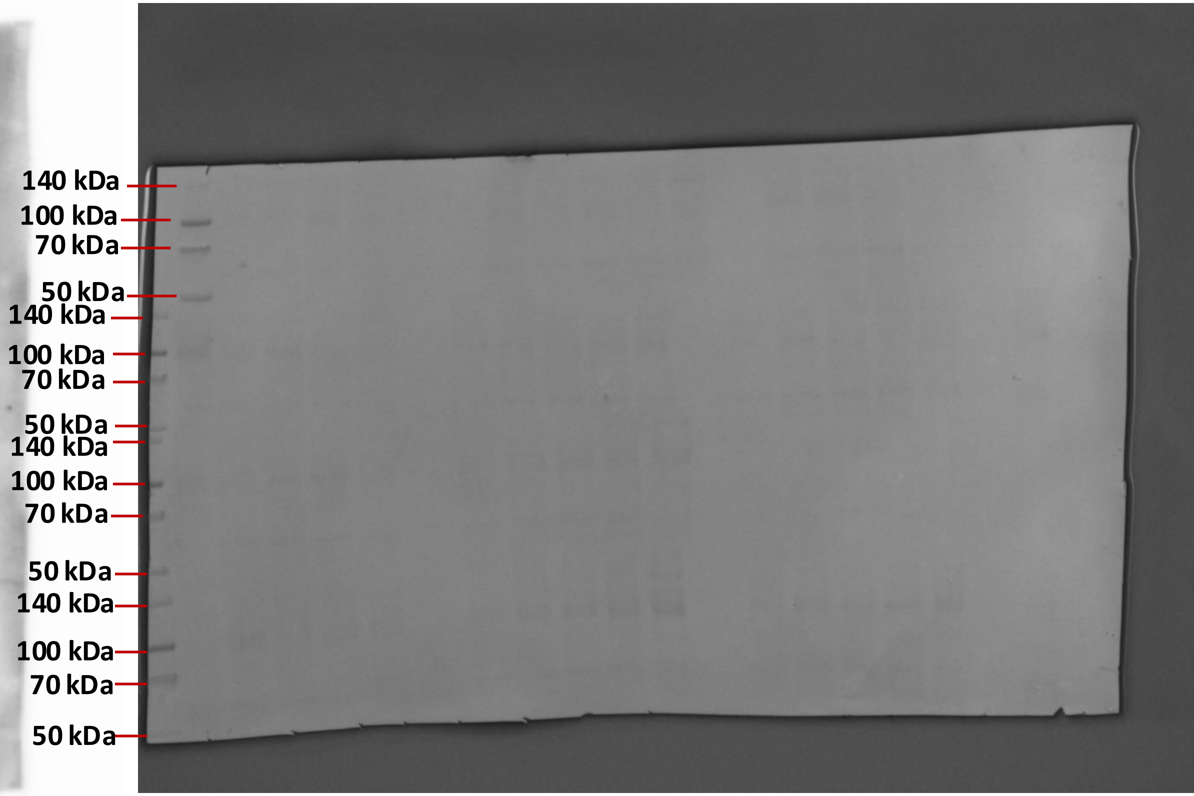

"Diff Study" = samples analysed for a different study

Figure 1 E and F: Sarcolemmal CD36 membrane 2 of 2

4 gels transferred on to one membrane – due to the large number of fractions generated per heart.
Gels cut just above the 140 Mw band and below the 50 Mw band – then all transferred on to the same membrane, to minimize heterogeneity between samples through transfer, antibody incubation and developing.

Chemi

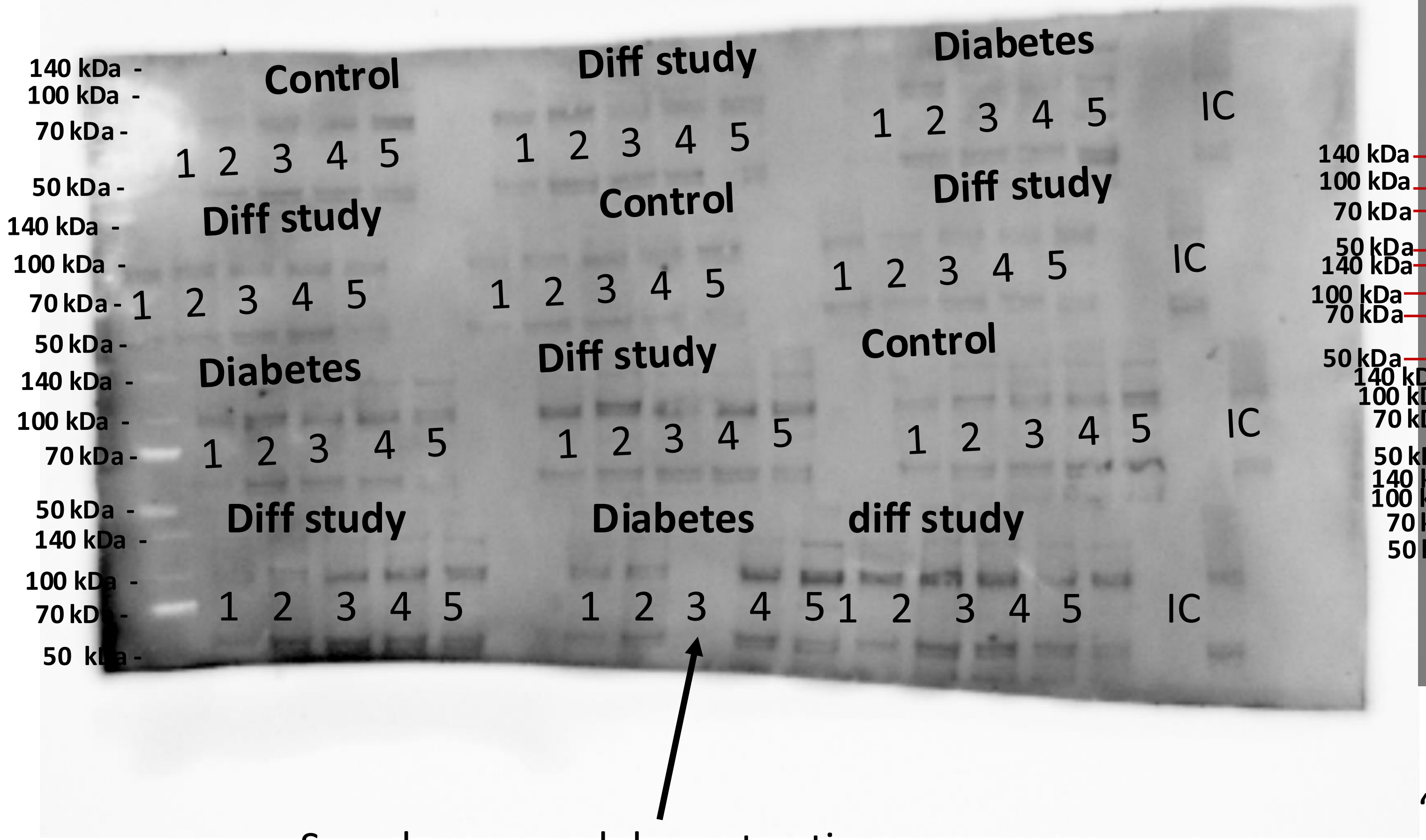

Sample removed due extraction error as no protein present in P3

Marker

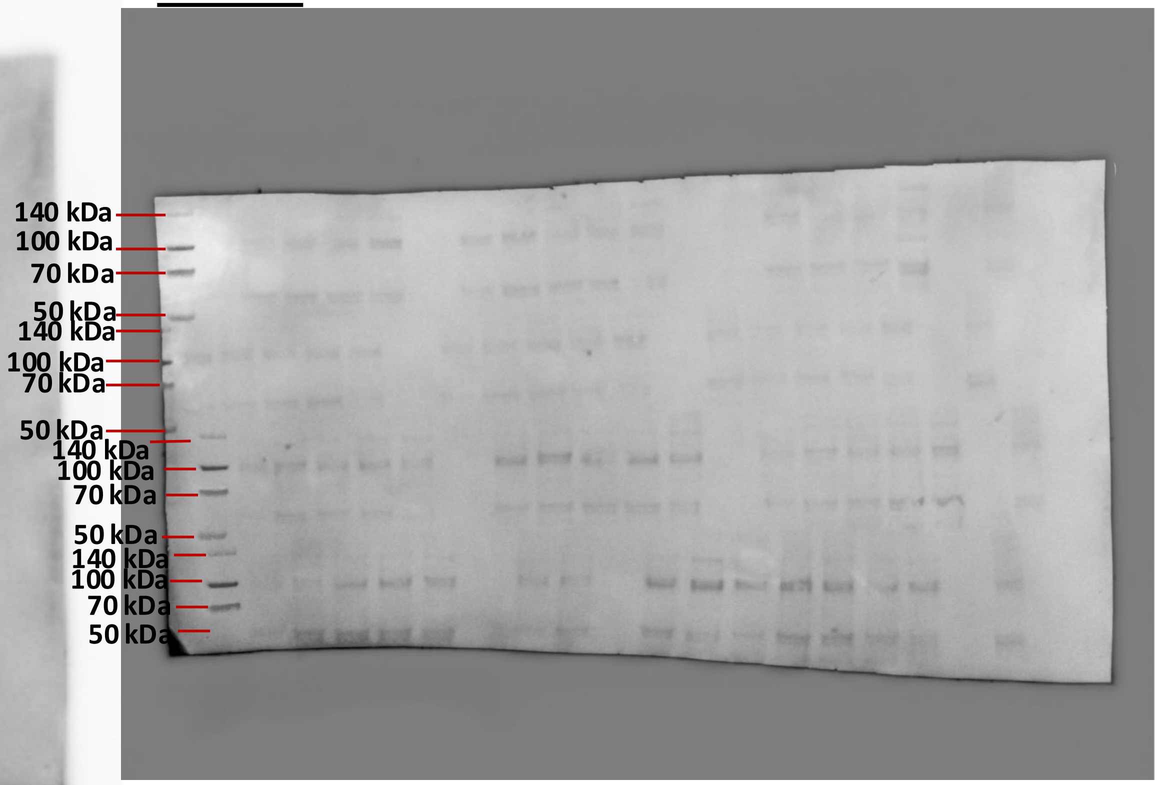

"Diff Study" = samples analysed for a different study
"IC" =internal control

Figure 2 A: CD36 S-acylation Rat

Chemi

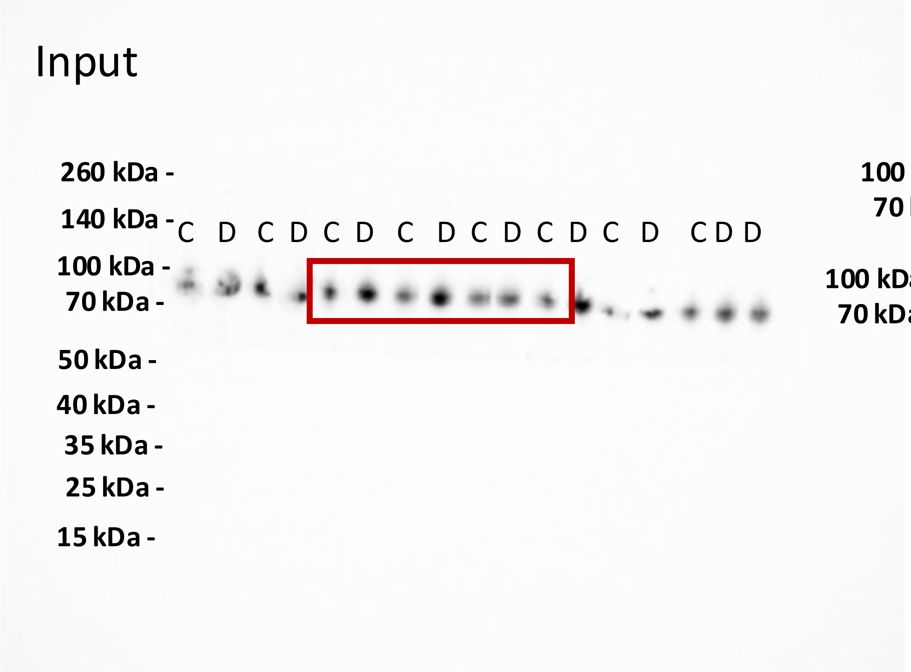

Uncropped gel and membrane

Chemi

CD36 S-acylation

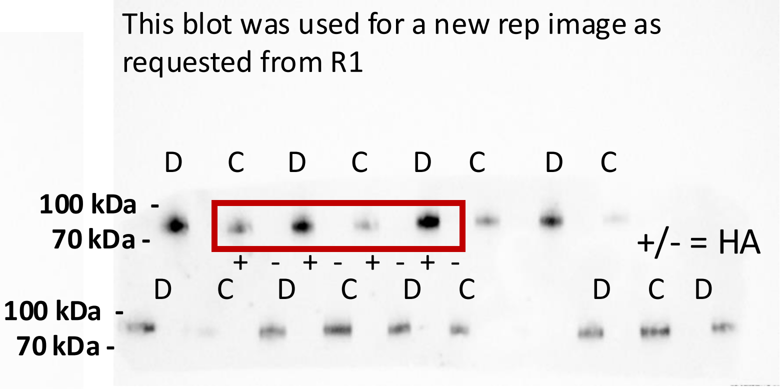

Chemi

CD36 S-acylation

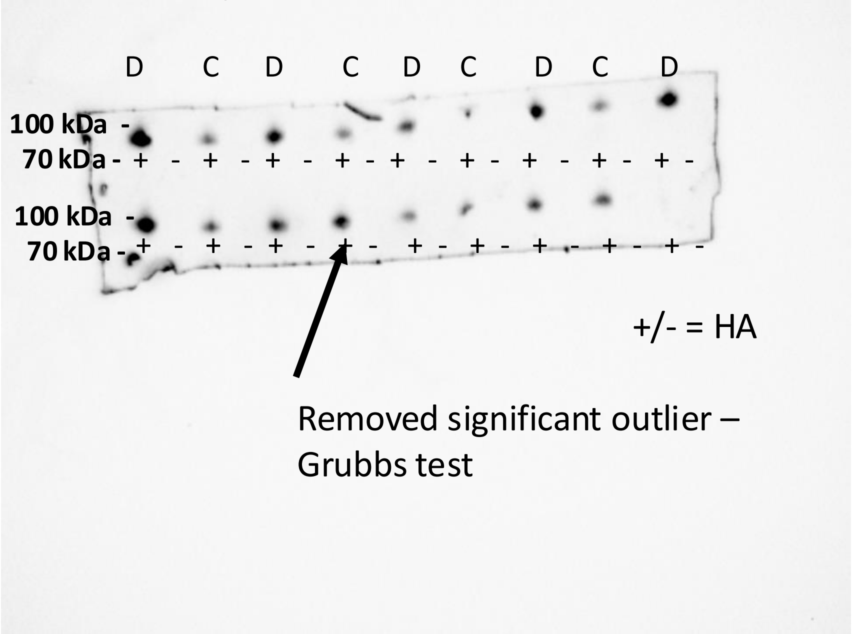

2 gels transferred on to one membrane – due to the positive (+HA) and negative (-HA) sample needed for every heart

Gels cut at the 140 Mw band and below the 70 Mw band – then all transferred on to the same membrane, to minimize heterogeneity between samples through transfer, antibody incubation and developing.

Figure 2C: Acyl PEG CD36

Chemi

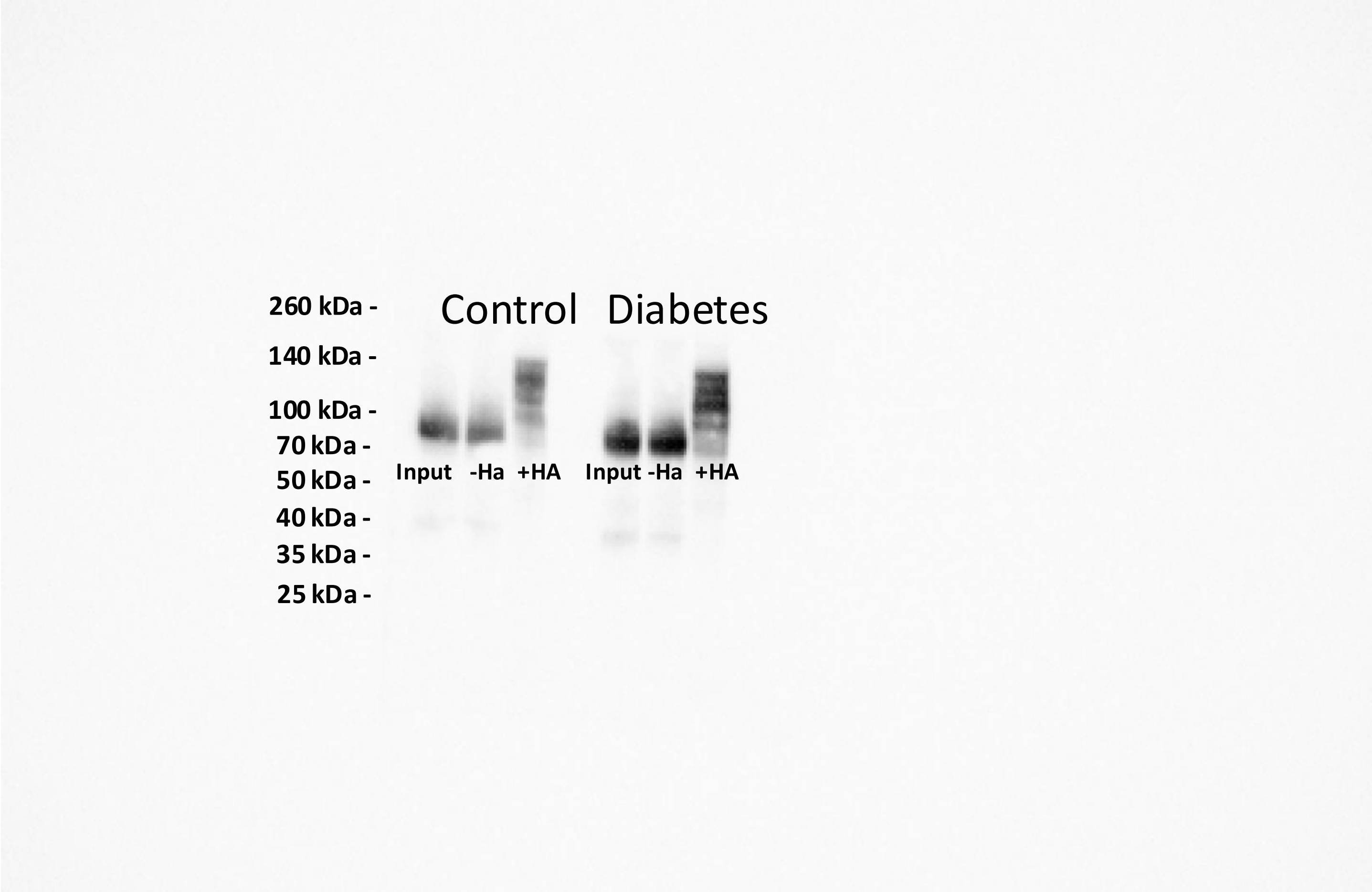

Uncropped gel and membrane

Figure 2 D: CD36 S-acylation Pig

Chemi

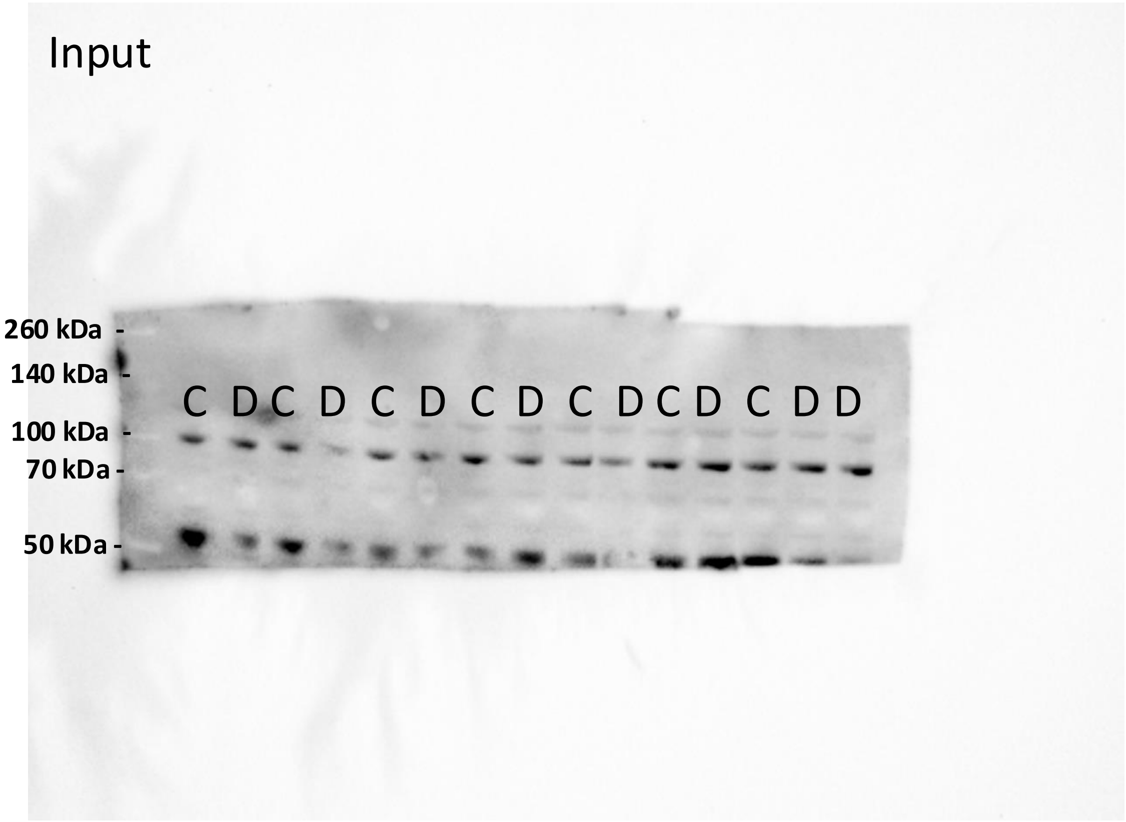

Chemi

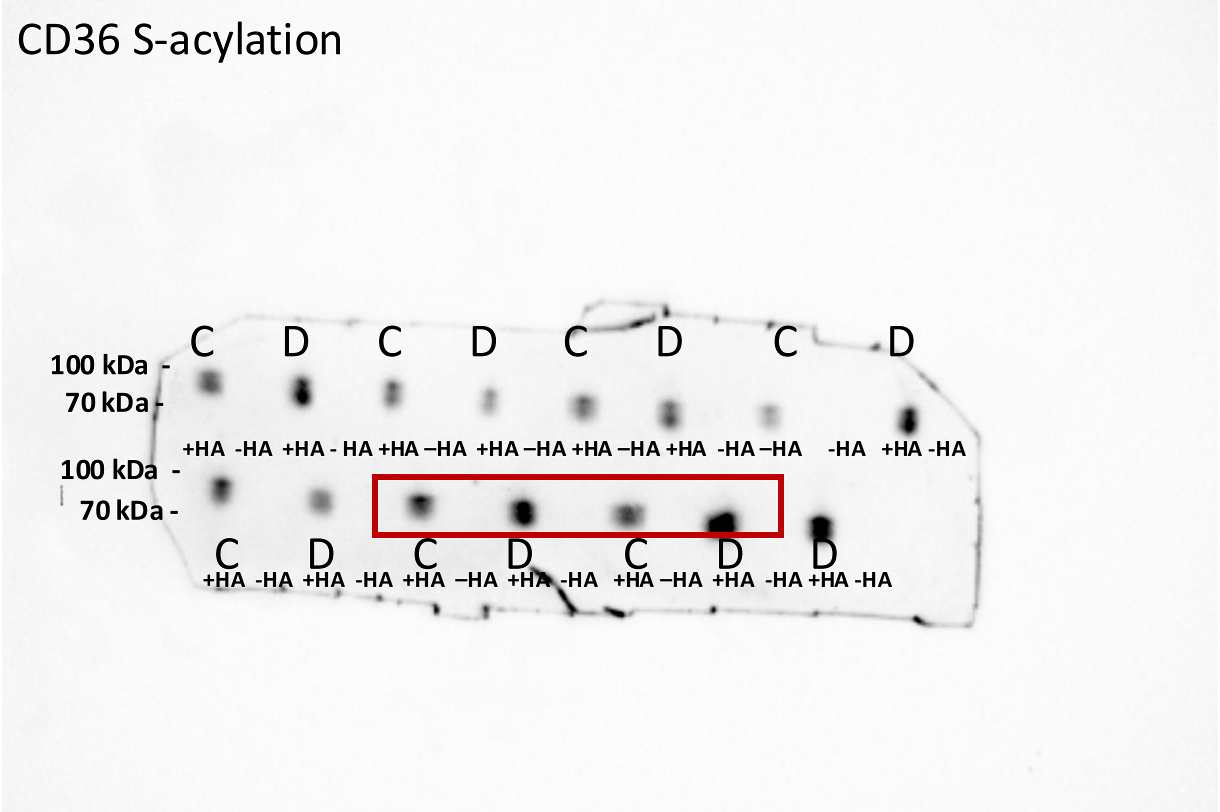

2 gels transferred on to one membrane – due to the positive (+HA) and negative (-HA) sample needed for every heart

Gels cut just above the 100 Mw band and below the 70 Mw band – then all transferred on to the same membrane, to minimize heterogeneity between samples through transfer, antibody incubation and developing.

Figure 2 E: CD36 S-acylation db/db

Chemi Input

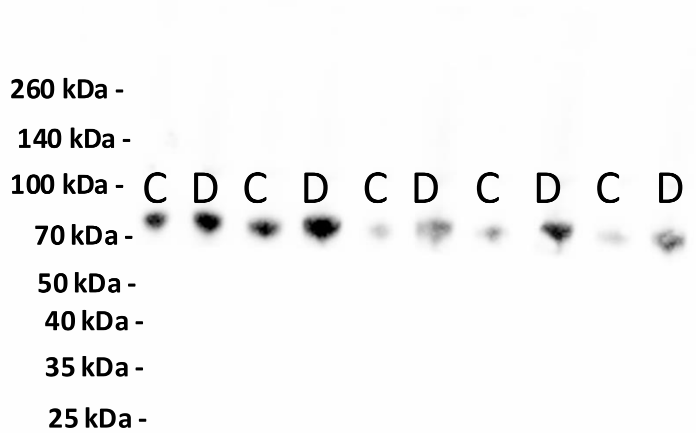

Uncropped gel and membrane

Chemi

CD36 S-acylation

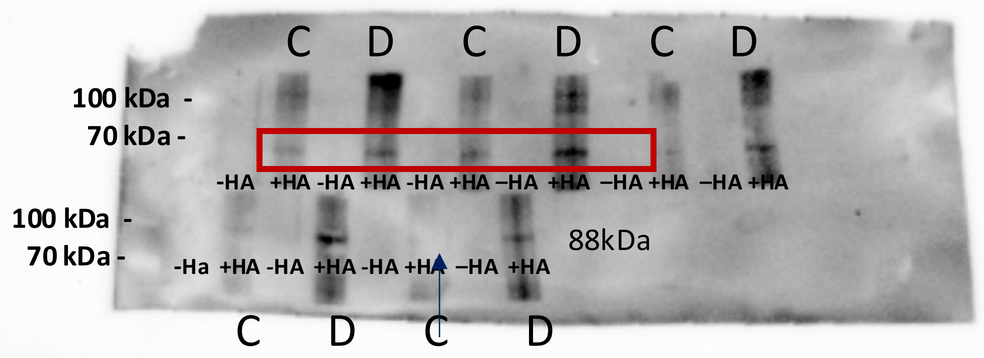

Sample removed – air bubble in blot

2 gels transferred on to one membrane – due to the positive (+HA) and negative (-HA) sample needed for every heart

Gels cut at the 140 Mw band and below the 70 Mw band – then all transferred on to the same membrane, to minimize heterogeneity between samples through transfer, antibody incubation and developing.

Figure 2F: CD36 S-acylation hiPSC-CM

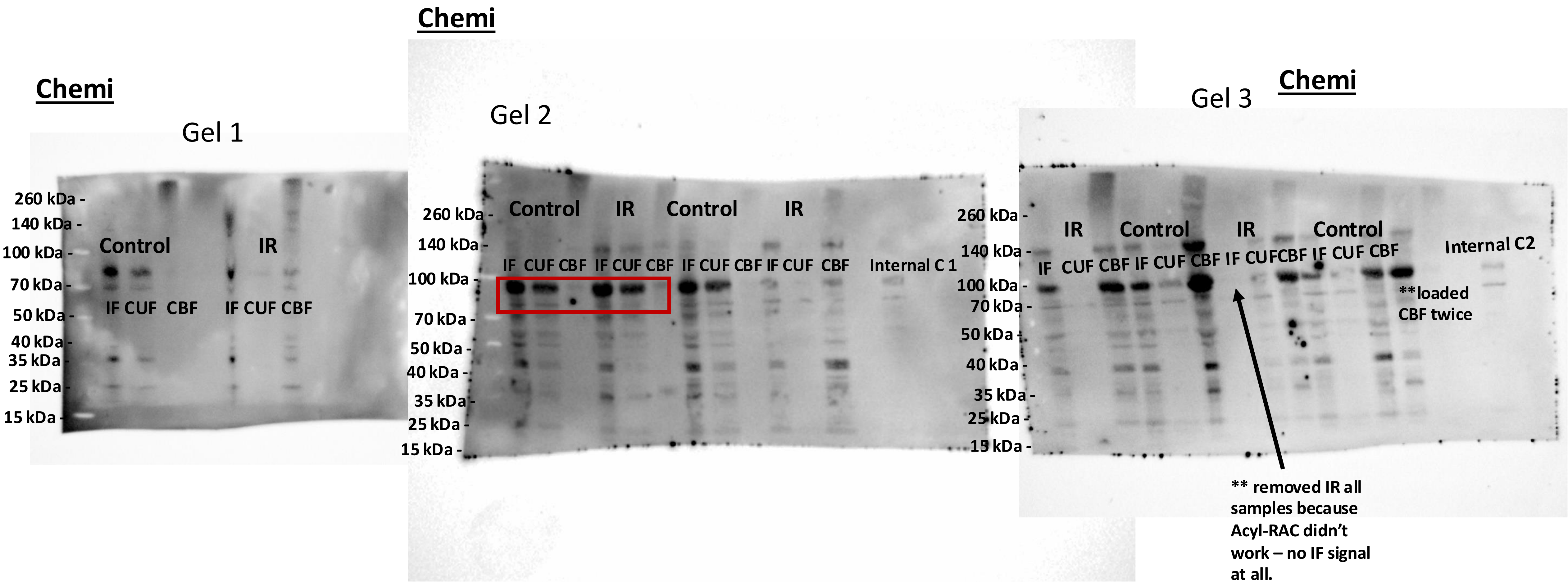

Uncropped gel and membrane

Figure 3 A-B: Low Fat High Fat

Chemi

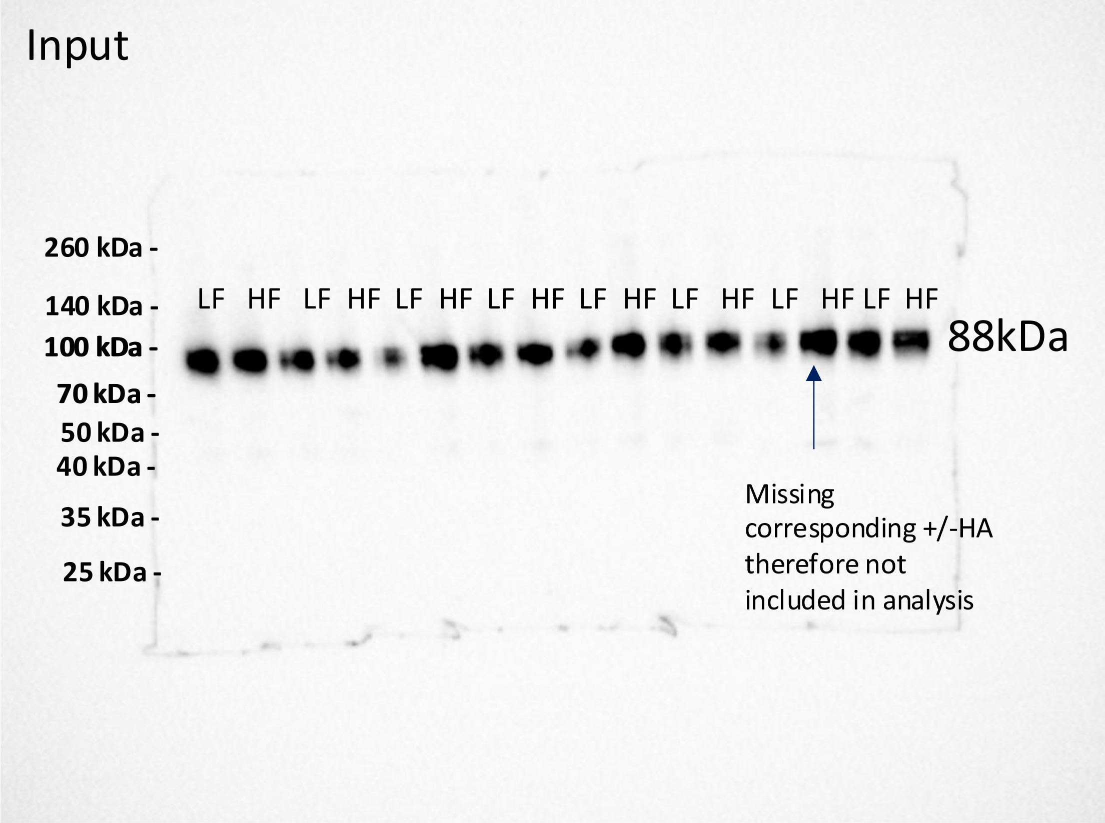

Uncropped gel and membrane

Chemi

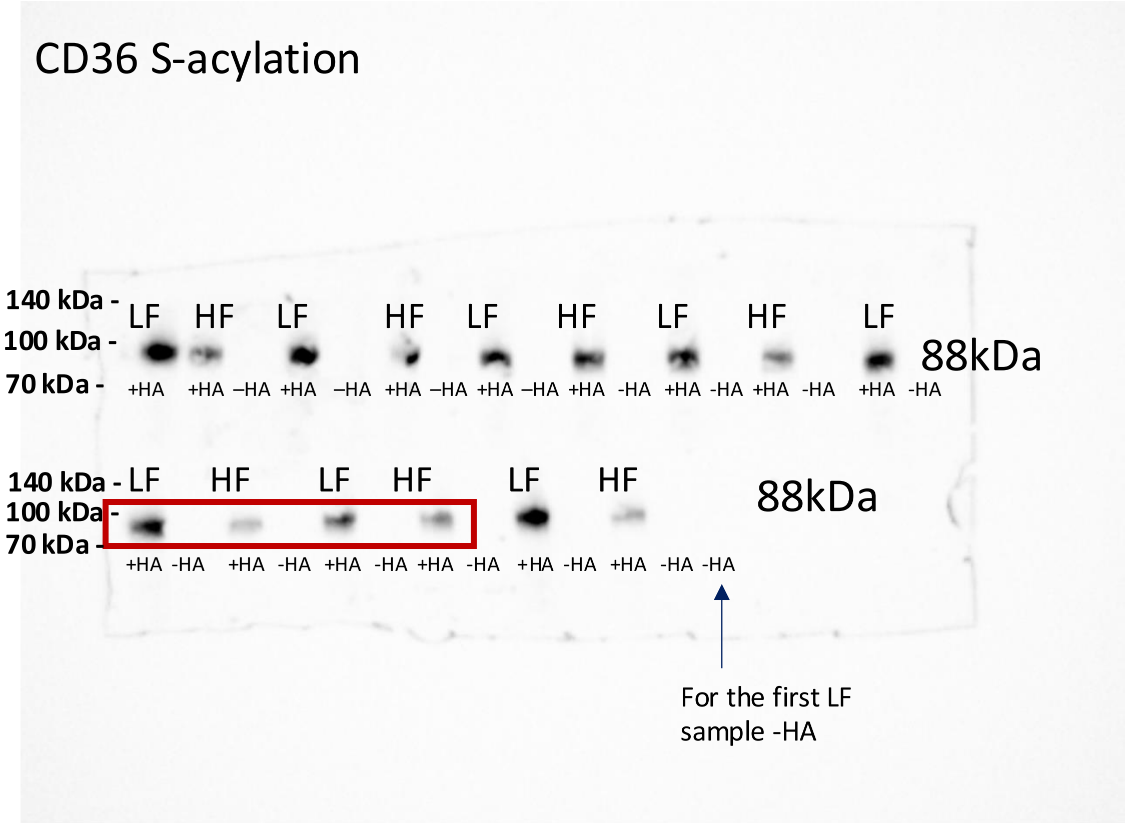

2 gels transferred on to one membrane – due to the positive (+HA) and negative (-HA) sample needed for every heart

Gels cut just at the 140 Mw band and below the 70 Mw band – then all transferred on to the same membrane, to minimize heterogeneity between samples through transfer, antibody incubation and developing.

Figure 5 D-E: hiPSC-CM CD36 S-acylation

Chemi Input

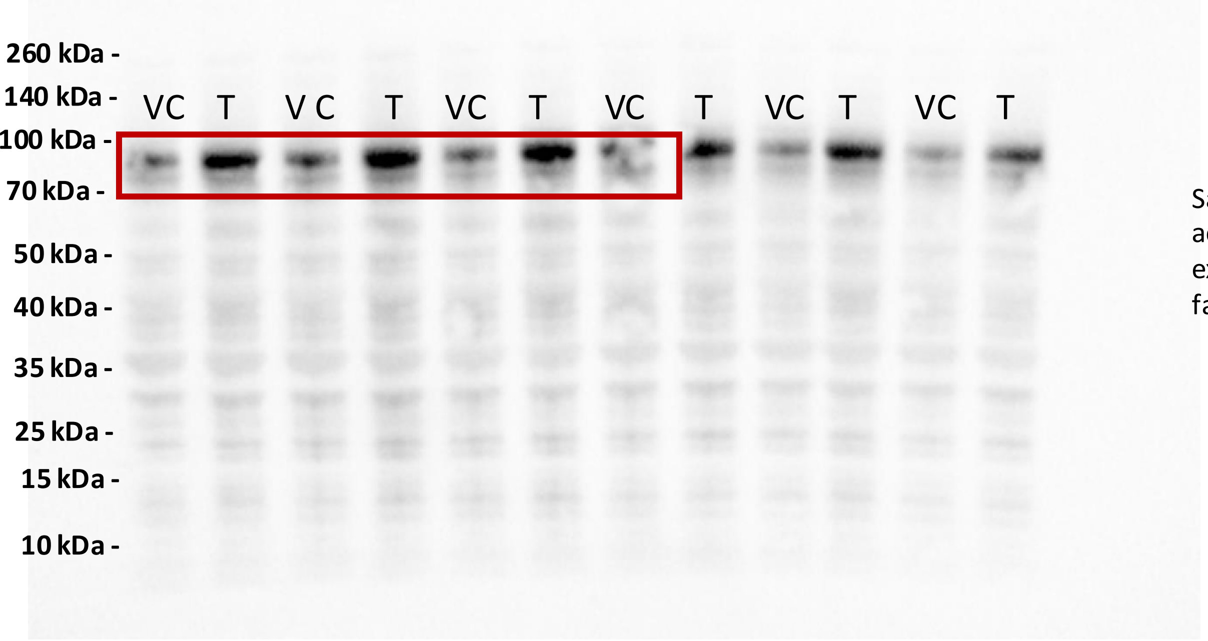

Uncropped gel and membrane

VC: Non-target siRNA

T:DHHC4 siRNA

2 gels transferred on to one membrane – due to the positive (+HA) and negative (-HA) sample needed for every heart

Gels cut just at the 140 Mw band and below the 70 Mw band – then all transferred on to the same membrane, to minimize heterogeneity between samples through transfer, antibody incubation and developing.

Chemi

CD36 S-acylation

Sample outlier using Grubbs test

Sample removed acyl-RAC experimental failure

Sample removed acyl-RAC experimental failure

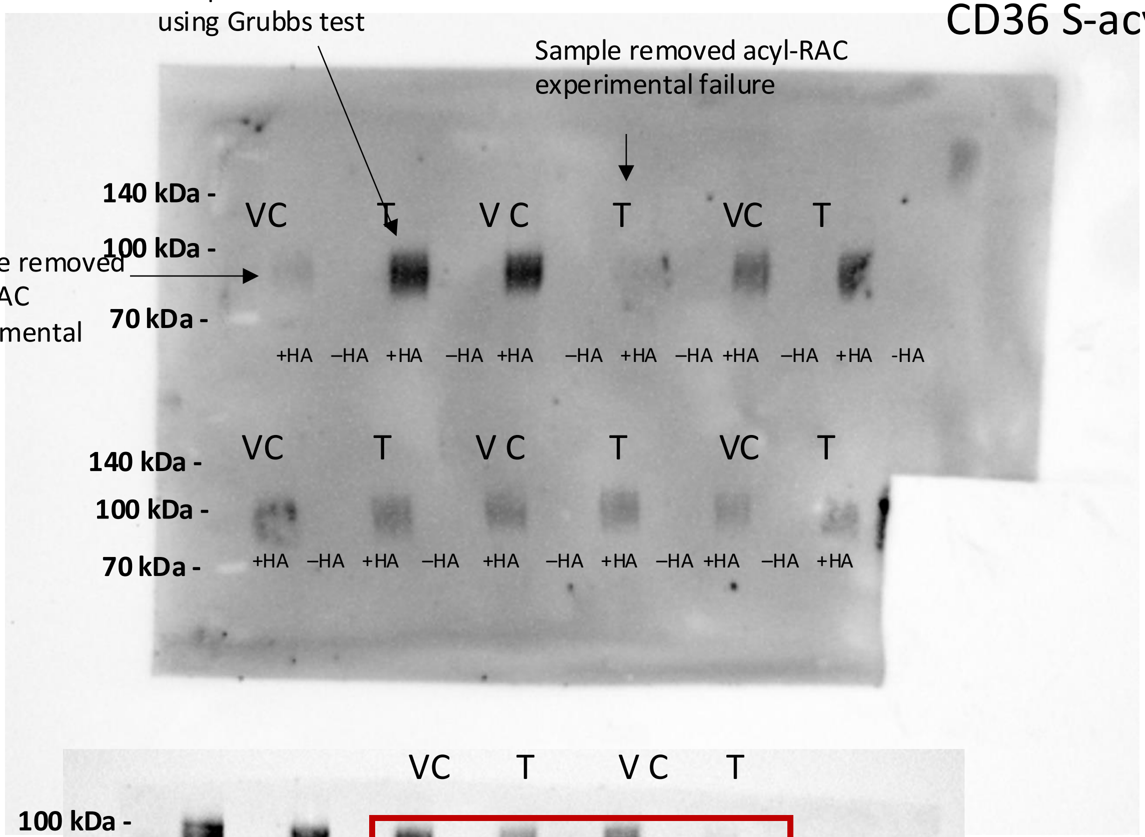

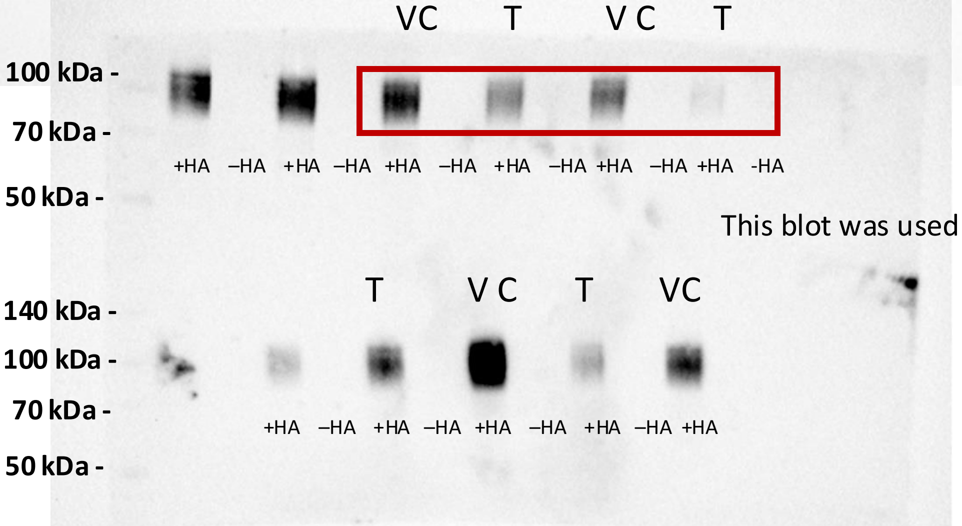

This blot was used for rep image

Figure 5 F-G: NRMV CD36 S-acylation

Chemi Input

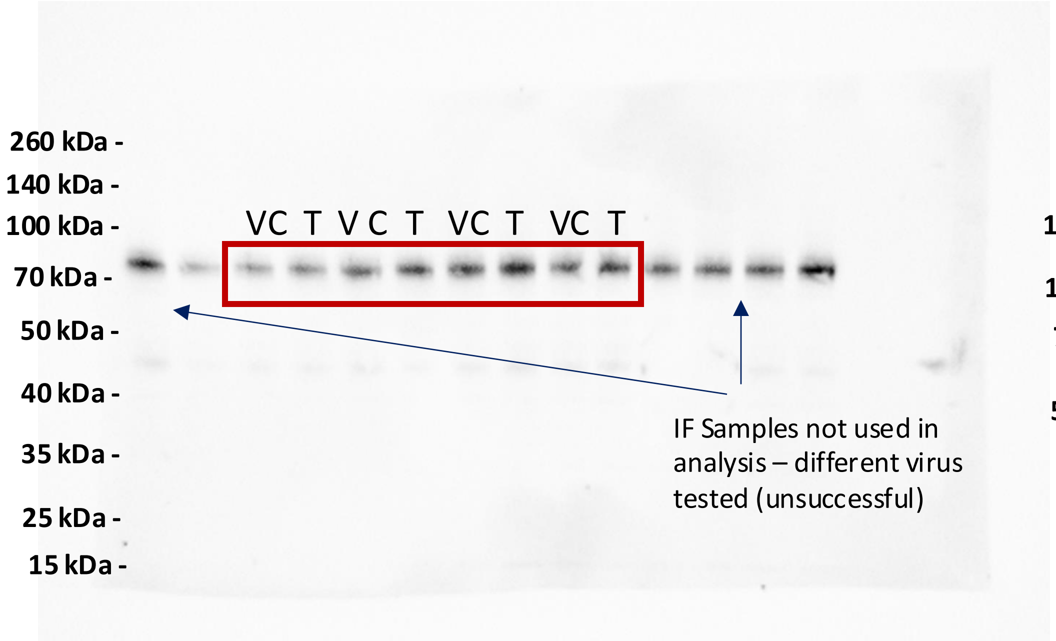

Uncropped gel and membrane

Chemi CD36 S-acylation

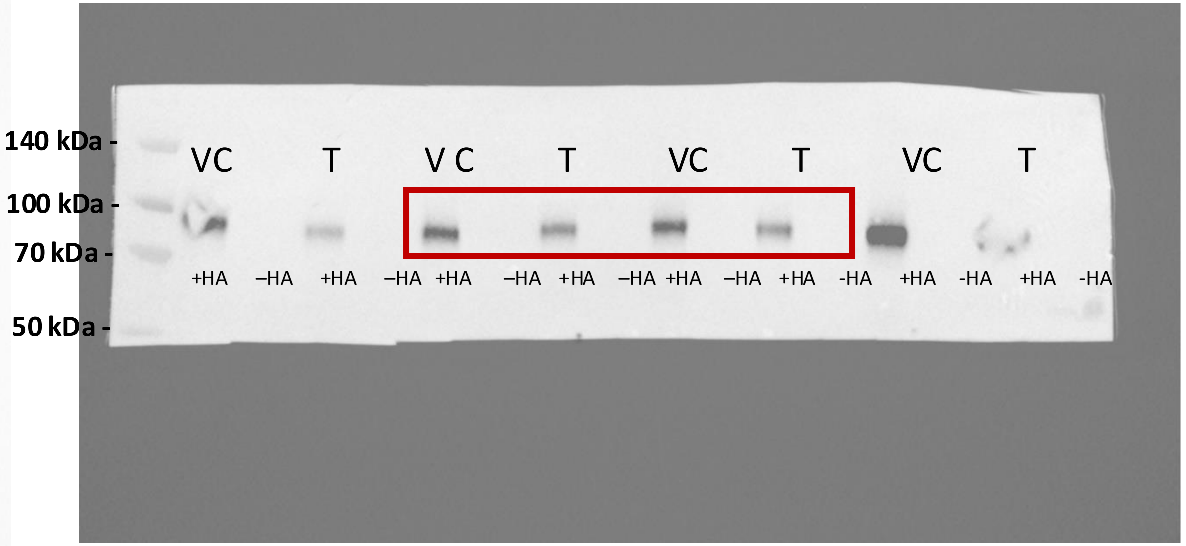

VC: Scramble ShRNA
T: DHHC4 ShRNA

Figure 5 I, J,L : FoxO1 KO and AS1842856 CD36 S-acylation input

Chemi Input

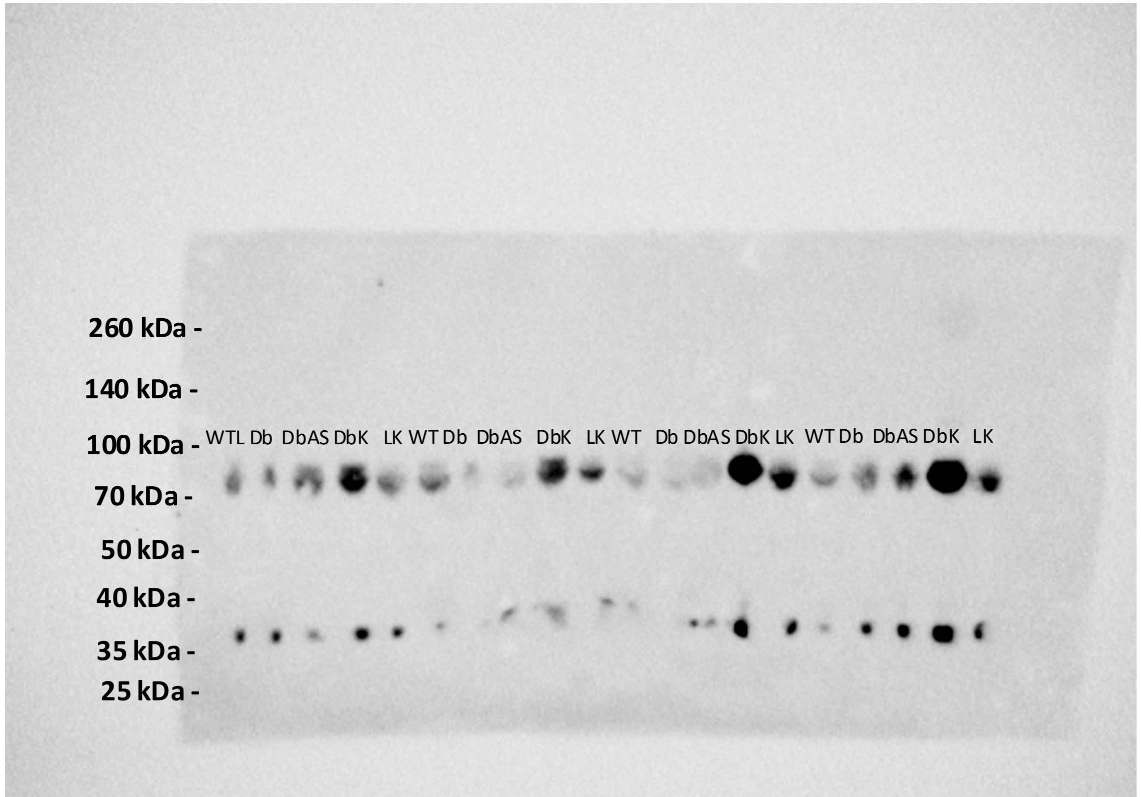

Uncropped gel and membrane

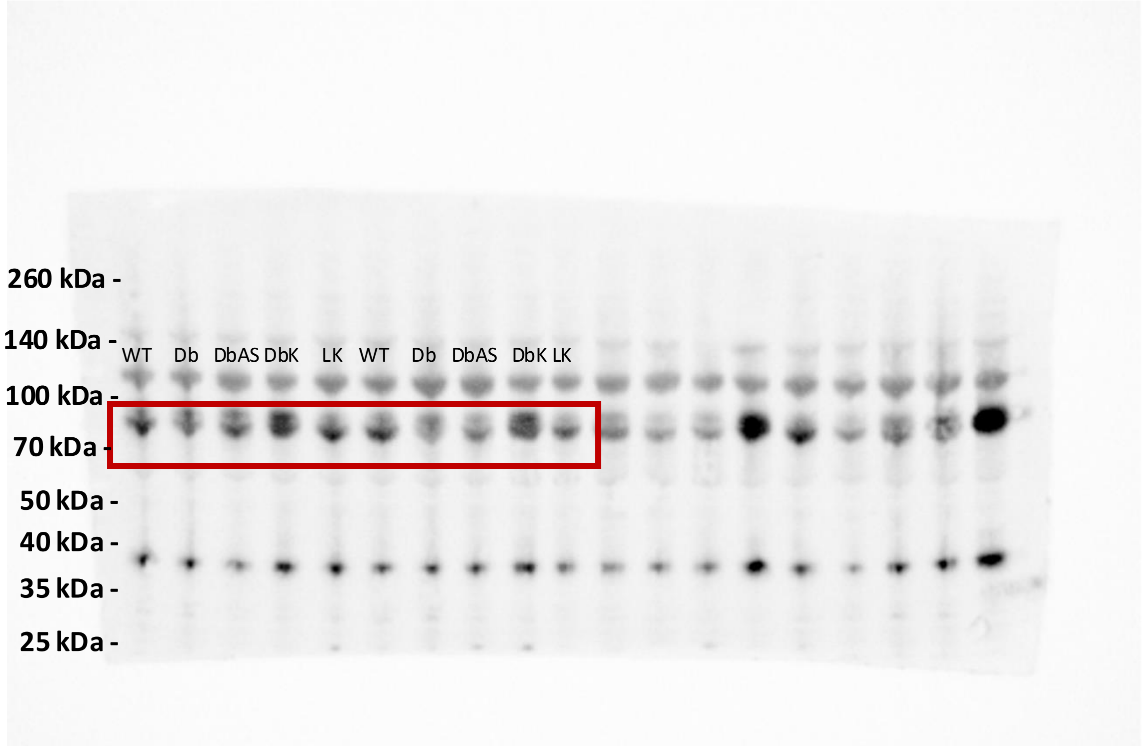

This blot was used for the rep image

Figure 5 I, J : FoxO1 KO CD36 S-acylation

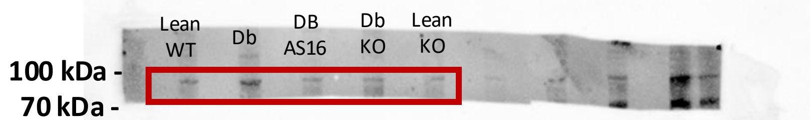

This blot was used for the rep image

Chemi

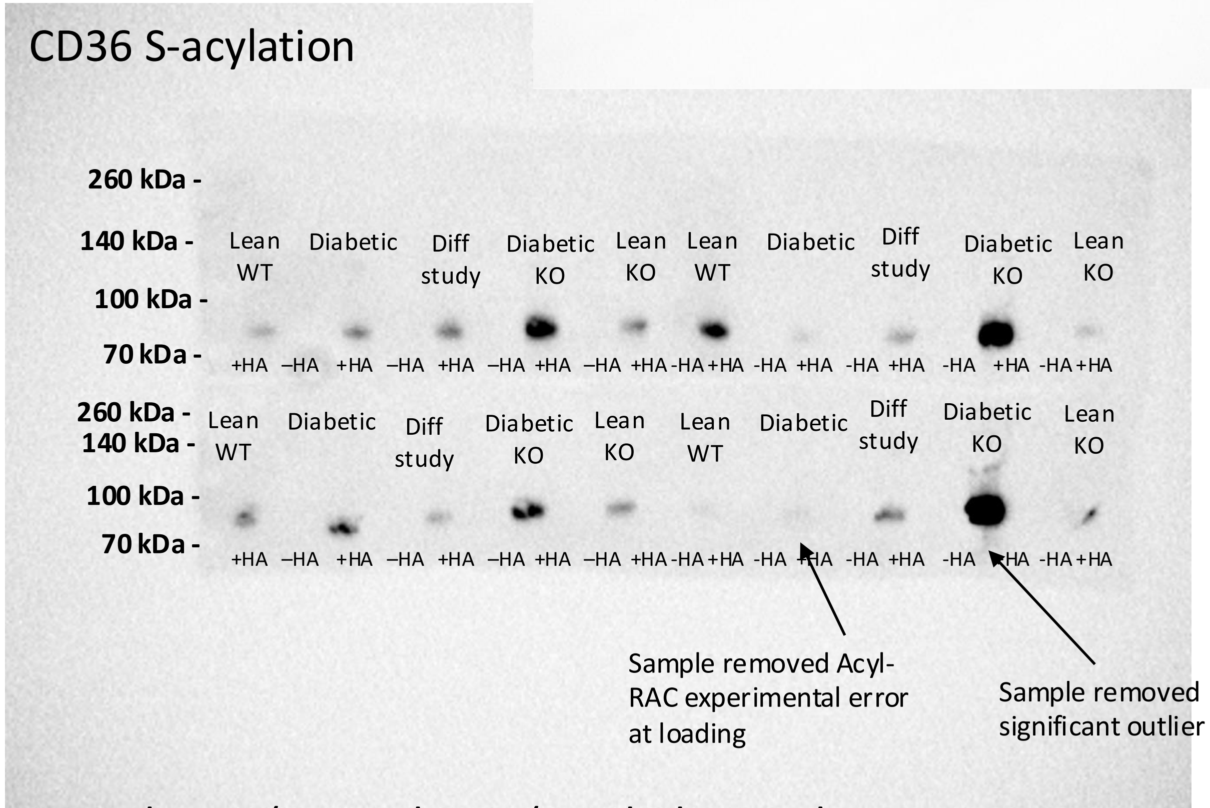

S-acylation (two gels cut (just below 70 kDa and above 260 kDa) and transferred on the same membrane). The membrane was cut after imaging

Marker

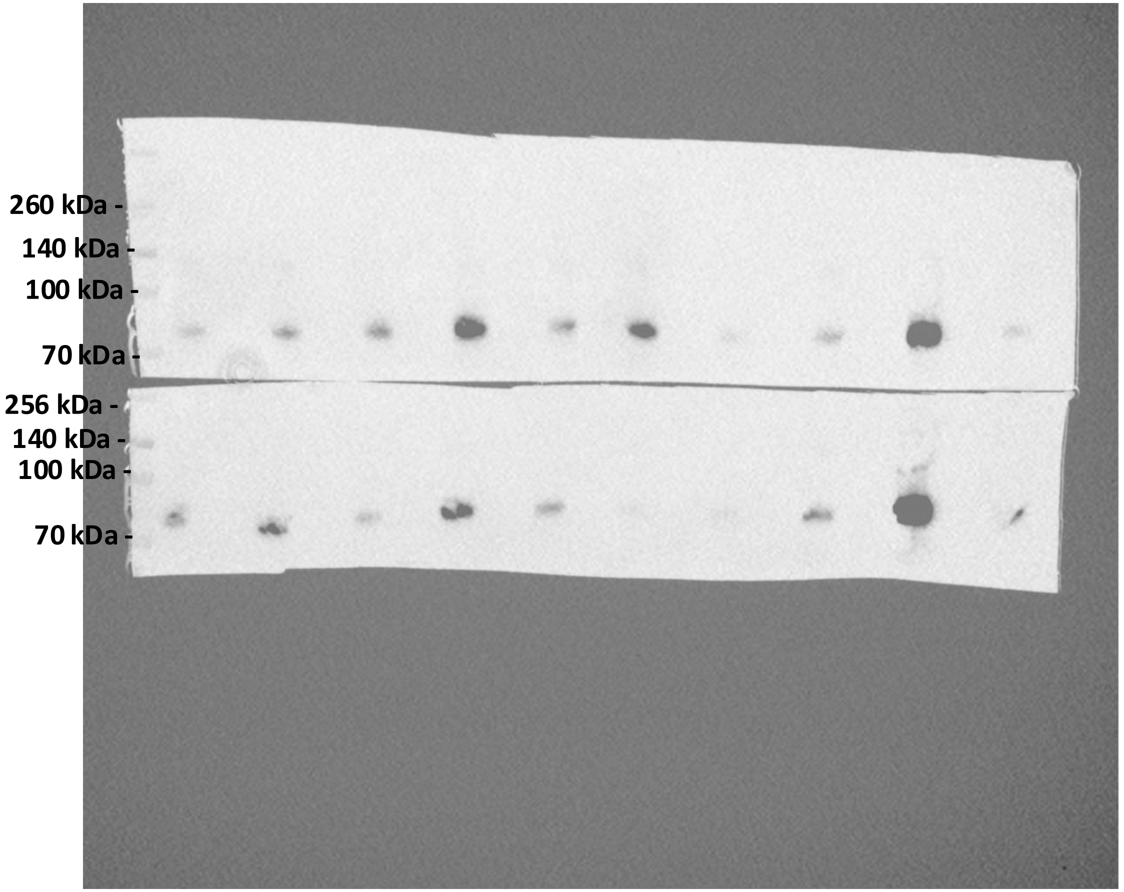

Figure 5 J, L: AS1842856 CD36 S-acylation

Chemi

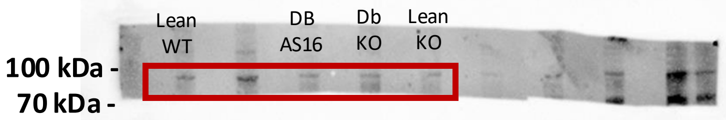

This blot was used for the rep image

CD36 S-acylation

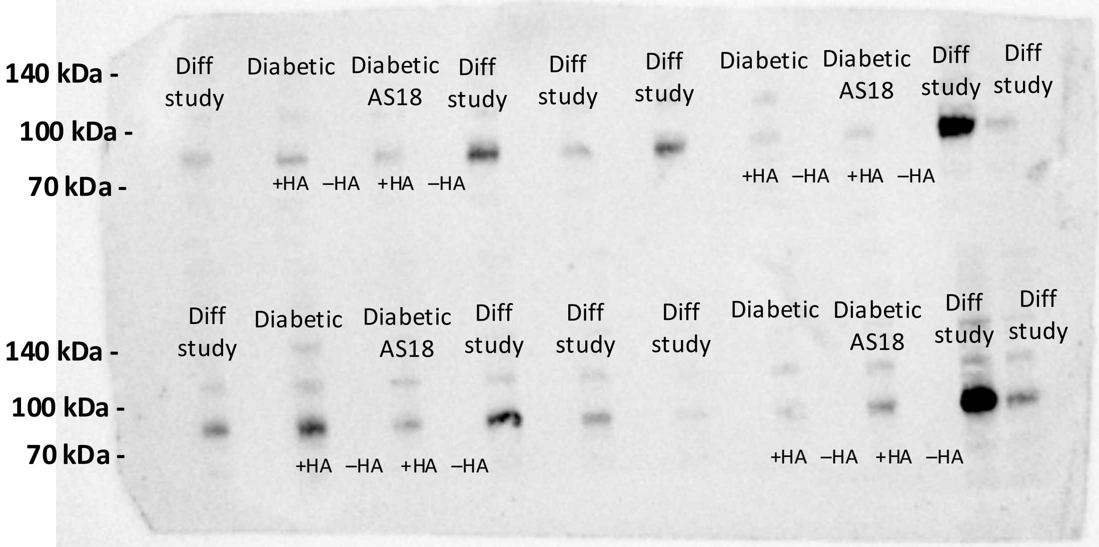

S-acylation (two gels cut (just below 70 kDa and above 140 kDa) and transferred on the same membrane).

Marker

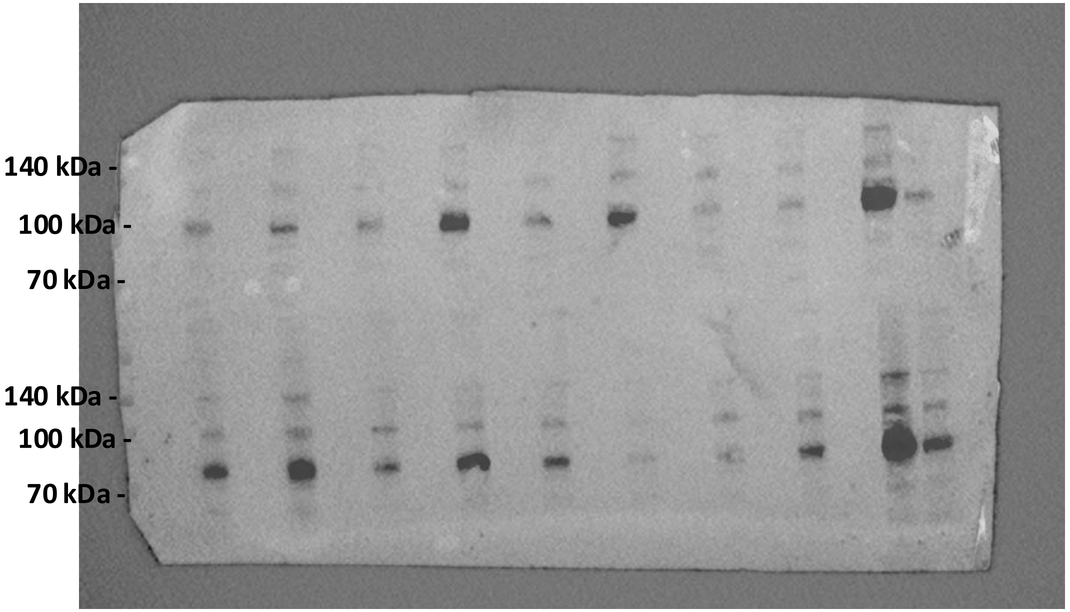

Figure 6B-C: CMA CD36 S-acylation

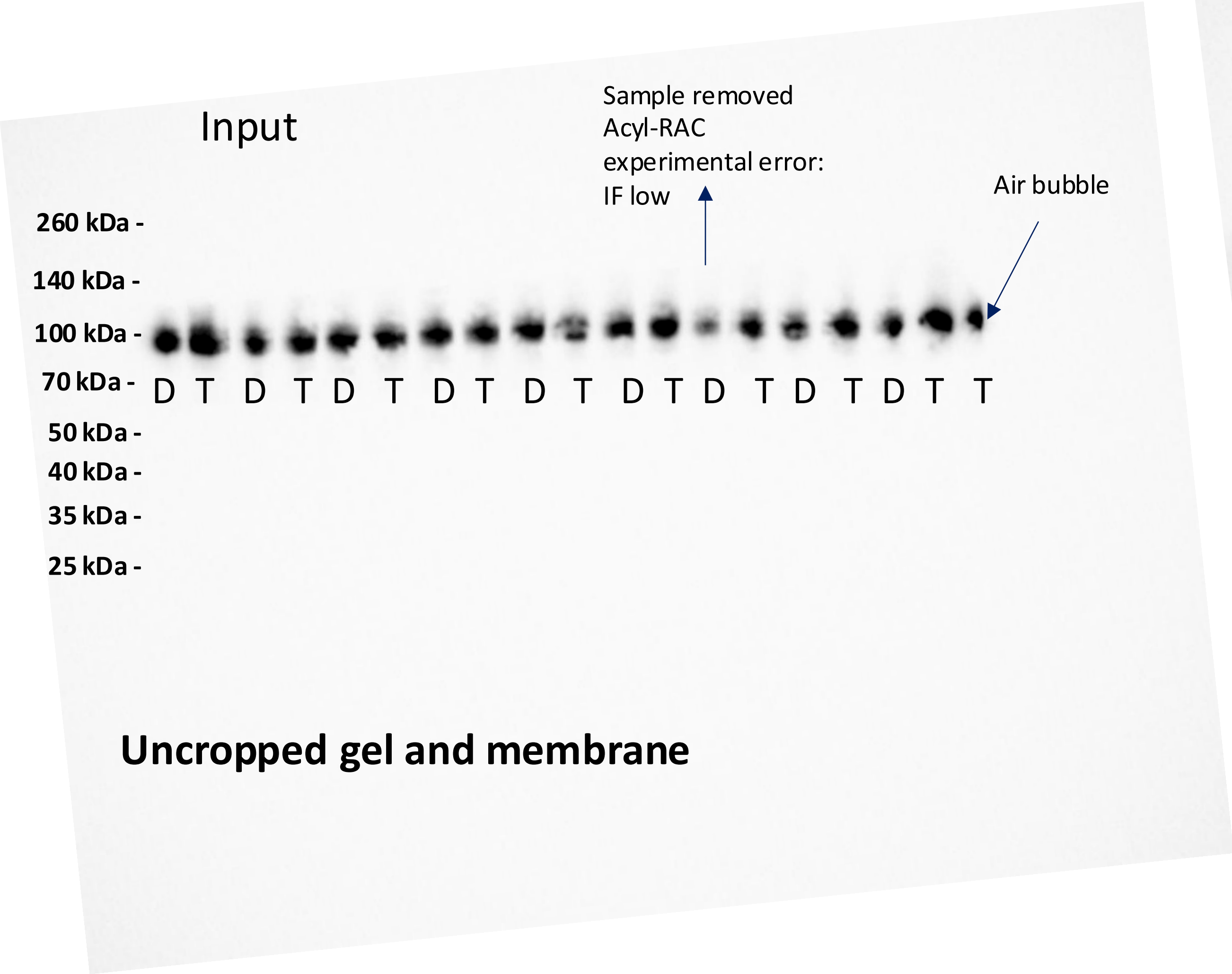

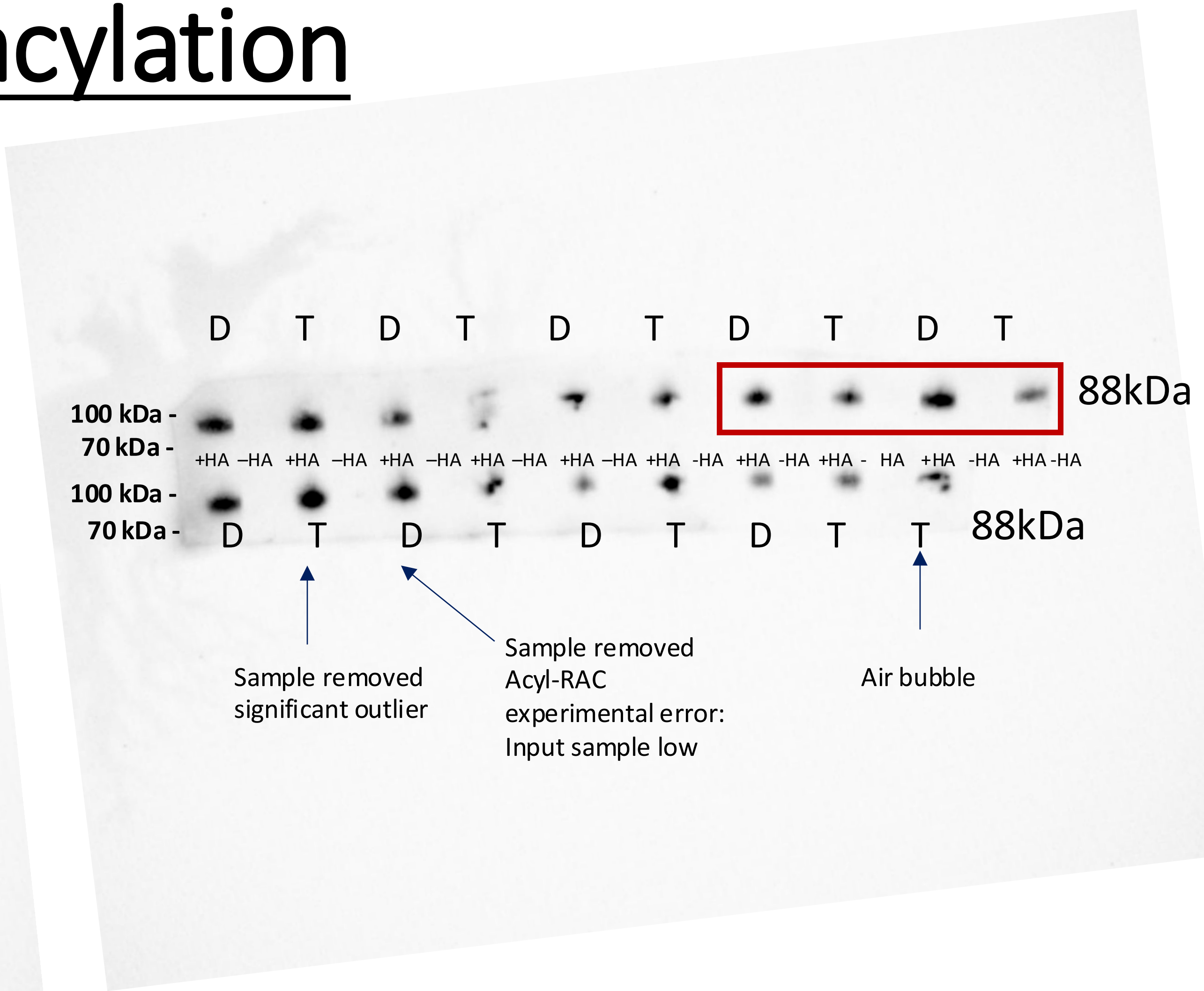

2 gels transferred on to one membrane – due to the positive (+HA) and negative (-HA) sample needed for every heart

Gels cut just above the 100 Mw band and below the 70 Mw band – then all transferred on to the same membrane, to minimize heterogeneity between samples through transfer, antibody incubation and developing.

Figure 6D: CMA Subcellular fractionation CD36 membrane 1/3

Chemi

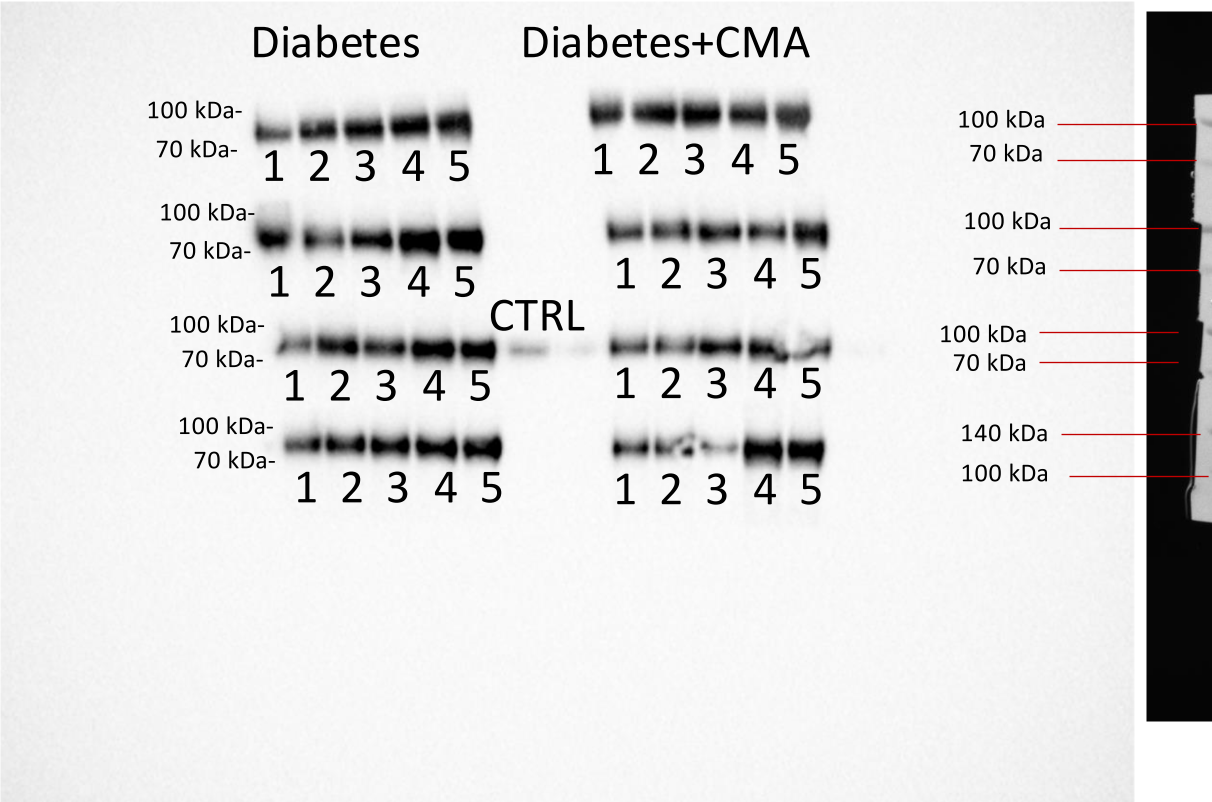

Marker

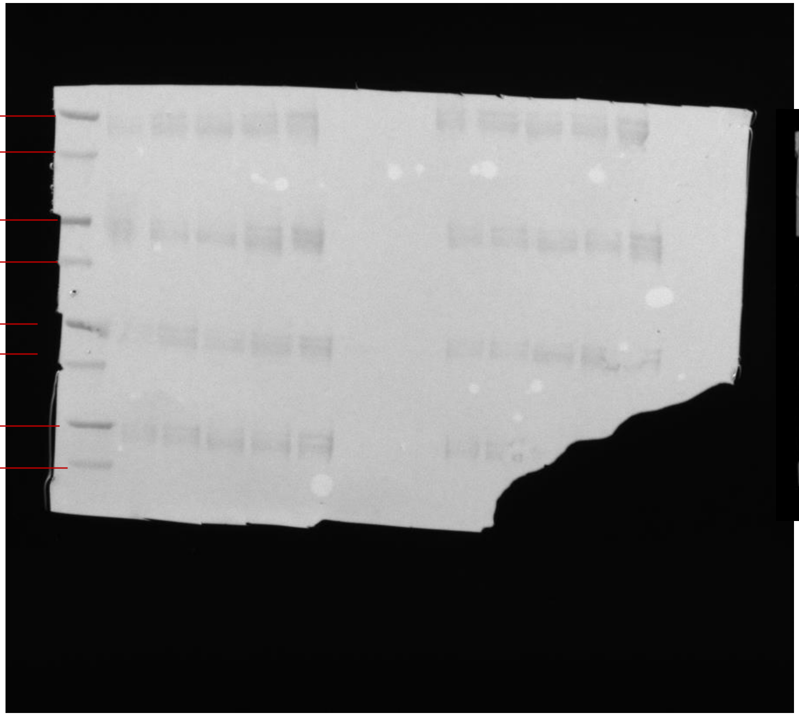

Ponceau

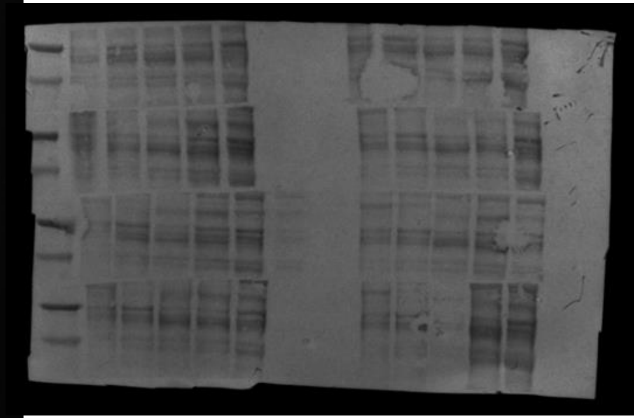

Membrane tore after chemiluminescence

4 gels transferred on to one membrane – due to the large number of fractions generated per heart.

Gels cut just above the 100 Mw band and below the 70 Mw band – then all transferred on to the same membrane, to minimize heterogeneity between samples through transfer, antibody incubation and developing.

Figure 6D: CMA Subcellular fractionation CD36 membrane 2/3

Chemi

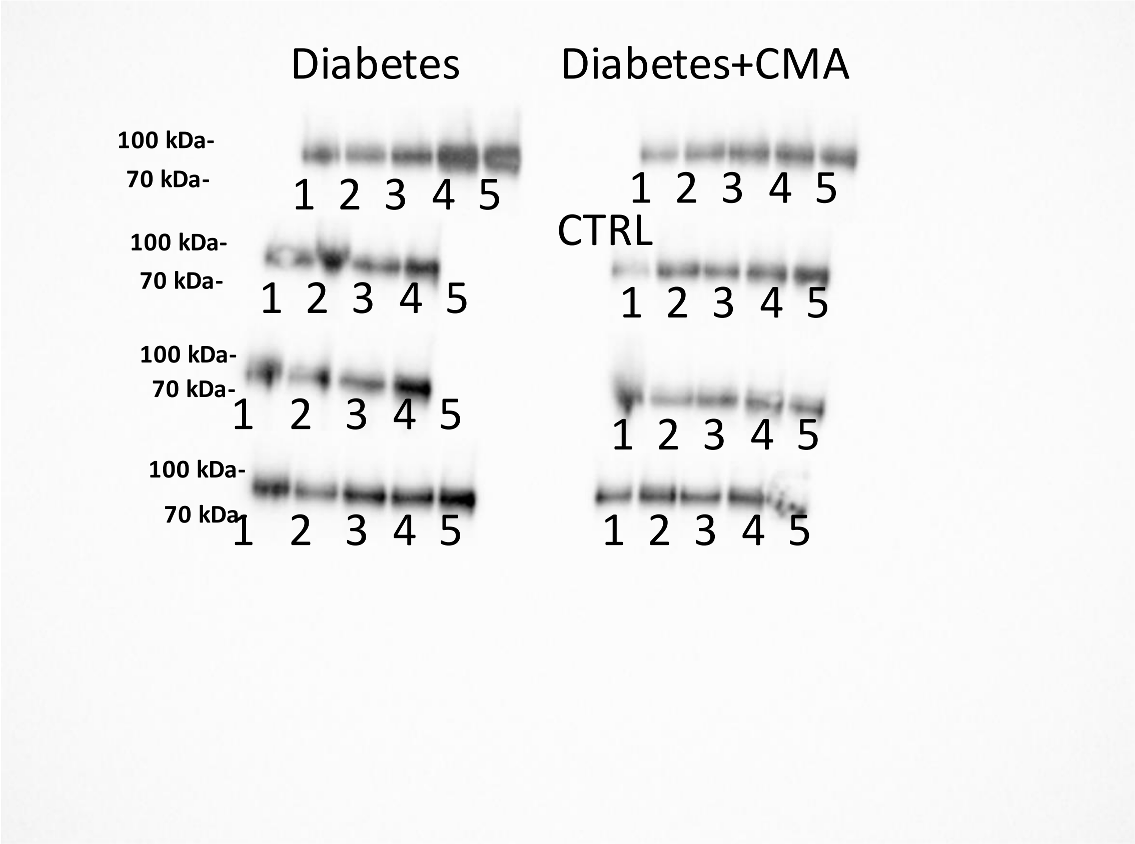

Markers

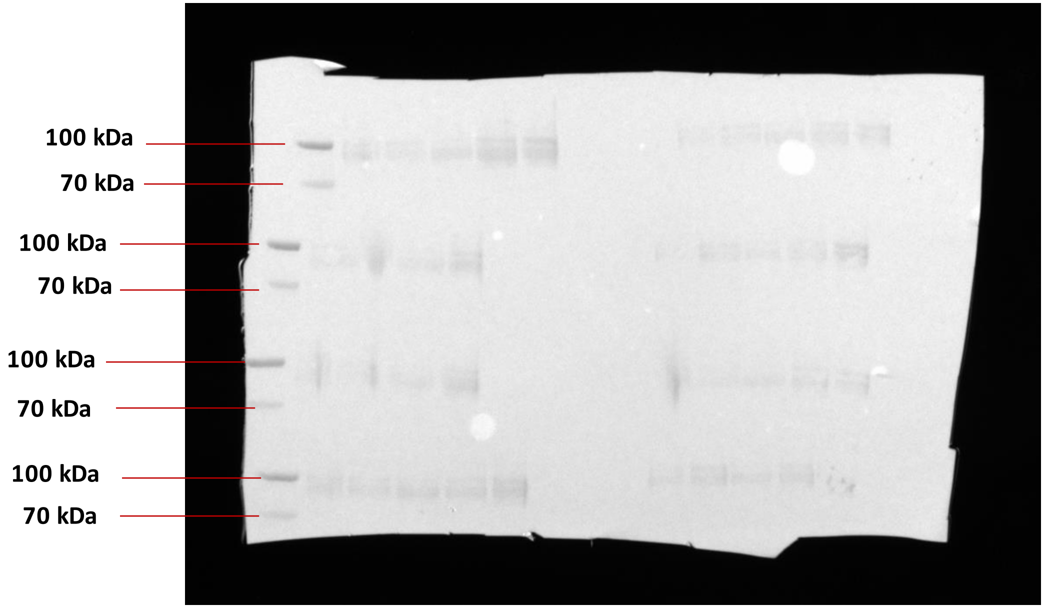

4 gels transferred on to one membrane – due to the large number of fractions generated per heart.

Gels cut just above the 100 Mw band and below the 70 Mw band – then all transferred on to the same membrane, to minimize heterogeneity between samples through transfer, antibody incubation and developing.

Figure 6D: CMA Subcellular fractionation CD36 membrane 3/3

Chemi

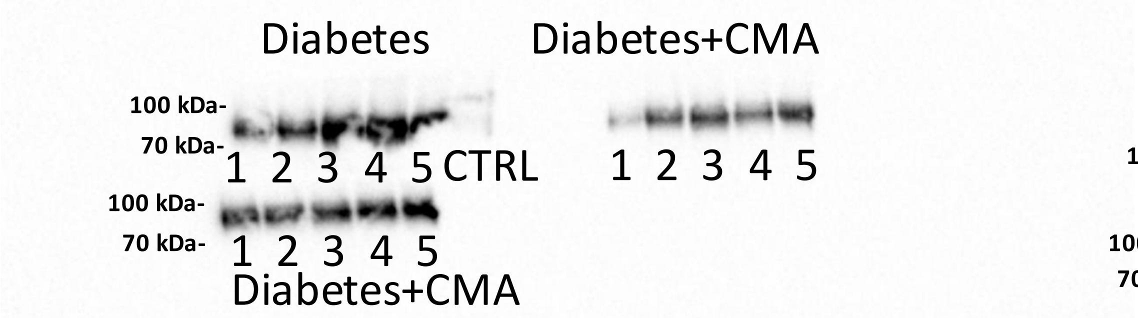

Markers

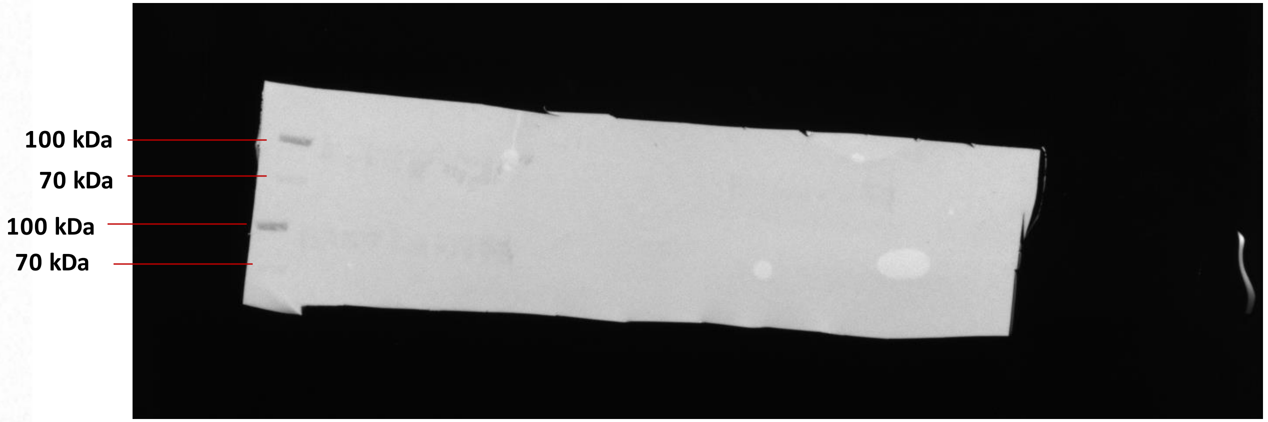

4 gels transferred on to one membrane – due to the large number of fractions generated per heart.

Gels cut just above the 100 Mw band and below the 70 Mw band – then all transferred on to the same membrane, to minimize heterogeneity between samples through transfer, antibody incubation and developing.

Figure 7B-C: ML348 CD36 S-acylation

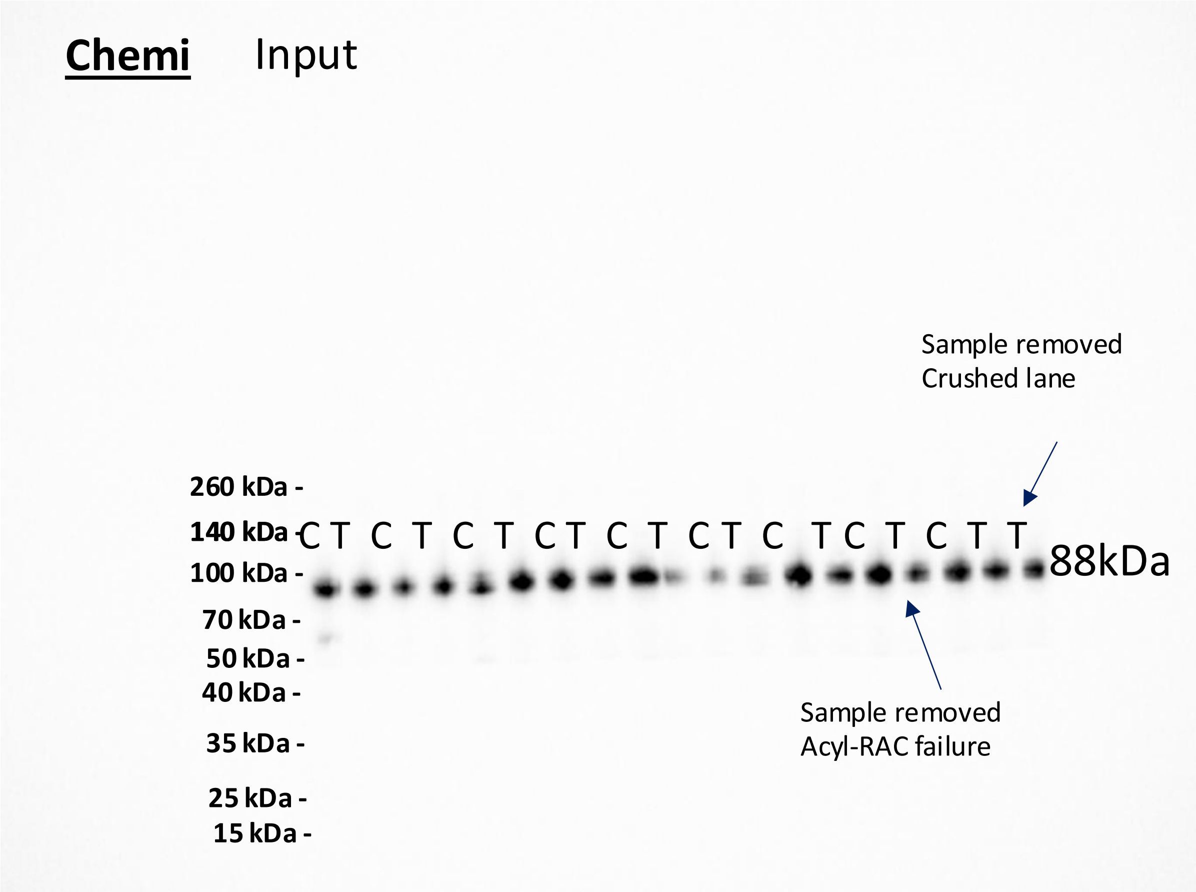

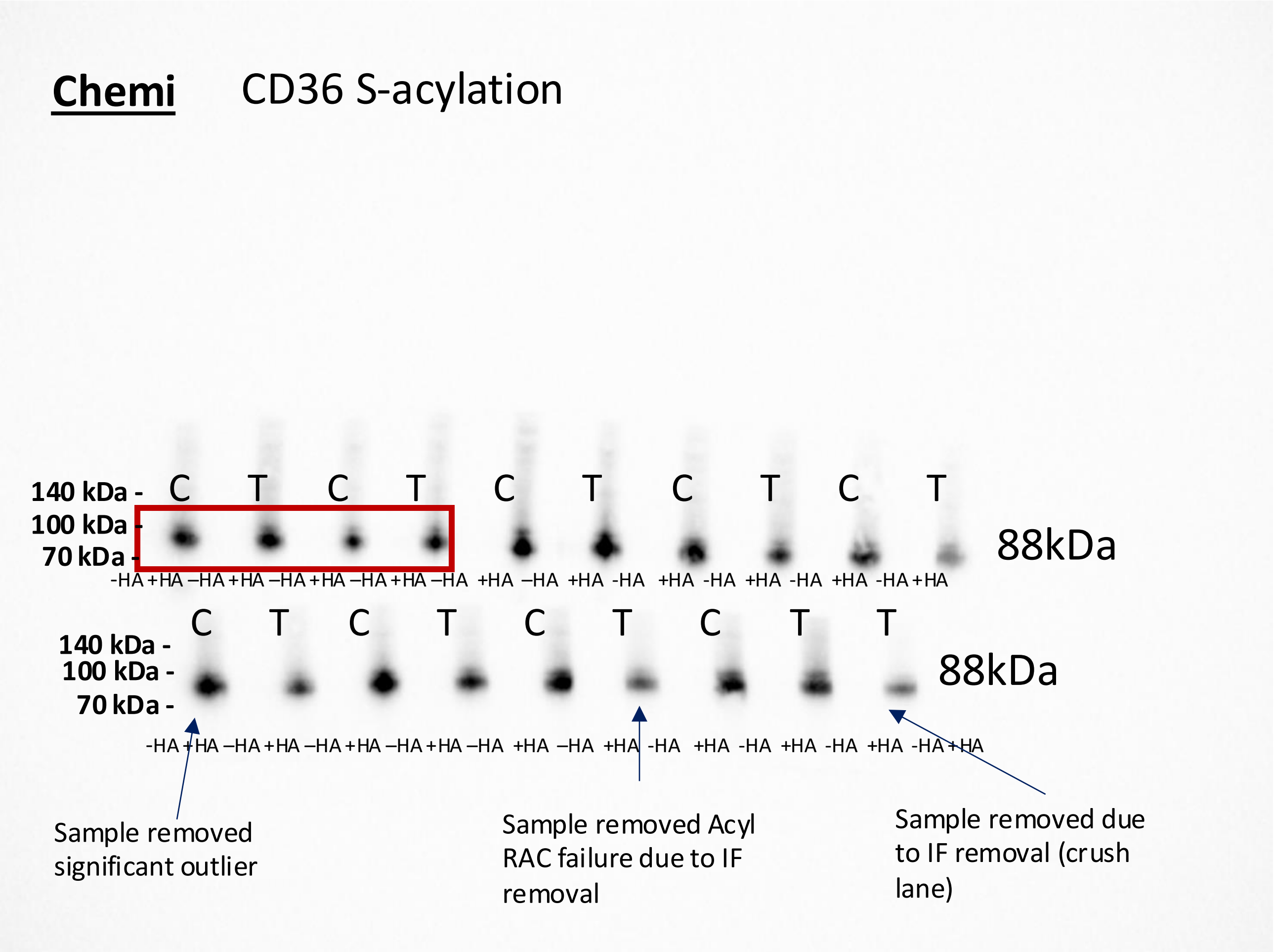

Uncropped gel and membrane

2 gels transferred on to one membrane – due to the positive (+HA) and negative (-HA) sample needed for every heart

Gels cut just above the 140 Mw band and below the 70 Mw band – then all transferred on to the same membrane, to minimize heterogeneity between samples through transfer, antibody incubation and developing.

Figure 7D: ML348 Subcellular fractionation CD36 membrane 1/2

Chemi

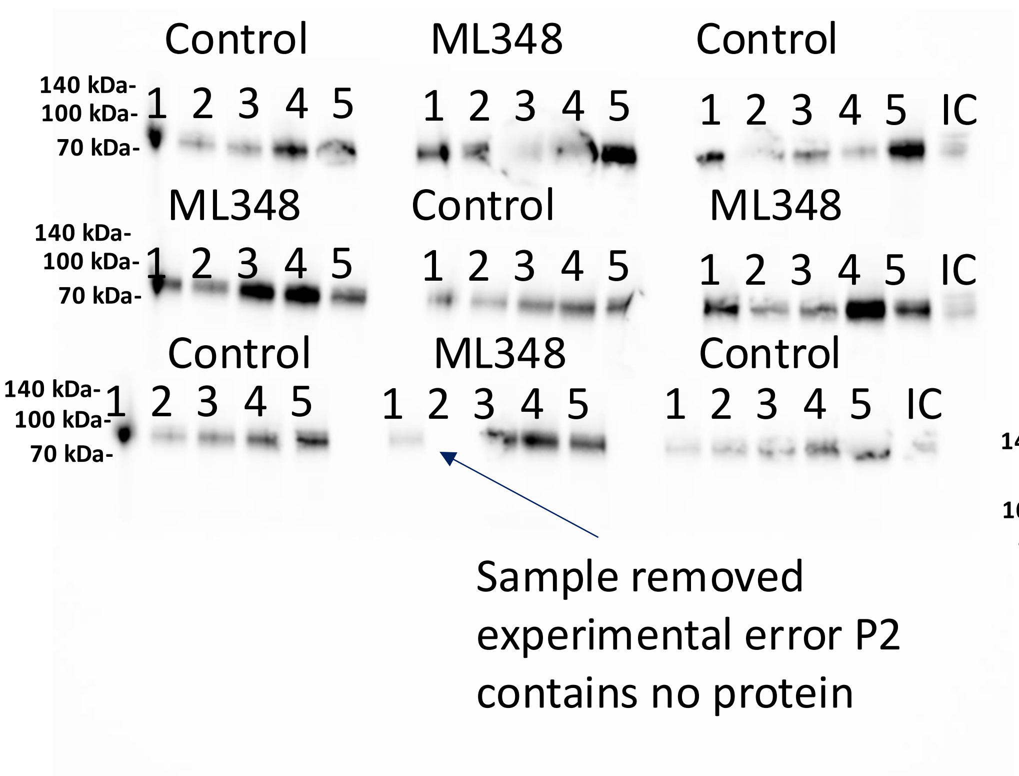

Marker

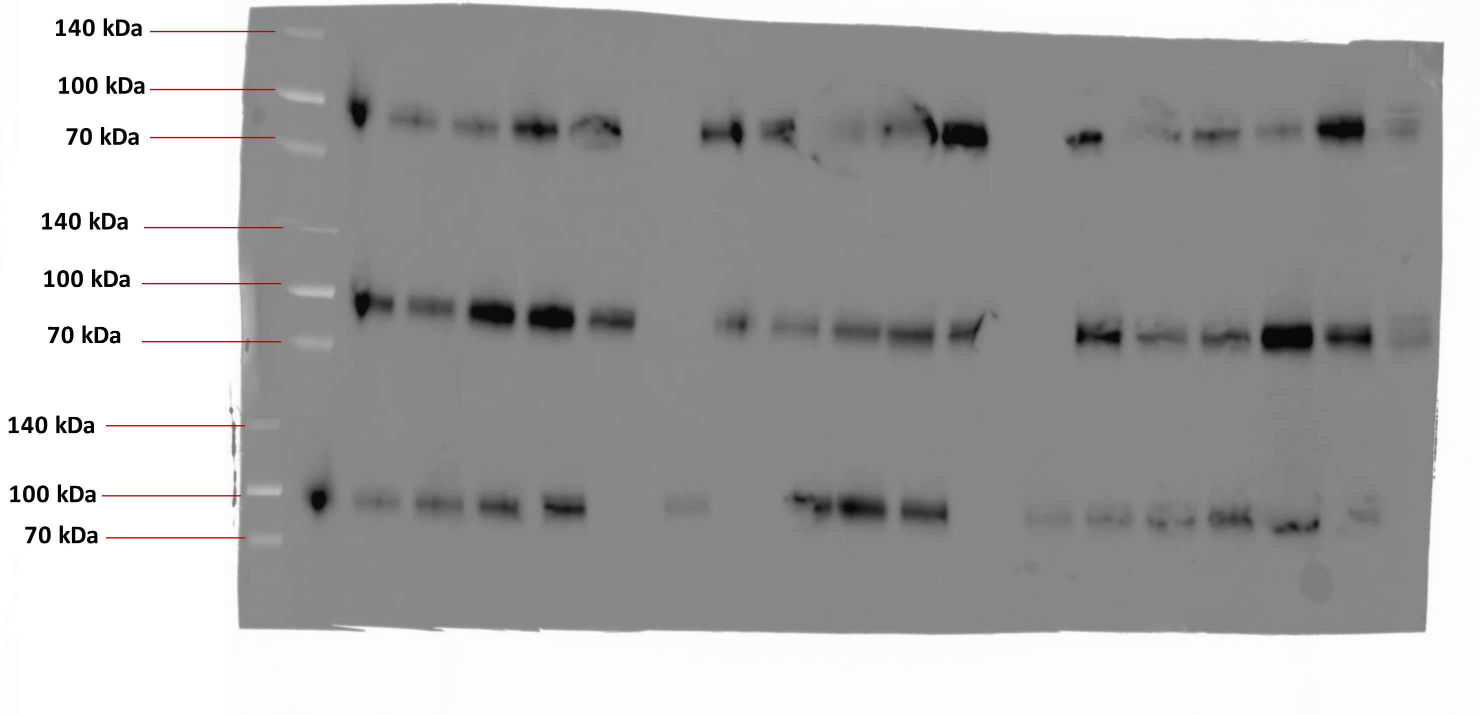

3 gels transferred on to one membrane – due to the large number of fractions generated per heart.

Gels cut just above the 140 Mw band and below the 70 Mw band – then all transferred on to the same membrane, to minimize heterogeneity between samples through transfer, antibody incubation and developing.

Figure 7D: ML348 Subcellular fractionation CD36 membrane 2/2

Chemi

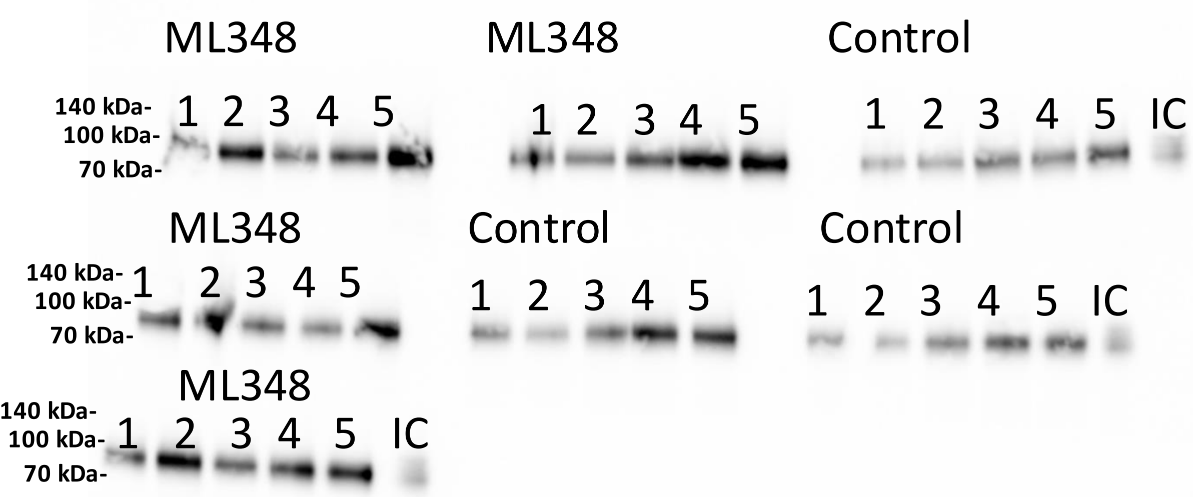

Marker

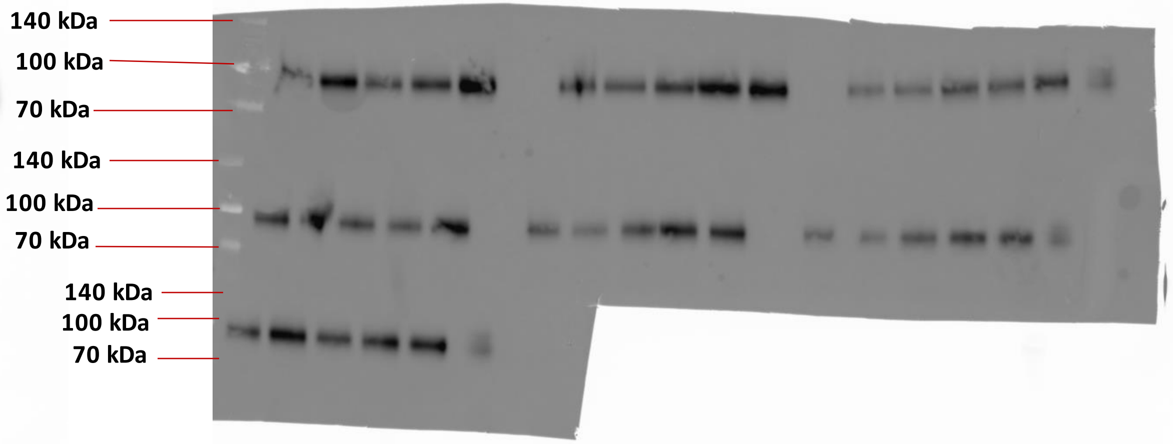

3 gels transferred on to one membrane – due to the large number of fractions generated per heart.

Gels cut just above the 140 Mw band and below the 70 Mw band – then all transferred on to the same membrane, to minimize heterogeneity between samples through transfer, antibody incubation and developing.

Supplemental Figure 2 A (CAV3 and CD36 validation - not quantified)

Chemi – CAV3

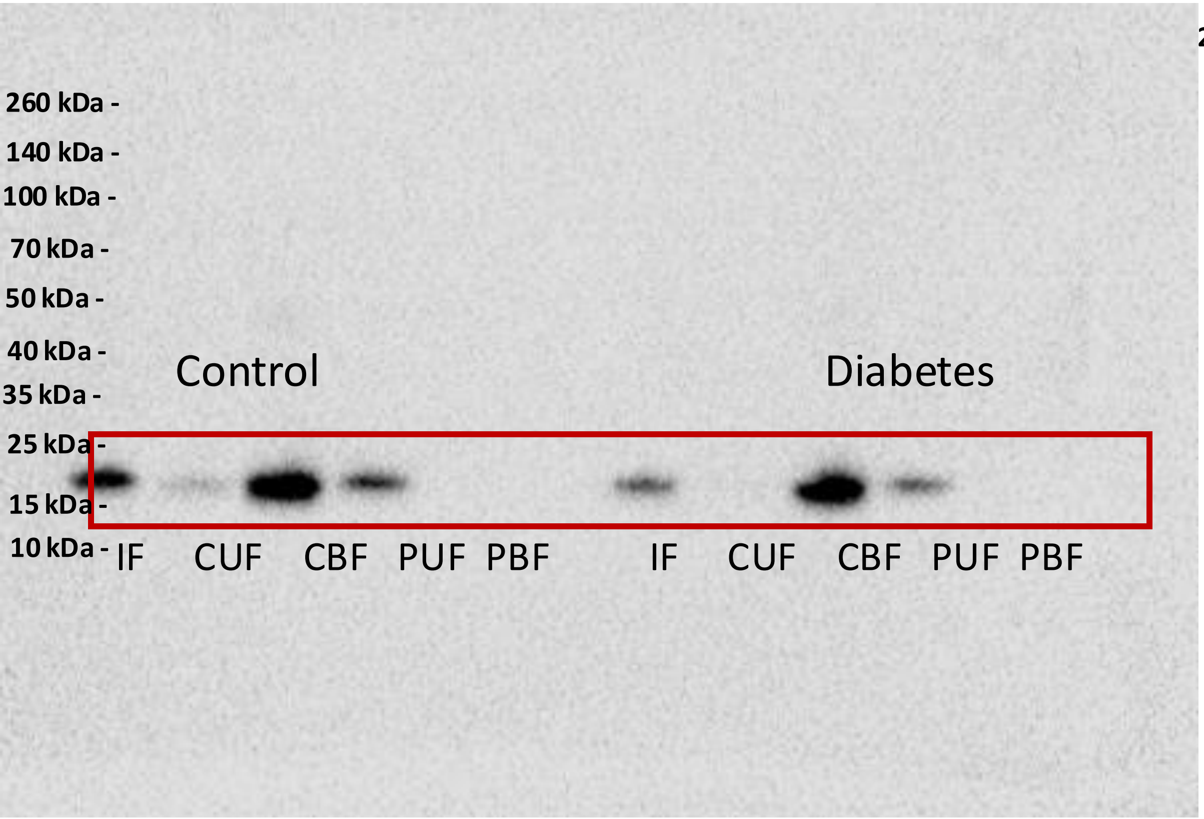

Chemi – CD36

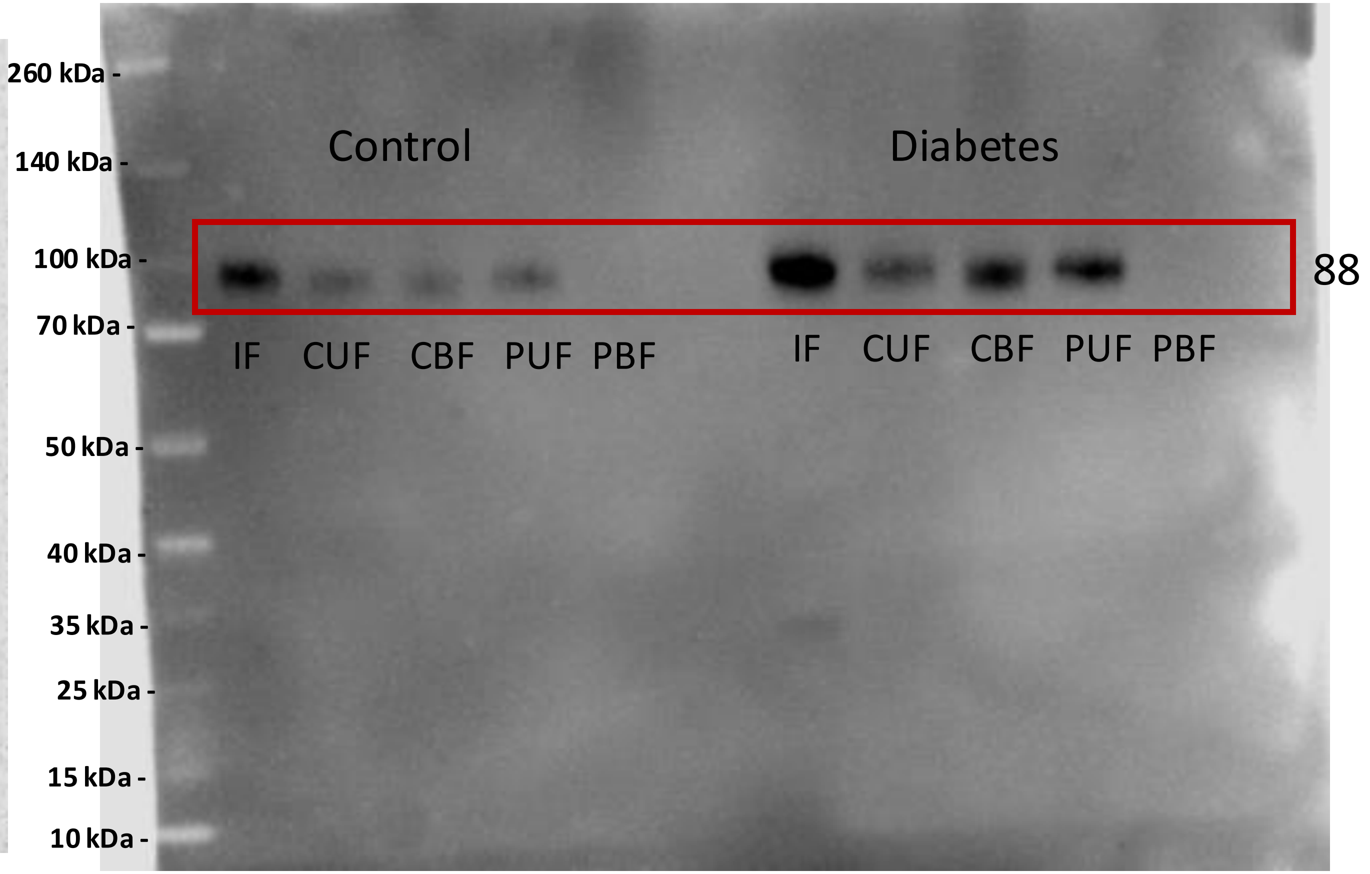

Uncropped gel and membrane – only one band for CD36

Supplemental Figure 2 B (not quantified) FABPpm

Chemi

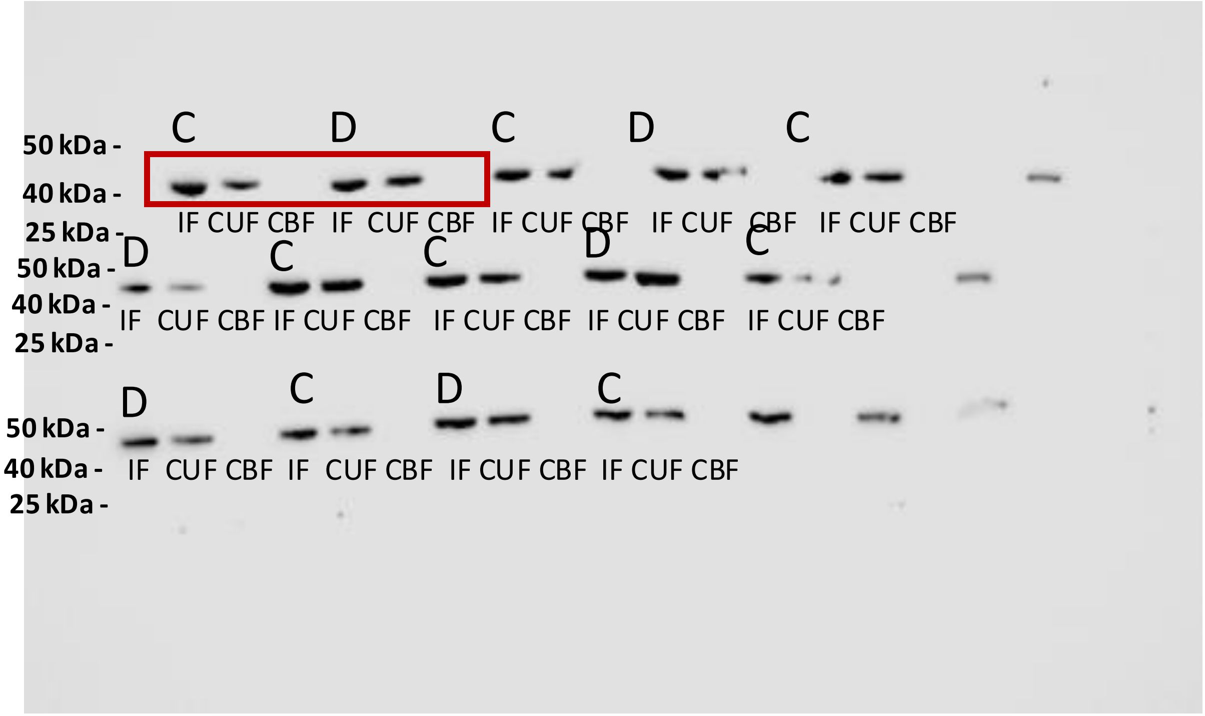

Marker

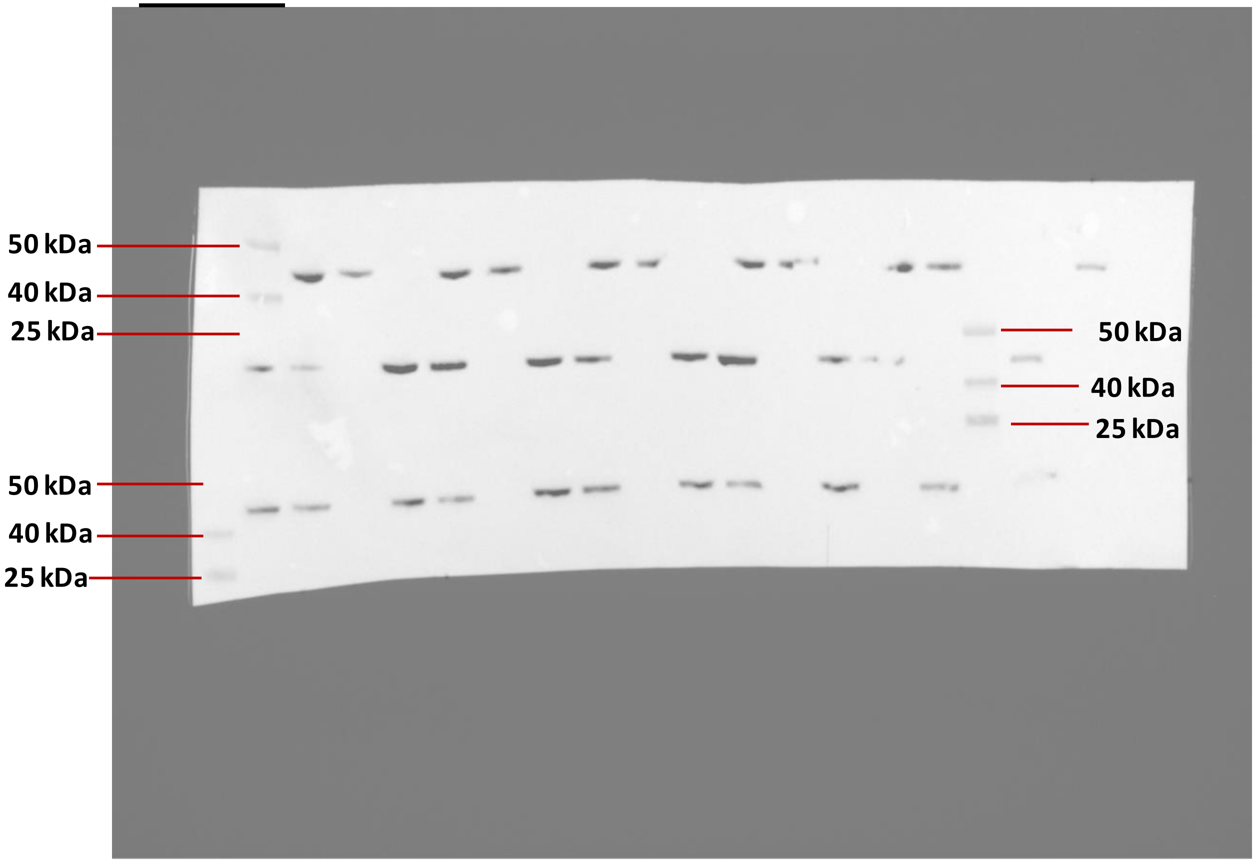

3 gels transferred on to one membrane – due to the positive (+HA) and negative (-HA) sample needed for every heart

Gels cut above the 50 kDa and below the 25 kDa then all transferred on to the same membrane, to minimize heterogeneity between samples through transfer, antibody incubation and developing.

Supplemental Figure 2 B (not quantified) GLUT1

Chemi

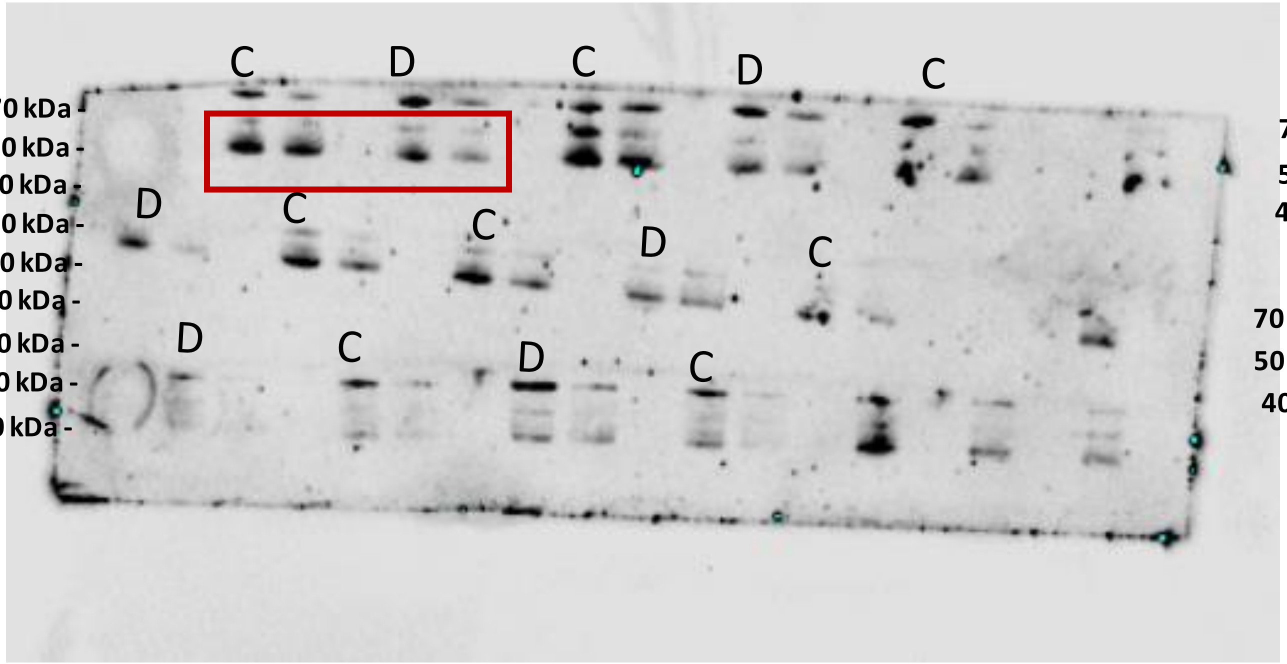

Marker

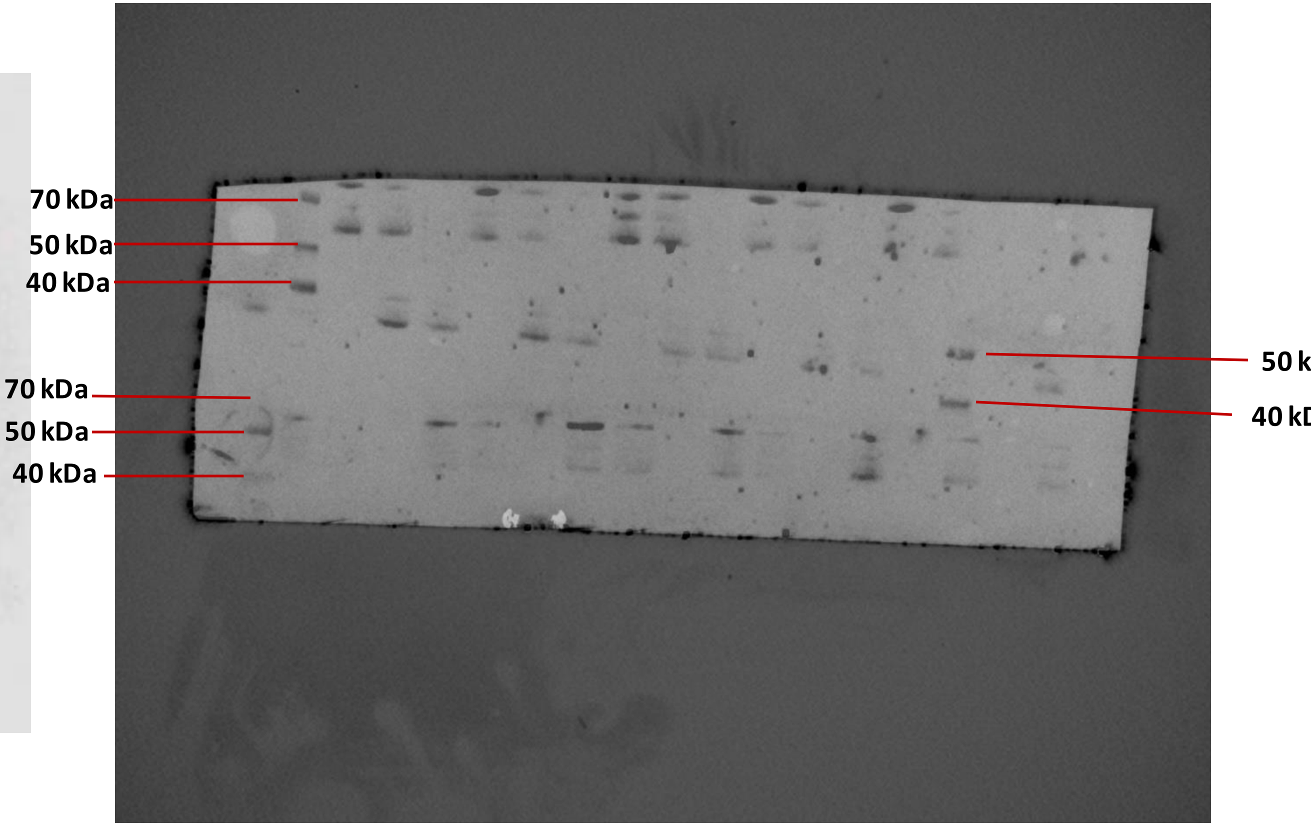

3 gels transferred on to one membrane – due to the positive (+HA) and negative (-HA) sample needed for every heart. Gels were cut above the 70 kDa and below the 40 kDa, then all transferred on to the same membrane, to minimize heterogeneity between samples through transfer, antibody incubation and developing.

Supplemental Figure 2 B (Not quantified)

Chemi CAV3

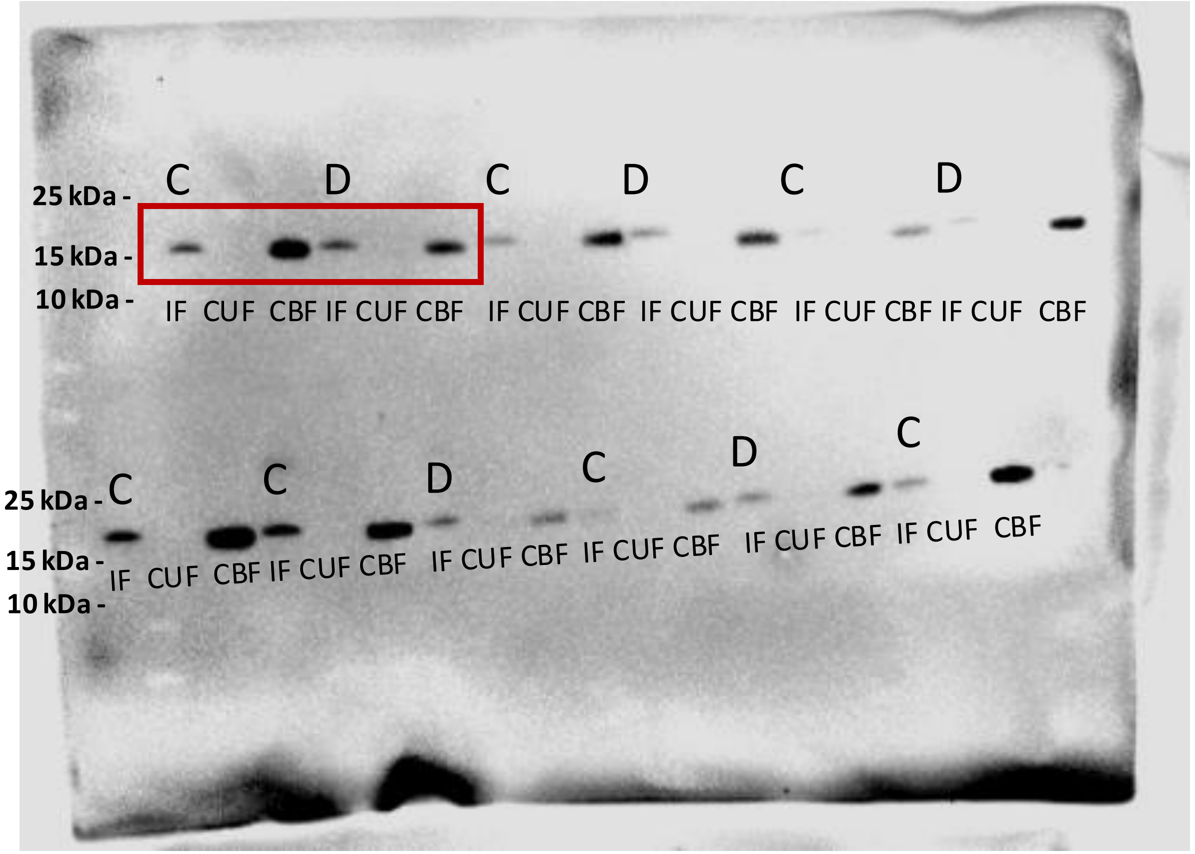

Chemi GLUT4

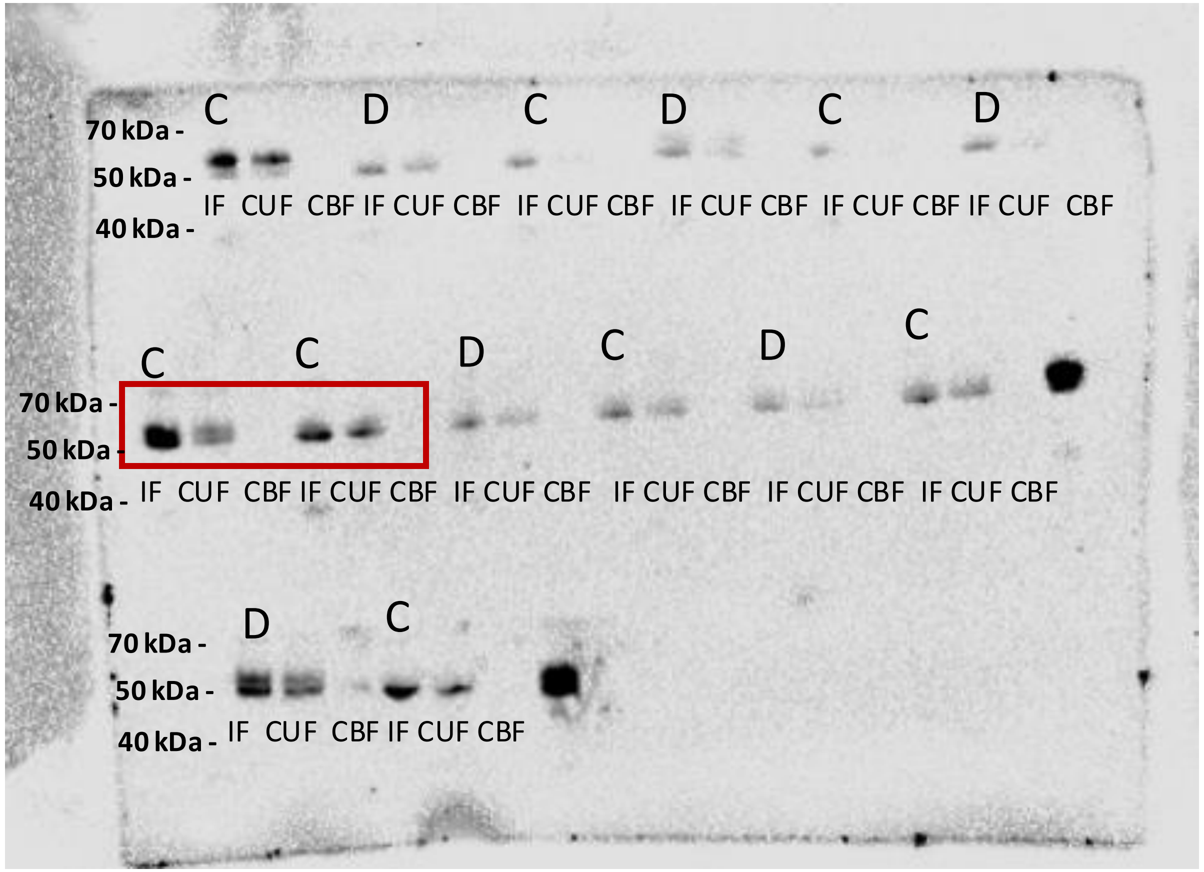

2-3 gels transferred on to one membrane – due to the positive (+HA) and negative (-HA) sample needed for every heart

Gels cut just above the 25 Mw band (for CAV3) and above the 70Mw band (for GLUT4) – then gels transferred on to the same membrane, to minimize heterogeneity between samples through transfer, antibody incubation and developing.

Supplemental Figure 2 C - CAV3

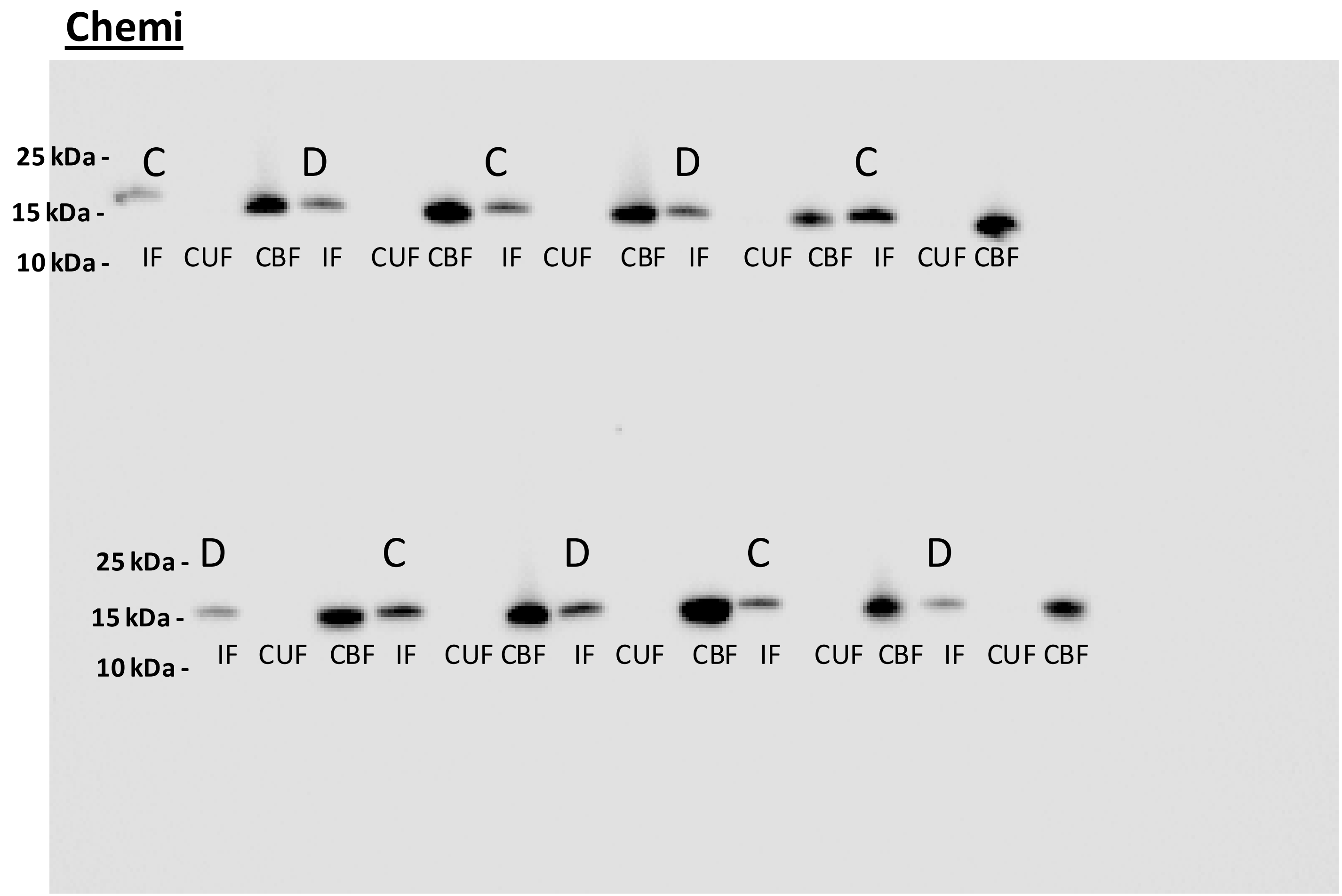

2 gels transferred on to one membrane – due to the positive (+HA) and negative (-HA) sample needed for every heart

Gels cut just above the 25 Mw band and below the 10 Mw band – then all transferred on to the same membrane, to minimize heterogeneity between samples through transfer, antibody incubation and developing.

Supplemental Figure 3 A-B: CAV3 S-acylation

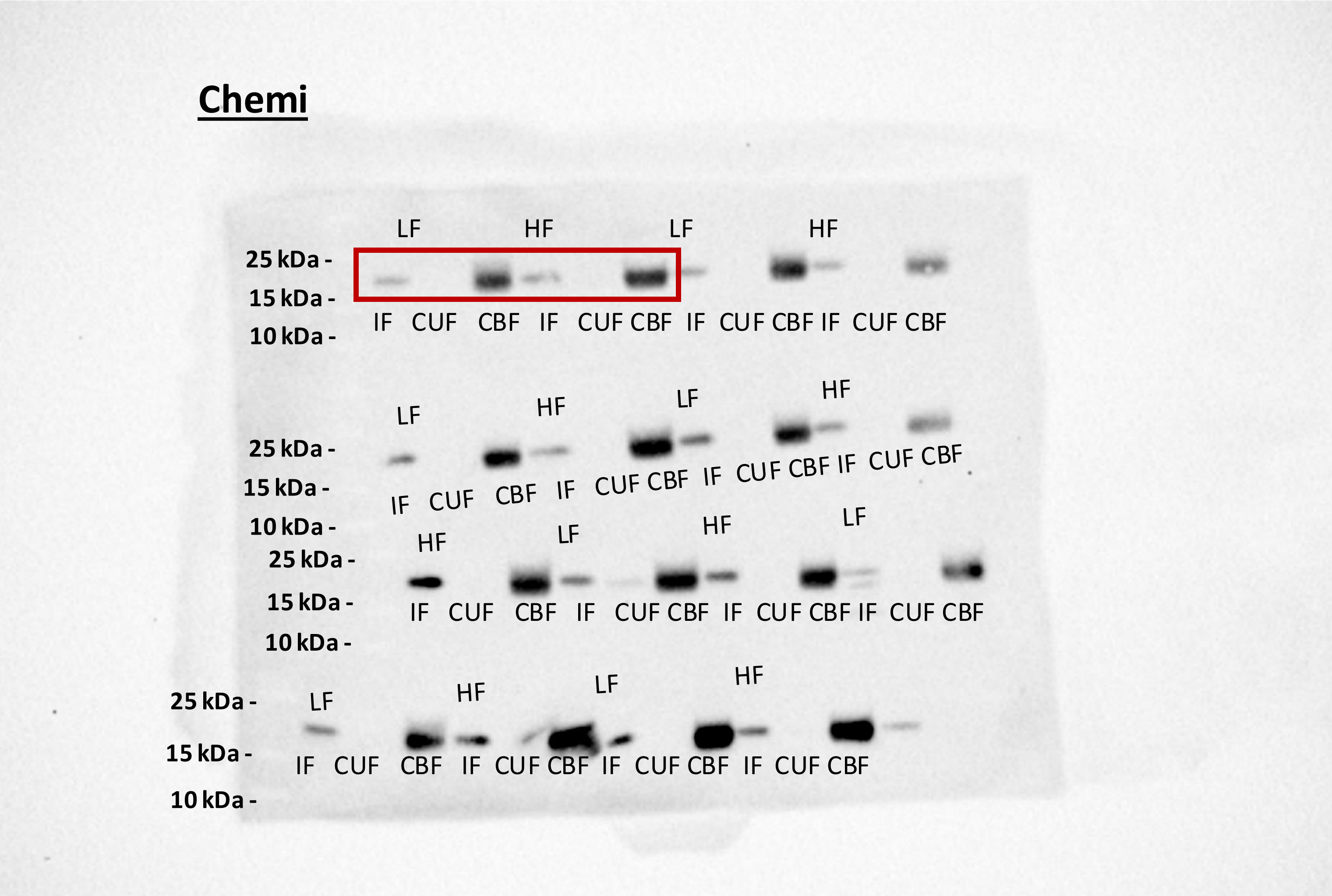

4 gels transferred on to one membrane – due to the positive (+HA) and negative (-HA) sample needed for every heart

Gels cut just above the 25 Mw band – then all transferred on to the same membrane, to minimize heterogeneity between samples through transfer, antibody incubation and developing.

Supplemental Figure 4A-B : CAV3 S-acylation

Chemi Input

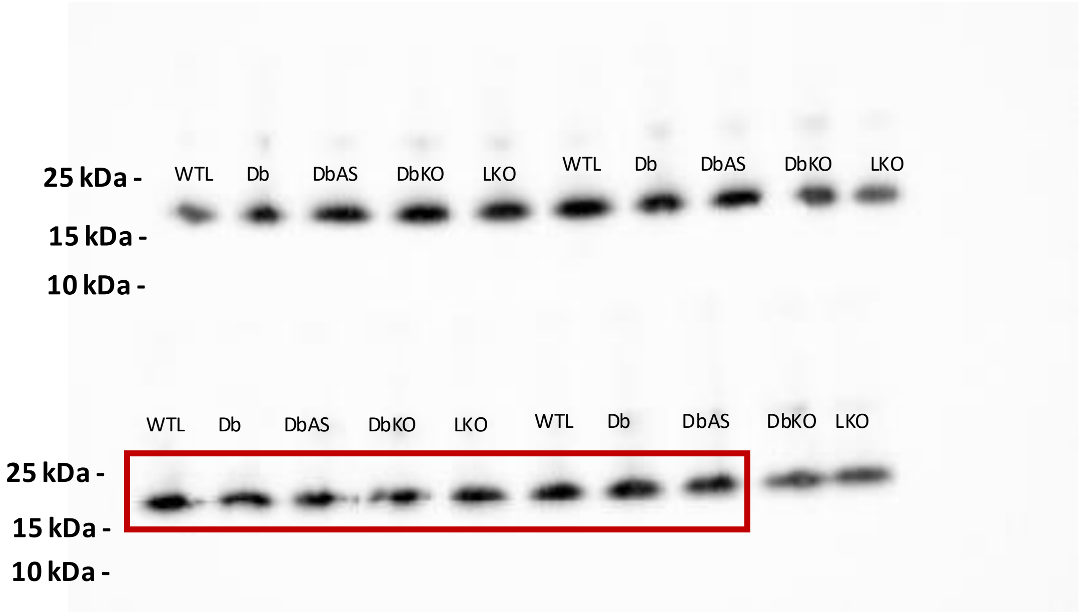

Input (two gels cut (just below 10 kDa and above 25 kDa) and transferred on the same membrane) from the same CD36 gels

Chemi

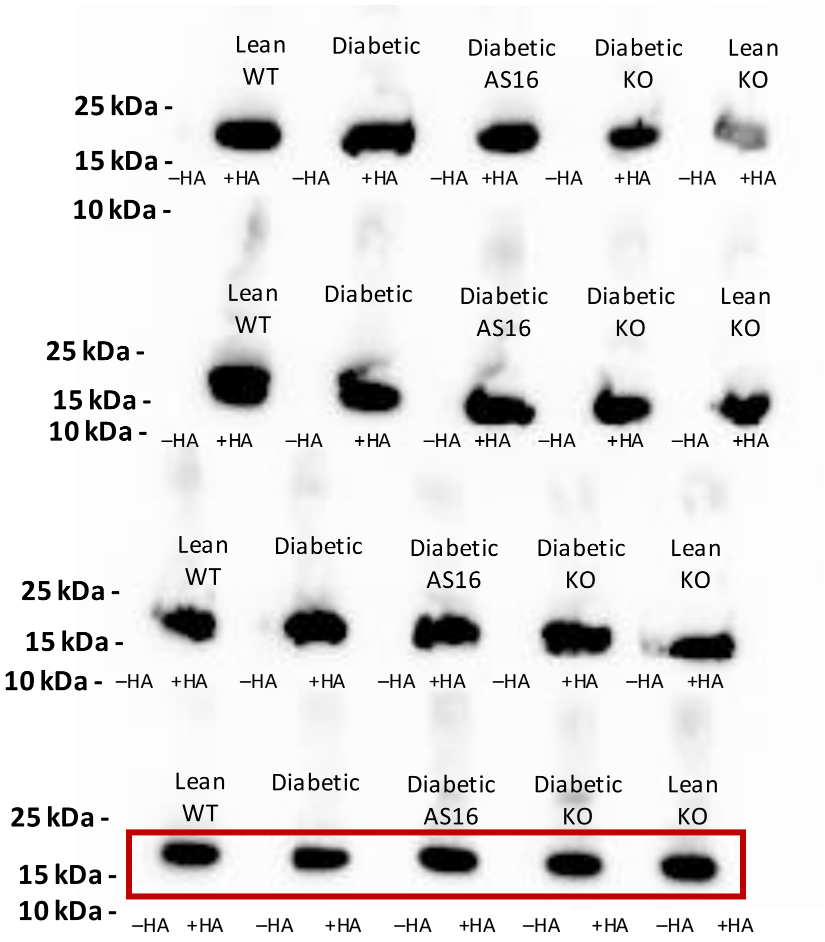

Four gels transferred on to one membrane – due to the positive (+HA) and negative (-HA) sample needed for every heart. Gels cut just above the 25 Mw band and below the 10Mw band – then all transferred on to the same membrane, to minimize heterogeneity between samples through transfer, antibody incubation and developing.

Supplemental Figure 5A-B : CAV3 S-acylation

Chemi Input

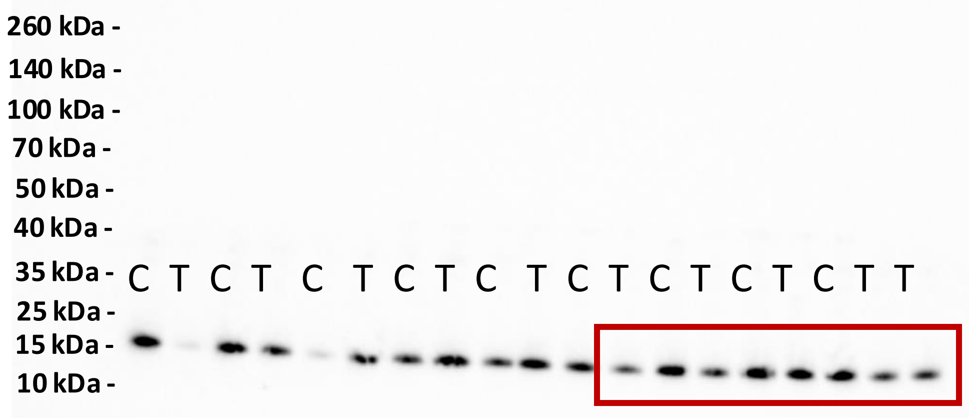

Uncropped gel and membrane

Chemi

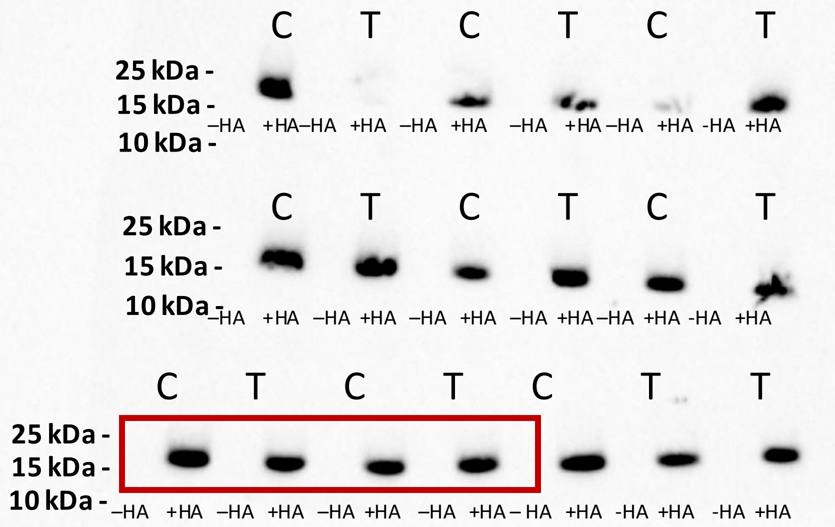

Three gels transferred on to one membrane – due to the positive (+HA) and negative (-HA) sample needed for every heart

Gels cut just above the 25 Mw band and below the 10Mw band – then all transferred on to the same membrane, to minimize heterogeneity between samples through transfer, antibody incubation and developing.

Supplemental Figure 6A-B : CAV3 S-acylation

Chemi Input

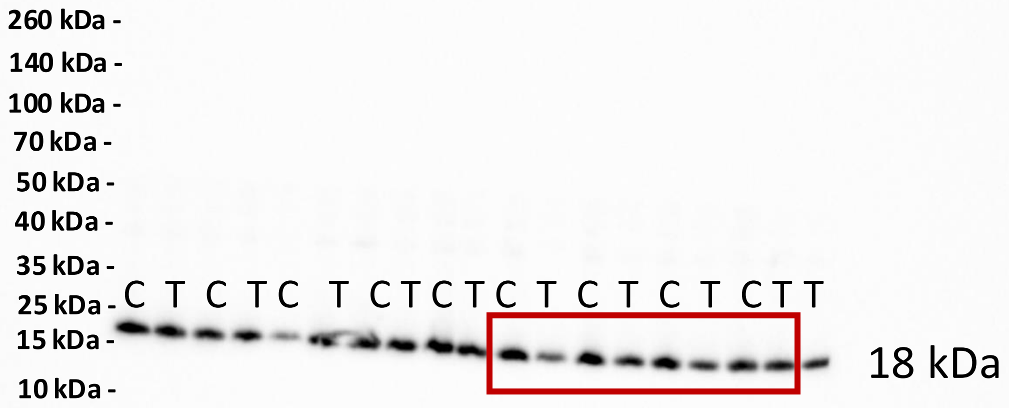

Uncropped gel and membrane

Chemi

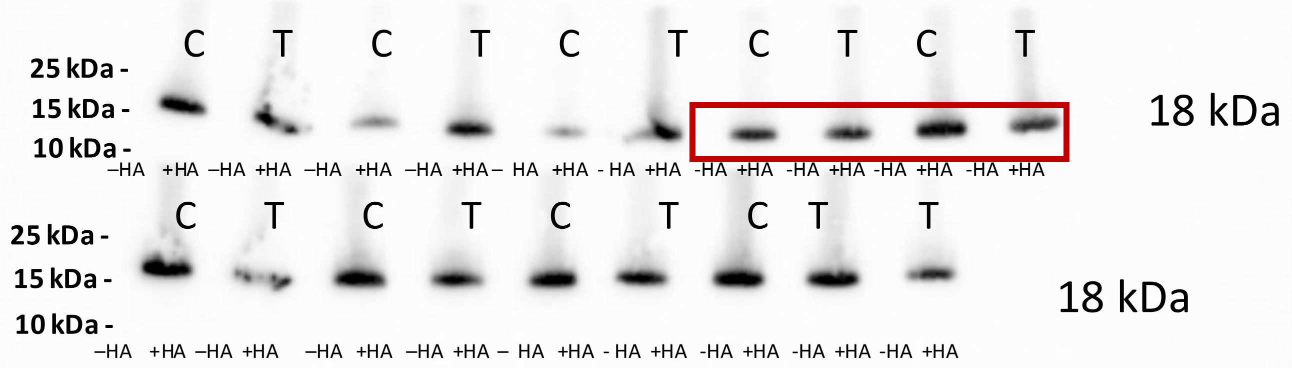

2 gels transferred on to one membrane – due to the positive (+HA) and negative (-HA) sample needed for every heart

Gels cut just above the 25 Mw band and below the 10Mw band – then all transferred on to the same membrane, to minimize heterogeneity between samples through transfer, antibody incubation and developing.
